# Inositol is an effective and safe treatment in polycystic ovary syndrome: a systematic review and meta-analysis of randomized controlled trials

**DOI:** 10.1186/s12958-023-01055-z

**Published:** 2023-01-26

**Authors:** Dorina Greff, Anna E. Juhász, Szilárd Váncsa, Alex Váradi, Zoltán Sipos, Julia Szinte, Sunjune Park, Péter Hegyi, Péter Nyirády, Nándor Ács, Szabolcs Várbíró, Eszter M. Horváth

**Affiliations:** 1 Centre for Translational Medicine, Semmelweis University, Budapest, Hungary; 2 Department of Obstetrics and Gynecology, Semmelweis University, 1182 Budapest, Üllői Út 78/A, Hungary; 3 Department of Physiology, Semmelweis University, Budapest, Hungary; 4 Department of Dietetics and Nutrition Sciences, Semmelweis University, Budapest, Hungary; 5 Institute for Translational Medicine, Szentágothai Research Centre, Medical School, University of Pécs, Pécs, Hungary; 6 Institute of Pancreatic Diseases, Semmelweis University, Budapest, Hungary; 7 Department of Urology, Semmelweis University, Budapest, Hungary; 8 Workgroup for Science Management, Doctoral School, Semmelweis University, Budapest, Hungary

**Keywords:** Cycle length, Testosterone, Insulin, BMI, Metabolic syndrome, PCOS, Inositol, Metformin

## Abstract

**Background:**

Metformin is the gold standard insulin sensitizer, which is widely used to treat insulin resistance in polycystic ovary syndrome (PCOS). However, metformin may induce gastrointestinal side effects.

**Objective:**

Inositols have long been debated as a potential alternative for metformin in treating PCOS. Therefore, the present systematic review aimed to evaluate the efficacy and safety of inositols in treating PCOS.

**Methods:**

The present systematic search was performed in CENTRAL, MEDLINE, and Embase from the inception until October 20th, 2021. Eligible randomized controlled trials (RCTs) included women diagnosed with PCOS and compared any inositols with metformin or placebo. Our primary outcome was cycle normalization, whereas secondary outcomes were body mass index (BMI), parameters of carbohydrate metabolism and clinical and laboratory hyperandrogenism. Results are reported as risk ratios or mean differences (MDs) with 95% confidence intervals (CIs).

**Results:**

Twenty-six RCTs were identified, including data of 1691 patients (806 inositol, 311 with placebo, and 509 metformin groups). In patients treated with inositols, the risk (CI: 1.13; 2.85) of having a regular menstrual cycle was found by 1.79 higher than in the case of placebo. Moreover, the inositols showed non-inferiority compared to metformin in this outcome. In the case of BMI (MD** = **-0.45; CI: -0.89; -0.02), free testosterone (MD = -0,41, CI: -0.69; -0.13), total testosterone (MD = -20.39, CI: -40.12; -0.66), androstenedione (MD** = **-0.69, CI: -1,16; -0.22), glucose (MD = -3.14; CI: -5.75; -0.54) levels and AUC insulin (MD = -2081.05, CI: -2745.32; -1416.78) inositol treatment induced greater decrease compared to placebo. Inositol increased sex-hormone-binding globulin significantly compared to placebo (MD = 32.06, CI:1.27; 62.85).

**Conclusion:**

Inositol is an effective and safe treatment in PCOS. Moreover, inositols showed non-inferiority in most outcomes compared to the gold standard treatment; metformin.

**Trial registration:**

PROSPERO registration number: CRD42021283275.

**Supplementary Information:**

The online version contains supplementary material available at 10.1186/s12958-023-01055-z.

## Background

Polycystic ovary syndrome (PCOS) is the most common endocrine disorder and one of the most frequent causes of infertility in women [1]. It affects 5–20% of women of childbearing age [1, 2]. Diagnosing PCOS is challenging due to the variability of symptoms [3]. On the basis of the latest clinical guideline, PCOS should be diagnosed according to the Rotterdam criteria, meaning the presence of at least two of the following criteria: ovulatory dysfunction, hyperandrogenism, or polycystic ovary morphology [4].

The pathogenesis of PCOS is still not fully understood. On the other hand, insulin resistance (IR) has a central role in its pathogenesis [5–7]. According to a cross-sectional study, IR is present in 75% of lean and 95% of overweight women with PCOS [8]. It is important to emphasize that 60–70% of women with PCOS are overweight [9]. Moreover, IR is more severe in obese women [7]. IR and compensatory hyperinsulinemia can, directly and indirectly, lead to irregular menstrual cycles and hyperandrogenism. Higher insulin levels reduce the sex hormone binding globulin (SHBG) production of the liver. Reduced SHBG levels lead to increased free testosterone levels worsening the symptoms of hyperandrogenism. In addition, hyperinsulinemia stimulates the androgen overproduction of ovarian theca cells [10].

In the treatment of PCOS, metformin is the gold standard metabolic treatment [4, 10]. However, metformin may induce mild to severe gastrointestinal side effects such as nausea, diarrhea, vomiting, and flatulence [11]. Therefore, alternative treatment with fewer side effects would be beneficial in managing these patients. In recent years, several studies have analyzed the potential effects of inositol supplementation, suggesting that inositols are potent alternatives for metformin in treating PCOS [12–16].

Inositols belong to the vitamin B complex group, which is synthesized in the human body. There are nine stereoisomers, of which the most important ones are myoinositol and D-chiro-inositol [17, 18]. Inositols are considered insulin sensitizers, as they modulate the members of insulin signaling pathways [6]. They positively influence menstrual cycle regularity, carbohydrate metabolism, and the clinical and laboratory symptoms of hyperandrogenism (e.g., free testosterone, total testosterone, SHBG) [19]. However, to date, the level of evidence has not been satisfactory for accepting them as standard therapy in the guidelines [4].

Thus, the aim of the present study was to systematically review the available randomized controlled trials (RCTs) regarding the efficacy and safety of inositols in treating PCOS, providing evidence for the following guidelines in this respect.

## Methods

The present systematic review and meta-analysis was carried out conclusively with the PRISMA 2020 guideline [20] (see Table S1), while the Cochrane Handbook was followed [21]. The study protocol was registered on PROSPERO (registration number CRD42021283275). Due to the lack of data, the primary outcome was changed from the presence of ovulation and menstrual cycle length to menstrual cycle normalization. In addition, the minimum number of studies for the meta-analysis was decreased to two.

### Eligibility criteria

RCTs were included comparing the efficacy and safety of inositols to placebo or metformin in women with PCOS without age restriction. In eligible studies, PCOS was diagnosed according to the Rotterdam criteria [22]. However, studies that did not mention the Rotterdam criteria, but diagnosed PCOS based on corresponding criteria, were also included. The intervention was any inositol in monotherapy, or inositol in combination with dietary supplements or aromatase inhibitors regardless of the dosage and duration of the treatment. Comparators were placebo (C1) or metformin (C2) in monotherapy; or placebo or metformin in combination (C3) with dietary supplements or aromatase inhibitors.

The primary outcome was the improvement of ovarian function determined by the rate (number of women with normal menstrual cycle in the study groups) of menstrual cycle normalization. Secondary outcomes were pregnancy rate (number of pregnancies in the study groups), body mass index (BMI), carbohydrate metabolism (fasting glucose, fasting insulin, oral glucose tolerance test—OGTT, Homeostatic Model Assessment insulin resistance – HOMA-IR index), clinical and laboratory hyperandrogenism (hirsutism, testosterone, androstenedione, dehydroepiandrosterone-sulfate – DHEAS, SHBG), and the side effects of the treatment.

The following studies were excluded: (1) cohort, case–control, case reports, cross-sectional studies, reviews, and animal studies, (2) studies with a combination of inositols and metformin therapy, and (3) studies reporting on pregnant women.

### Information sources and search strategy

The systematic search was performed in MEDLINE (via PubMed), Embase, and Cochrane Central Register of Controlled Trials (CENTRAL) from the inception until October 20^th^, 2021. In addition, the reference list of the studies was screened for further eligible RCTs.

The systematic search was carried out with the following predefined search key: (PCOS OR PCOD OR polycystic ovar* disease OR "polycystic ovary syndrome" OR polycystic ovar* syndrom*) AND (inositol OR inositols OR metformin OR myoinositol OR chiroinositol). Filters or language restrictions were not applied during the search.

### Selection process

Two independent review authors selected the articles via the EndNote X9 (Clarivate Analytics, Philadelphia, PA, USA) reference manager program. Publications were screened based on title, and abstract first, and then the full text based on the eligibility criteria. A third independent review author resolved disagreements during the selection process.

### Data collection process and data items

A standardized data collection sheet was created based on the consensus of methodological and clinical experts. Then, two independent review authors extracted data from the eligible articles using the standardized data collection sheet.

The following data were extracted: title, first author, year of publication, countries, number of centers, study design, main study findings, patient demographics, inclusion and exclusion criteria, details regarding the PICO (population, intervention, comparator, outcome), and the event rates or the means of outcomes in the examined groups.

For continuous variables, baseline and after treatment mean and standard deviation (SD) values were extracted, and in the case of missing SD p-values from paired t-test were collected as well.

For dichotomous data, events for the outcomes and total numbers of patients were used on both arms.

### Study risk of bias assessment

The risk of bias was assessed based on the recommendation of the Cochrane Collaboration, using the Cochrane risk-of-bias tool for randomized trials (RoB 2) [23]. Disagreements between the data extractors were resolved by involving a third reviewer.

### Synthesis methods

The effect of inositol treatment compared to placebo or metformin was analyzed. If possible, subgroup analysis was carried out based on different inositol isomers and their combinations (D-chiro-inositol, myoinositol, or a combination of the two).

The continuous results were presented by calculating mean differences (MD) with 95% confidence intervals (CIs) for continuous variables from the changes between the baseline and after treatment value. Because of missing correlation of before and after values, a 0 correlation was assumed to calculate the SD of change. In the case of missing SD and presence of p-value, the recommendation of Cochrane handbook was followed [24] To pool MDs, the random-effects model was applied with inverse variance method, and Restricted maximum-likelihood method was used to estimate variance measure τ^2^ [25]. In the case of dichotomous categorical outcomes, pooled risk ratios (RRs) were calculated with 95% CIs using the random-effects model with the Mantel–Haenszel method, and to obtain τ^2^ the Paule-Mandel estimator was used [26].

In all models, p-value less than 0.05 was considered to be statistically significant. Statistical heterogeneity was assessed by the I^2^ statistics and the Cochran Q test, where *p* < 0.1 indicates significant heterogeneity. Where applicable, the prediction intervals (i.e. the expected range of effects of future studies) of the results were reported following the recommendations of IntHout et al. [27]. All the results were summarized graphically on forest plots. To pool MDs, metacont was used, and for RR metabin functions from the meta R package v. 5.5–0 [28] . All statistical calculations were done using the R language [29].

### Assessing the level of evidence

The recommendation of the "Grades of Recommendation, Assessment, Development, and Evaluation (GRADE)" workgroup was followed to evaluate the quality of evidence [30].

## Results

### Search and selection

**Fig. 1:**
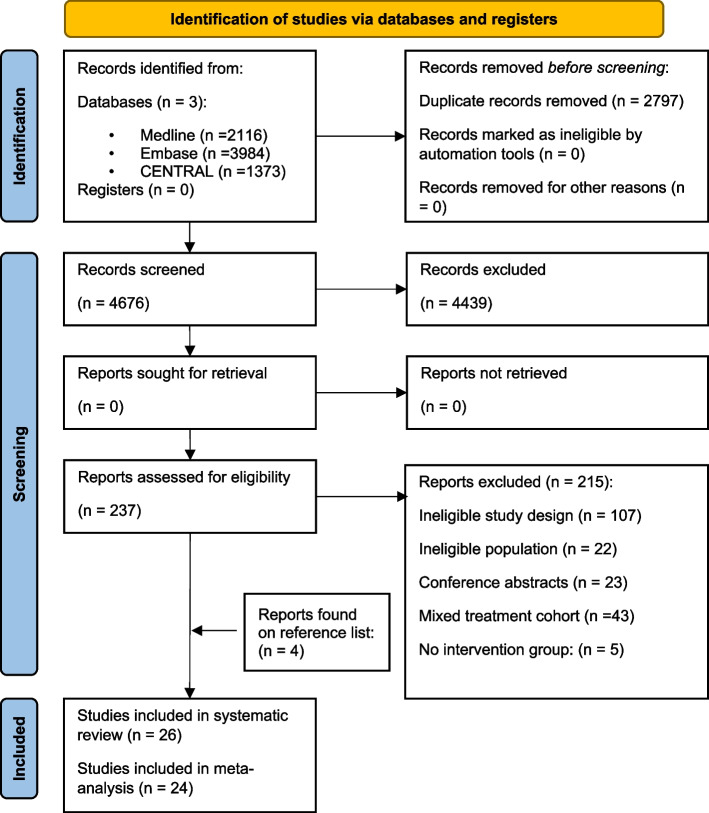
PRISMA 2020 flowchart representing the study selection process

Of 4676 records, 26 RCTs (Fig. 1) were included with 1691 women with PCOS. Twenty-four studies were included in the quantitative synthesis [12–16, 31–49], but two studies were excluded from the meta-analysis due to inappropriate data reporting [50, 51].

### Basic characteristics of the included studies

Baseline characteristics of the included analyses are detailed in Table 1. Most studies included women in their 30 s, with a mean BMI below 30 kg/m2. In the case of two studies, BMI was also an inclusion criterion, meaning they investigated overweight and obese women with PCOS [43, 51]. In Table S2, the inclusion and exclusion criteria of the included studies were summarized. Table S3 summarizes the details of the intervention and control treatment in each study. Eligible studies used either myoinositol or D-chiro-inositol as the investigated intervention. However, the dose and length of administration were different between the studies. One trial compared myoinositol and inositol combinations to diet [15]. A single three-arm trial was included comparing myoinositol to metformin and placebo [44].

**Table 1:** Basic characteristics of the included studies

Study (year)	Country	Study period	Population (I/C)^a^	Age^b^	BMI^b^	Intervention	Control	Outcomes
Angik, 2015 [46]	India	09.2012–08.2014	50/50	NR	23.7	MI 1000 mg 24w	MET 1000 mg	cycle norm., BMI, pregnancy rate, FPI, FPG, HOMA, TT, m-FG score, side effect
Benelli, 2016 [31]	Italy	NR	21/25	24.1	31.5	MI (1100 mg) + DCI (27,6 mg) 24w	FA 400mcg	BMI, FPI, FPG, HOMA index, FT, SHBG, DHEAS, A, side effect
Brusco, 2013 [32]	Italy	06.2012–05.2013	58/91	NR	NR	MI (2000 mg) + DCI (400 mg) 12w	FA 400mcg	pregnancy rate
Chirania, 2017 [47]	India	08.2015–07.2016	26/28	23.8	25.1	MI 1000 mg 16w	MET 1000 mg	cycle. norm., BMI, pregnancy rate, FPI
Chhabra, 2018 [33]	India	NR	31/32	29.7	NR	MI 4000 mg 12w	MET 1700 mg	cycle norm., m-FG score, acne
Costantino, 2009 [34]	Italy and France	NR	23/19	28.3	22.7	MI 4000 mg 12-16w	FA 400 mcg	BMI, FPI, FPG, AUC-glu, AUC-ins, TT, FT, SHBG, DHEAS, A
Doná, 2012 [35]	Italy	NR	18/8	23.5	21.7	MI 1200 mg 12w	Placebo powder	BMI, FPI, FPG, AUC-ins, AUC-glu, HOMA, TT, A
Donne, 2019 [15]	Italy	11.2015–06.2016	22/21	26.7	32	1. MI 4000 mg 24w	diet	cycle norm., BMI, FG-score
						2. MI 1100 mg + DCI 27,6 mg 24w		
Fruzetti, 2016 [36]	Italy	2014–2015	24/22	21.9	27.8	MI 4000 mg 24w	MET 1500 mg	BMI, HOMA, AUC-ins, A, hirsutism, acne
Genazzani, 2008 [37]	Italy	NR	10/10	NR	28.4	MI 2000 mg 12w	FA 200mcg	BMI, FPI, HOMA, glu/ins ratio, TT, A, FG-score
Gerli, 2007 [38]	Italy	NR	45/47	29.4	34.4	MI 4000 mg 14w	FA 400mcg	BMI, pregnancy rate, FPI, FPG, AUC-ins,
H. Jamiliam, 2017 [40]	Iran	06.2016–12.2016	30/30	28.1	27.9	MI 4000 mg 12w	MET 1500 mg	BMI
Iuorno, 2002 [39]	Venezuela	NR	10/10	27.4	24.5	DCI 600 mg 7w	NR	BMI, FPI, FPG, AUC-glu, AUC-ins, TT, FT, SHBG, DHEAS, A, side effect
Leo, 2013^c^ [50]	Italy	NR	20/20	NR	27,5	MI 3000 mg 24w	MET 1700 mg	BMI, FPI, FPG, HOMA, TT, FT, SHBG, A, FG-score
M. Jamiliam, 2017 [41]	Iran	11.2016–02.2017	30/30	26.8	26.5	MI 4000 mg 12w	MET 1500 mg	BMI, TT, SHBG, mFG-score
Nehra, 2017 [42]	India	NR	30/30	23.5	26.3	MI 2000 mg 24w	MET 1500 mg	BMI
Nehra J., 2017 [16]	India	NR	30/30	23.5	26.3	MI 2000 mg 24w	MET 1500 mg	FPI, FPG, Glu/ins ratio, HOMA, TT
Nestler, 1999 [43]	Venezuela	NR	22/22	27.5	31.2	DCI 1200 mg 7w	Placebo	BMI, AUC-glu, AUC-ins, TT, FT, SHBG, DHEAS, A, side effect, presence of ovulation
Pourghasem, 2018 [44]	Iran	2015–2016	50/50/50	30.9	28.3	MI 4000 mg 12w	1.MET 1500 mg	cycle norm., pregnancy rate, side effect
							2.FA 400mcg	
Raffone, 2010 [49]	Italy	06.2006–06.2008	60/60	29.4	25	MI 4000 mg 24w	MET 1500 mg	cycle norm., pregnancy rate
Rajasekaran, 2021 [13]	India	05.2018–03.2020	50/50	30.5	26.5	MI 4000 mg 12w	MET 1700 mg	cycle norm., BMI, pregnancy rate, FPI, FPG, HOMA, TT, SHBG, side effect
Schihalli, 2012 [45]	Italy	01.2010–09.2010	9/8	30.6	NR	MI 4000 mg NR w	FA 400mcg	pregnancy rate
Shokrpour, 2021 [14]	Iran	09.2017–12.2017	26/27	28	27.7	MI 4000 mg 12w	MET 1500 mg	BMI, FPG, Insulin, HOMA
Singh, 2020 [48]	India	04.2013–08.2014	66/66	NR	31.8	MI 4000 mg 12w	FA 500mcg	BMI, FPI, FPG, TT
Soldat-Stankovic, 2021 [12]	Bosnia-Herzegovina	11.2017–05.2019	30/30	NR	26.1	MI 4000 mg 24w	MET 1500 mg	BMI, FPI, FPG, AUC-glu, AUC-ins, HOMA, TT, SHBG, DHEAS, FG-score,side effect
Tagliaferri, 2017^c^ [51]	Italy	NR	14/20	25.6	32.6	MI 1000 mg 24w	MET 1700 mg	BMI, pregnancy rate, AUC-ins, AUC-glu, TT, SHBG, DHEAS, A, FG-score, side effect,

NR: not reported. Cycle norm.: cycle normalization; TT: total testosterone; FT: free testosterone; SHBG: sex-hormone binding globulin; A: androstenedione; DHEAS: dehydroepiandrosteron- sulfate; FG-score: Ferriman-Gallwey score; mFG-score: modified Ferriman- Gallwey score; AUC-Glu: Area under the curve- glucose; AUC-ins: Area under the curve – insulin; FPG: fasting plasma glucose; FPI: fasting plasma insulin; Glu/ins ratio: glucose / insulin ratio

^a^I/C intervention/ control

^b^Age (years) and BMI (kg/m2) are expressed in mean

^c^studies included only in the systematic review part

### Inositol treatment promotes ovarian cycle normalization and contributes to weight loss

**Fig. 2:**
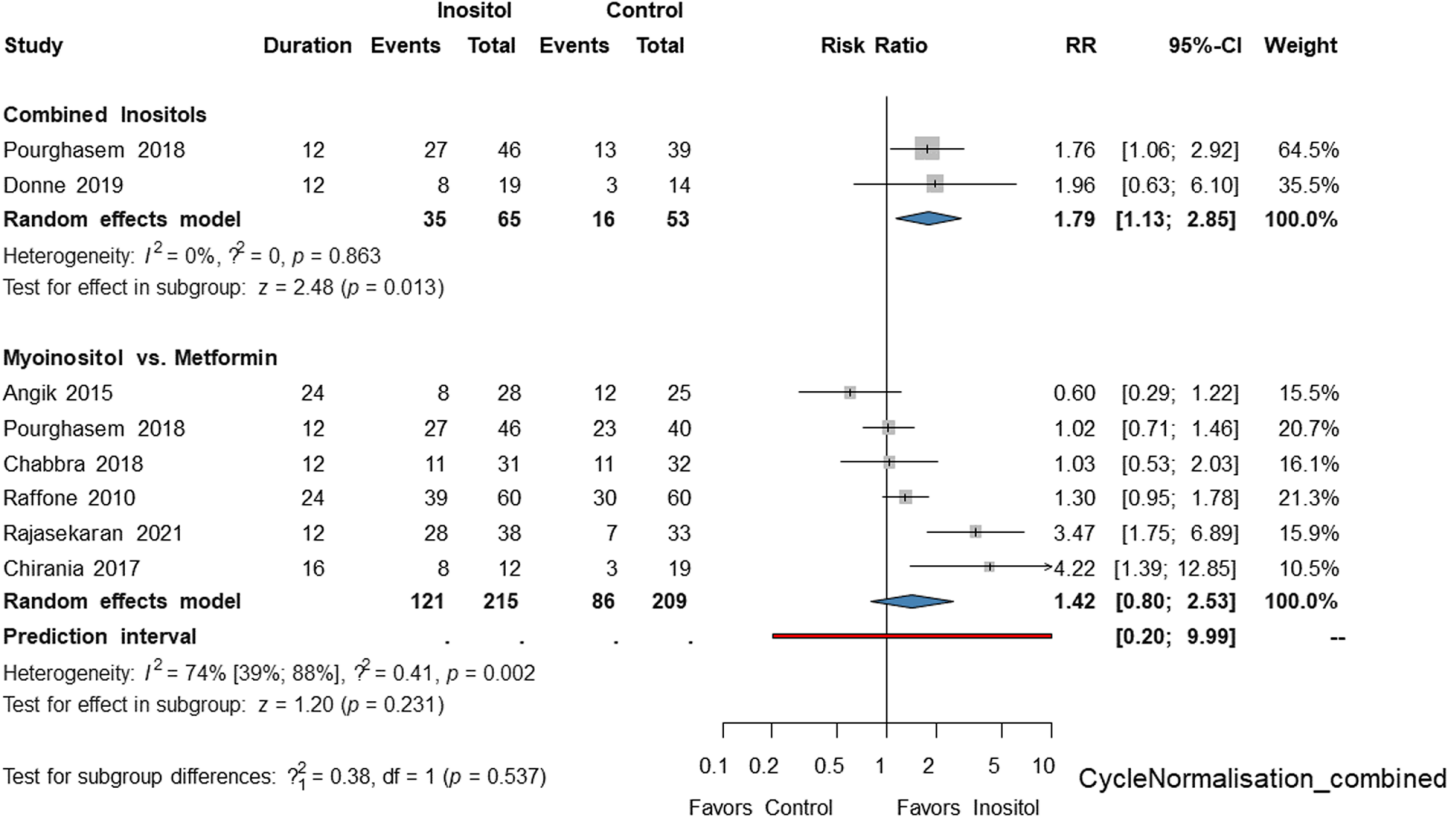
Forest plots representing the risk of cycle normalization in the groups treated with inositols compared to placebo or metformin

Results of the pooled analysis are included in Table 2 and 3. On the basis of two eligible studies, the rate of cycle normalization was higher in the inositol group compared to the placebo (RR = 1.79, CI: 1.13; 2.85, Fig. 2).

**Table 2:** Summary of studies comparing inositol stereoisomers to placebo

Outcomes	Inositol vs Placebo			Myo-Inositol vs Placebo			DCI vs Placebo			Inositol combination vs Placebo		
	**N**^**0**^** of studies** **(N**^**0**^** of pts)**	**RR/ MD** **(95% CI)**	**GRADE**	**N**^**0**^** of studies** **(N**^**0**^** of pts)**	**RR/ MD** **(95% CI)**	**GRADE**	**N**^**0**^** of studies** **(N**^**0**^** of pts)**	**RR/ MD** **(95% CI)**	**GRADE**	**N**^**0**^** of studies** **(N**^**0**^** of pts)**	**RR/ MD** **(95% CI)**	**GRADE**
Total testosterone (ng/dl)	**6 (284)**	**-20.39 (-40.12; -0.66)**	**moderate**	4 (220)	-11.38 (-29.48; 6.72)	moderate	**2 (64)**	**-41,71 (-70.09; -13.34)**	**high**	-	-	-
Free testosterone (ng/dl)	**4 (152)**	**-0.41 (-0.69; -0.13)**	**moderate**	1 (42)	-0.57 (-1; -0.14)	high	**2 (64)**	**-0.58 (-0.89; -0.28)**	**high**	1 (46)	-0,12 (-0.28; 0.04)	low
SHBG (nmol/L)	**4(152)**	**32.06 (1.27; 62.85)**	**moderate**	1 (42)	37.6 (-43.97; 119.17)	moderate	**2(64)**	**55.45 (25.99; 84.91)**	**high**	1 (46)	10.82 (-1.7; 23.34)	moderate
Androstenedione (ng/ml)	**6 (198)**	**-0.69 (-1.16; -0.22)**	**moderate**	**3 (88)**	**-0.89 (-1.56; -0.22)**	**very low**	**2 (64)**	**-0.52 (-1.13; -0.09)**	**low**	1 (46)	0.12 (-1.3; 1.54)	low
DHEAS (µg/dl)	4 (152)	-92.54 (-206.31; 21.22)	low	1 (42)	-114 (-294.53; 66.53)	high	**2 (64)**	**-168,48 (-281.15; -55.82)**	**moderate**	1 (46)	42.4 (-89.68; 174.48)	low
Ferriman-Gallwey score	1(43)	-1.23 (-5.37; 2.92)	low	-	-	-	-	-	-	1 (43)^a^	-1.23 (-5.37; 2.92^a^	low^a^
Glucose (mg/dl)	**5 (266)**	**-3.14 (-5.75; -0.54)**	**moderate**	**3 (200)**	**-4.03(-6.59; -1.47)**	**moderate**	1 (20)	-9 (-28.26; 10.26)	low	1 (46)	2.47 (-3.96; 8.9)	low
Insulin (µU/ml)	6 (286)	-2.78 (-7.13; 1.57)	low	4 (220)	0.46 (-0.68; 1.59)	low	1 (20)	-13 (-41,03; 15.03)	low	1 (46)	-9.25 (-15.57; -2.93)	high
HOMA index	3 (92)	-0.23 (-1.19; 0.72)	low	2 (46)	-0.17 (-1.57; 1.23)	moderate	-	-	-	1 (46)	-0.73 (-2.2; 0.74)	low
AUC Glucose (mg/dl/min)	4 (132)	-763.3 (-1925.05; 398.45)	moderate	2 (68)	-550.98 (-2182.91; 1080.95)	low	2 (64)	-1502.41 (-3406.31; 401.48)	low	-	-	-
AUC insulin (µU/ml/min)	**4 (132)**	**-2081.05 (-2745.32; -1,416.78)**	**high**	**2 (68)**	**-2034.05 (-2706.3; -1361.81)**	**high**	2 (64)	-4027.17 (-8352.7; 298.33)	moderate	-	-	-
BMI (kg/m2)	**8 (419)**	**-0.45 (-0.89; -0.02)**	**high**	**5 (312)**	**-0.71 (-1.00; -0.43)**	**high**	2 (64)	0.35 (-0.56; 1.27)	low	1 (43)^a^	-0.21( -4.44; 4.01)^a^	low^a^
cycle normalization	**2 (118)**	**1.79 (1.13; 2.85)**	**very low**	1(85)	1.76 (1.06; 2.92)	moderate	-	-	-	1 (33)^a^	1.96 (0.63; 6.1)a	low^a^
pregnancy rate	4 (308)	1.24 (0.85; 1.81)	very low	3 (159)	0.92 (0.53; 1.61)	very low	-	-	-	1 (149)	1.45 (1.06; 1.98)	low
pregnancy rate (no other treatment)	1 (42)	3.3 (0.4; 27.13)	low	1 (42)	3.3 (0.4; 27.13)	very low	-	-	-	-	–	-

Significant results are written with bold numbers

^a^means MYO and MYO + DCI treated group is pooled into one group [15]

**Table 3:** Summary of studies comparing myoinositol treatment to metformin

Outcomes	Inositol vs Metformin		
	**N**^**0**^** of studies** **(N**^**0**^** of pts)**	**RR/ MD** **(95% CI)**	**GRADE**
Total testosterone (ng/dl)	4 (320)	0.2 (-5.72; 6.12)	moderate
Free testosterone(ng/dl)	-	-	-
**SHBG (nmol/L)**	**3 (220)**	**2.78 (0.02; 5.54)**	**moderate**
Androstenedione (ng/ml)	-	-	-
DHEAS (µg/dl)	1 (60)	17.31 (-17.84; 52.46)	low
**Ferriman-Gallwey score**	**3 (220)**	**0.6 (0.24; 0.96)**	**high**
Glucose (mg/dl)	5 (373)	-0.84 (-3.62; 1.93)	low
Insulin (µU/ml)	6 (427)	-0.37 (-1.52; 0.78)	high
HOMA index	6 (419)	-0.18( -0.41; 0.06)	high
AUC Glucose (mg/dl/min)	1 (60)	1218.76 (-812.79; 3250.3)	moderate
AUC insulin (µU/ml/min)	1 (60)	1593.71 (-2802.06; 5989.5)	moderate
BMI (kg/m2)	9 (593)	-0,11 (-0.25; 0.04)	high
cycle normalisation	6 (424)	1.42 (0.8; 2.53)	very low
pregnancy rate	5 (383)	1.22 (0.84; 1.78)	very low
pregnancy rate (no other treatment)	3 (183)	1.38 (0.88; 2.15)	very low

Significant results are written with bold numbers

The pooled analysis of eight RCTs showed a higher reduction in BMI in the inositol group compared to placebo (MD = -0.45 kg/m^2^, CI: -0.89; -0.02, Figure S2a). Particularly, myoinositol seems to have a beneficial effect on weight loss MD = -0.71 kg/m^2^ (CI: -1.00; -0.43 kg/m^2^, Figure S2b.).

Myoinositol had an efficacy similar to metformin regarding cycle normalization (RR = : 1.42 CI: 0.8; 2.53, Fig. 2) and BMI reduction (MD = -0,11 kg/m2, CI: -0.25; 0.04, Figure S2c.).

### Androgens in PCOS

Compared to placebo, inositols significantly reduced total testosterone levels (MD = -20.39 ng/dl, CI: -40.12; -0.66, Figure S3a.). Two studies showed an advantageous effect of DCI for this outcome. On the other hand, free testosterone was significantly reduced by inositol treatment compared to placebo (MD = -0.41 ng/dl, CI: -0.69; -0.13, Figure S4.). SHBG levels were significantly increased by inositols (MD = 32.06 nmol/l, CI: 1.27; 62.85, Figure S5a.). Androstenedione was also significantly reduced after inositol treatment (MD = -0.69 ng/ml, CI: -1.16; -0.22, Figure S6.). Myoinositol, compared to placebo, also seems to have a beneficial effect on androstenedione (MD = 0.89 ng/ml, CI: -1.56; -0.22, Figure S6.). DCI reduced DHEAS levels (MD = -168.48 μg/dl, CI-281.15; -55.82, Figure S7a.). However, the combined analysis of different inositols did not reach the level of significance. Finally, only one study investigated the effect of inositol on the FG-score [15].

Compared to metformin, myoinositol significantly increased SHBG levels (MD = 2.78 nmol/l, CI: 0.02; 5.54, Figure S5a.). However, metformin seemed more effective in decreasing FG-score (MD = 0.6, CI: 0.24; 0.96, Figure S8) than inositol. In the case of total testosterone levels, inositol was non-inferior compared to metformin (Figure S3b.). However, only one RCT reported on DHEAS [12], and no articles compared inositol to metformin regarding free testosterone and androstenedione levels.

### Glucose metabolism in PCOS

Inositols significantly reduced fasting plasma glucose compared to placebo (MD = -3.14 mg/dl, CI: -5.75; -0.54, Figure S9a.). The analysis showed that myoinositol has the most pronounced effect on glucose levels (MD = -4.03 mg/dl, CI: -6.59; -1.47, Figure S9a.). In the case of fasting plasma insulin, HOMA-IR, and AUC-glucose, inositols compared to placebo showed no favorable effect (Figure S10-12a.). In general, inositols significantly reduced AUC-insulin levels (MD = -2081.05 μU/ml/min, CI: -2745.32; -1416.78, Figure S13a.). However, according to the subgroup analysis, myoinositol seems to benefit AUC-insulin levels compared to placebo (MD = -2034.05 μU/ml/min, CI: -2706.3; -1361.81, Figure S13a.).

No significant differences were found between the inositol and the metformin treatment regarding the investigated glycemic outcomes, suggesting non-inferiority of inositols to metformin (Figure S9-13b.).

### Pregnancy in PCOS

The pregnancy rate was reported in eight RCTs, while in four articles, the inositol therapy was followed by additional therapy such as letrozole or a combination of rFSH and HCG injection. The overall pregnancy outcome was heterogenous regarding its definition, carrying a significant risk of bias.

Only one study reported on the pregnancy rate for the inositol placebo comparison without additional therapy and found no difference (RR = 3.3 CI: 0.4; 27.13, Figure S14.) [38]. Similarly, the pool of studies with inositol therapy, followed by additional therapy, showed no significant difference in the pregnancy rate compared to placebo (RR = 1.24, CI: 0.85; 1.81, Figure S15a.).

When compared to metformin, inositols showed similar results with (RR = 1.22, CI: 0.84; 1.78, Figure S15b.) and without (RR = 1.38, CI: 0.88; 2.15, Figure S14.) additional therapy.

### Side effects

Four studies comparing inositol to placebo reported no side effects for inositols. Furthermore, four studies comparing inositol to metformin showed a lower rate of side effects in the inositol group (7 vs. 53%, RR = 0.16, CI: 0.09; 0.28, Figure S16.). Side effects in the metformin group were bloating, nausea, and generalized weakness.

### Risk of bias assessment, quality of evidence

The summary of the RoB 2 risk of bias assessment can be found in Table S4. Furthermore, the level of evidence is summarized in Tables 2 and 3 and Tables S5-9. For most of the outcomes the level of evidence was moderate.

## Discussion

According to the present meta-analysis, inositols have a beneficial effect on all aspects of PCOS. First, inositols reduce serum total and free testosterone and androstenedione levels, increase SHBG levels, and normalize cycle length compared to placebo. On the other hand, in all these parameters, they were not inferior to metformin. Furthermore, a significant decrease was found in fasting glucose and AUC insulin levels and BMI in the inositol-treated groups. Of the analyzed isomers, myoinositol has the most supported benefit. Finally, compared to metformin, inositols showed fewer side effects.

Myoinositol is synthesized from glucose-6-phosphate (G6P) endogenously. On the other hand, it can be found in the cell membranes as phosphatidyl-myoinositol as the precursor of inositol triphosphate (PIP2), which plays a crucial role [52] in the signal transduction of various receptors, including FSH, promoting granulosa cell differentiation and follicle maturation [47]. In addition, myoinositol might improve oocyte and embryo quality [5] by encouraging translocation of GLUT4 to the plasma membrane in order to increase glucose uptake [53] and promote aromatase activity. During the secondary signaling mechanisms, inositol triphosphate (IP3) will also be released, which can be converted to free myoinositol by inositol-monophosphatase [6].

Data on cycle regularization was heterogeneous. Menstrual cycle regularization was considered if the patient had amenorrhea or oligomenorrhea, and after the treatment, they had eumenorrhea. The results of Genazzani et al. were not included in the analysis as they reported improvement if the patient became oligomenorrheic from amenorrhea [37] Pundir et al. reported similar results [19]. However, in their included studies, cycle normalization was heterogenous, and some of them could not be included in the pool.

In the case of DHEAS and androstenedione, no significant difference was found. Results are mostly consistent with those of Zeng et al., but other inositol stereoisomers were also investigated in the present study [54] They found no statistical difference between myoinositol and the placebo group regarding total testosterone levels. However, on the basis of two articles, they found a decrease in free testosterone. In comparison with Pundir et al., one more RCT was included in the present analysis [31], and no difference was found in DHEAS levels after inositol treatment compared to placebo [19, 31]. On the other hand, Kutenai et al. reported that myoinositol reduced total testosterone and DHEA level more effectively than metformin [55]. These results might be the consequence of the more effective aromatase activity.

According to our analysis, inositols increase the concentration of SHBG, mainly due to their effect on insulin resistance. Moreover, as precursors of inositol triphosphate (PIP2), they play a crucial role in insulin signal transduction. Inositols have a dual effect on free androgen concentration: (1) through their contribution to follicle maturation, they can improve the mechanism of dominant follicle selection, increasing aromatase activity, and thus effectively reducing total androgen production, (2) they also induce the production of SHBG, leading to a reduction in free androgen levels. Inositols seem to reduce testosterone and androstenedione levels but not DHEA concentrations, suggesting that their antiandrogen effect is mainly based on the improvement of ovarian function. On the other hand, DCI is an aromatase inhibitor and promotes glycogen synthase, which inhibits the conversion of androgens to estrogens, resulting in the accumulation of androgens and lack of estrogens. That is why long-term or high-dose DCI administration will worsen the symptoms of PCOS. However, in the short term, it can improve insulin levels, thus promoting SHBG production [6]. According to our data, 6–8-week treatment had no adverse effects on androgen levels.

Zeng et al. also analyzed the effect of inositols on SHBG. However, they only included two articles and showed that myoinositol might have a better effect on SHBG compared to placebo [54].

According to our data, inositols are non-inferior to metformin regarding its effect on free and total testosterone, androstenedione, and SHBG. In accordance with our results, Zhang et al. found similar improvement in total testosterone, SHBG, BMI, fasting insulin (FI), and fasting blood glucose (FBG), while Fanchinetti et al. in testosterone, androstenedione, and SHBG levels after inositol treatment compared to metformin [3, 56].

After inositol treatment, an improvement was found in hyperinsulinemia and carbohydrate metabolism compared to placebo. Our data show similar results to previously published meta-analyses [19, 54]. However, the included studies partially overlap with those in our analysis. On the other hand, Zeng et al. failed to show a beneficial effect of inositols on fasting glucose compared to placebo [54]. Compared to metformin, inositol also seemed non-inferior regarding carbohydrate metabolism. Zhang et al. and Kutanei et al. showed similar results to the one in the present meta-analysis. However, Fanchinetti et al. showed no significant difference in the efficacy of myoinositol and metformin regarding fasting insulin and HOMA index [56].

Contrary to our results, Zeng et al. meta-analysis’ reported that myoinositol had no beneficial effect on weight loss [54]. However, they only pooled the after-treatment BMI values and not the change. On the other hand, inositols were noninferior to metformin regarding BMI decrease, which is similar to previous meta-analyses published by Fanchinetti et al. and Zhang et al. [3, 56].

### Strengths and limitations

The strength of the study was that a strict protocol was followed. Inositols were compared not only to placebo, but also to the gold standard treatment of metformin. Different stereoisomers were also investigated separately to examine which is the most effective in PCOS. No language restrictions were used. Finally, a rigorous methodology was applied.

The limitations of this analysis were the small number of studies with small sample size, and the heterogeneous populations. Furthermore, the follow-up time differed among the studies. In addition, studies investigated different dosages of inositols in inositol monotherapy compared to inositol combinations. The generalization of pregnancy rate results was problematic since only one study analyzed women who wanted pregnancy. In addition, graphs of AUC-insulin and glucose were missing. Therefore, interpreting these results was complicated to judge the effect of inositols and metformin on early and late insulin responses. Lastly, the presence of moderate and high risk of bias in some domains was another limitation.

### Implications for practice and research

Inositols should be included in the treatment protocol of PCOS, especially in women suffering from side effects of metformin. Further well-designed RCTs are needed to assess the beneficial effect on pregnancy rate. Investigators should also consider examining the effect of metformin and inositol co-treatment.

## Conclusion

On the basis of our results, inositols have a beneficial effect on several outcomes of PCOS. Moreover, inositols showed non-inferiority in almost all outcomes compared to metformin, representing a promising alternative treatment in PCOS. Therefore, it is recommended that inositols be included in the guideline for the treatment of PCOS.

## Supplementary Information

**Additional file 1.** Supplementary material - Inositol is an effective and safe treatment in polycystic ovary syndrome: a systematic review and meta-analysis with randomized controlled trials. The present study investigated several outcomes , which resulted more than 40 forest plots. The results were summarized in Tables 2 and 3 in the main text, and forest plots are presented in the supplementary material.

## Data Availability

The datasets used in this study can be found in the full-text articles included in the systematic review and meta-analysis.

## Abbreviations

AUC: Area under the curve
BMI: Body mass index
CI: Confidence interval
DCI: D-chiro-inositol
DHEAS: Dehydroepiandrosterone-sulfate
FBS: Fasting blood glucose
FI: Fasting insulin
FSH: Follicle-stimulating hormone
G6P: Glucose-6-phosphate
GLUT4: Glucose transporter type 4
Figure S: Figure in Supplementary material
GRADE: Grades of Recommendation, Assessment, Development, and Evaluation
HOMA-IR: Homeostatic Model Assessment insulin resistance
IP3: Inositol triphosphate
IR: Insulin resistance
MD: Mean difference
OGTT: Oral glucose tolerance test
PCOS: Polycystic ovary syndrome
PICO: Population, Intervention, Comparator, Outcome
PIP2: Inositol triphosphate
RCT: Randomized controlled trial
RoB 2: Risk-of-bias tool for randomized trials
RR: Risk ratio
SD: Standard deviation
SHBG: Sex hormone binding globulin
