# Supplementary material for: Inositol is an effective and safe treatment in polycystic ovary syndrome: a systematic review and meta-analysis of randomized controlled trials
Source: Reprod Biol Endocrinol. 2023 Jan 26;21:10. doi: 10.1186/s12958-023-01055-z (PMC9878965; doi:10.1186/s12958-023-01055-z)
Supplement: Supplementary file 1 — Additional file 1. Supplementary material - Inositol is an effective and safe treatment in polycystic ovary syndrome: a systematic review and meta-analysis with randomized controlled trials. The present study investigated several outcomes , which resulted more than 40 forest plots. The results were summarized in Tables 2 and 3 in the main text, and forest plots are presented in the supplementary material. [file 12958_2023_1055_MOESM1_ESM.docx]

**SUPPLEMENTARY MATERIAL**

**Title**

Inositol is an effective and safe treatment in polycystic ovary syndrome: a systematic review and meta-analysis with randomized controlled trials

**Authors**

Dorina Greff^1,2,3^, Anna E. Juhász^1,4^, Szilárd Váncsa^1,6,7^, Alex Váradi^6^, Zoltán Sipos^6^, Julia Szinte^1,2,3^, Sunjune Park^1,3^, Péter Hegyi^1,6,7^, Péter Nyirády^8^, Nándor Ács^2^, Szabolcs Várbíró^2,5^, Eszter M. Horváth^3^

**Affiliations:**

^1^Centre for Translational Medicine, Semmelweis University, Budapest, Hungary

^2^Department of Obstetrics and Gynecology, Semmelweis University, Budapest, Hungary

^3^Department of Physiology, Semmelweis University, Budapest, Hungary

^4^Department of Dietetics and Nutrition Sciences, Semmelweis University, Budapest, Hungary

^5^Workgroup for Science Management, Doctoral School, Semmelweis University, Budapest,

Hungary

^6^Institute for Translational Medicine, Szentágothai Research Centre, Medical School, University of Pécs, Pécs, Hungary

^7^ Institute of Pancreatic Diseases, Semmelweis University, Budapest, Hungary

^8^Department of Urology, Semmelweis University, Budapest, Hungary

**TABLE OF CONTENT**

**Figure S1.** Forest plots representing the risk of cycle normalization in the groups treated with inositols compared to placebo or metformin.

**Figure S2a**. Forest plots representing the mean difference of weight loss in the groups treated with inositols compared to placebo.

**Figure S2b.** Forest plots representing the mean difference of weight loss in the groups treated with different inositol stereoisomers compared to placebo.

**Figure S2c.** Forest plots representing the mean difference of weight loss in the groups treated with inositols compared to metformin.

**Figure S2d.** Forest plots representing the mean difference of weight loss in the groups treated with inositols compared to placebo or metformin.

**Figure S2e.** Forest plots representing the mean difference of weight loss in the groups treated with inositols compared to placebo or metformin with imputed correlations.

**Figure S3a.** Forest plots representing the mean difference of total testosterone levels in the groups treated with different inositol stereoisomers compared to placebo.

**Figure S3b.** Forest plots representing the mean difference of total testosterone levels in the groups treated with inositols compared to metformin.

**Figure S3c.** Forest plots representing the mean difference of total testosterone levels in the groups treated with inositols compared to placebo or metformin.

**Figure S3d.** Forest plots representing the mean difference of total testosterone levels in the groups treated with inositols compared to placebo or metformin. (Summary of different inositols into one ‘combined inositols’group.)

**Figure S4**. Forest plots representing the mean difference of free testosterone levels in the groups treated with inositols compared to placebo.

**Figure S5a.** Forest plots representing the mean difference of SHBG levels in the groups treated with inositols compared to placebo or metformin. (Summary of different inositols into one ‘combined inositols’group.)

**Figure S5b.** Forest plots representing the mean difference of SHBG levels in the groups treated with inositols compared to placebo or metformin.

**Figure S5c.** Forest plots representing the mean difference of SHBG levels in the groups treated with different inositol stereoismers compared to placebo.

**Figure S6.** Forest plots representing the mean difference of androstenedione levels in the groups treated with inositols compared to placebo.

**Figure S7a.** Forest plots representing the mean difference of DHEAS levels in the groups treated with different inositol stereoisomers compared to placebo.

**Figure S7b.** Forest plots representing the mean difference of DHEAS levels in the groups treated with inositols compared to placebo or metformin.

**Figure S7c.** Forest plots representing the mean difference of DHEAS levels in the groups treated with inositols compared to placebo or metformin. (Summary of different inositols into one ‘combined inositols’group.)

**Figure S8.** Forest plots representing the mean difference of Ferriman–Gallwey score in the groups treated with inositols compared to placebo or metformin.

**Figure S9a.** Forest plots representing the mean difference of fasting plasma glucose in the groups treated with different inositol stereoisomers compared to placebo.

**Figure S9b.** Forest plots representing the mean difference of fasting plasma glucose in the groups treated with inositols compared to metformin.

**Figure S9c.** Forest plots representing the mean difference of fasting plasma glucose in the groups treated with inositols compared to placebo or metformin.

**Figure S9d.** Forest plots representing the mean difference of fasting plasma glucose in the groups treated with inositols compared to placebo or metformin. (Summary of different inositols into one ‘combined inositols’group.)

**Figure S10a.** Forest plots representing the mean difference of fasting plasma insulin in the groups treated with different inositol stereoisomers compared to placebo.

**Figure S10b.** Forest plots representing the mean difference of fasting plasma insulin in the groups treated with inositols compared to metformin.

**Figure S10c.** Forest plots representing the mean difference of fasting plasma insulin in the groups treated with inositols compared to placebo or metformin.

**Figure S10d.** Forest plots representing the mean difference of fasting plasma insulin in the groups treated with inositols compared to placebo or metformin. (Summary of different inositols into one ‘combined inositols’group.)

**Figure S11a.** Forest plots representing the mean difference of HOMA-IR in the groups treated with different inositol stereoisomers compared to placebo.

**Figure S11b.** Forest plots representing the mean difference of HOMA-IR in the groups treated with inositols compared to metformin.

**Figure S11c.** Forest plots representing the mean difference of HOMA-IR in the groups treated with inositols compared to placebo or metformin.

**Figure S11d.** Forest plots representing the mean difference of HOMA-IR in the groups treated with inositols compared to placebo or metformin. (Summary of different inositols into one ‘combined inositols’group).

**Figure S12a.** Forest plots representing the mean difference of AUC Glucose in the groups treated with different inositol stereoisomers compared to placebo.

**Figure S12b.** Forest plots representing the mean difference of AUC Glucose in the groups treated with inositols compared to metformin.

**Figure S12c.** Forest plots representing the mean difference of AUC Glucose in the groups treated with inositols compared to placebo or metformin.

**Figure S13a.** Forest plots representing the mean difference of AUC insulin in the groups treated with different inositol stereoisomers compared to placebo.

**Figure S13b.** Forest plots representing the mean difference of AUC Insulin in the groups treated with inositols compared to metformin.

**Figure S13c.** Forest plots representing the mean difference of AUC Insulin in the groups treated with inositols compared to placebo or metformin.

**Figure S13d.** Forest plots representing the mean difference of AUC Insulin in the groups treated with inositols compared to placebo or metformin. (Summary of different inositols into one ‘combined inositols’group).

**Figure S14.** Forest plots representing the risk of pregnancy in the groups treated with inositols compared to placebo or metformin (without any other additional treatment).

**Figure S15a.** Forest plots representing the risk of pregnancy in the groups treated with different inositol stereoisomers compared to placebo.

**Figure S15b.** Forest plots representing the risk of pregnancy in the groups treated with inositols compared to metformin.

**Figure S15c.** Forest plots representing the risk of pregnancy in the groups treated with inositols compared to placebo or metformin.

**Figure S15d.** Forest plots representing the risk of pregnancy in the groups treated with inositols compared to placebo or metformin. (Summary of different inositols into one ‘combined inositols’ group).

**Figure S16.** Forest plots representing the risk of side effect in the groups treated with inositols compared to placebo or metformin.

**Figure S17.** Visualisation of ROB2. Comparison of inositol treatment to placebo.

**Figure S18.** Visualisation of ROB2. Comparison of inositol treatment to metformin.

**Table S1.** PRISMA checklist

**Table S2.** Eligibility criteria of the population of the included studies

**Table S3.** The list of intervention and control in the groups

**Table S4.** Risk of bias assessment using the Risk of Bias 2 tool

**Table S5.** GRADE: The quality of evidence in the inositol treated groups compared to placebo

**Table S6.** GRADE: The quality of evidence in the myoinositol treated group compared to placebo

**Table S7.** GRADE: The quality of evidence in the DCI treated group compared to placebo

**Table S8.** GRADE: The quality of evidence in the myo-inositol and DCI treated group compared to placebo

**Table S9.** GRADE: The quality of evidence in the myo-inositol treated group compared to metformin.

**Supplementary results 1.:** PCOS patomechanism

**Supplementary results 2.:** FG-score

**Supplementary results 3.** Side effects

**Supplementary results 4.:** Pregnancy rate

**Supplementary results 5.:** Different inositol stereoisomers

**Figure S1.** Forest plots representing the risk of cycle normalization in the groups treated with inositols compared to placebo or metformin.


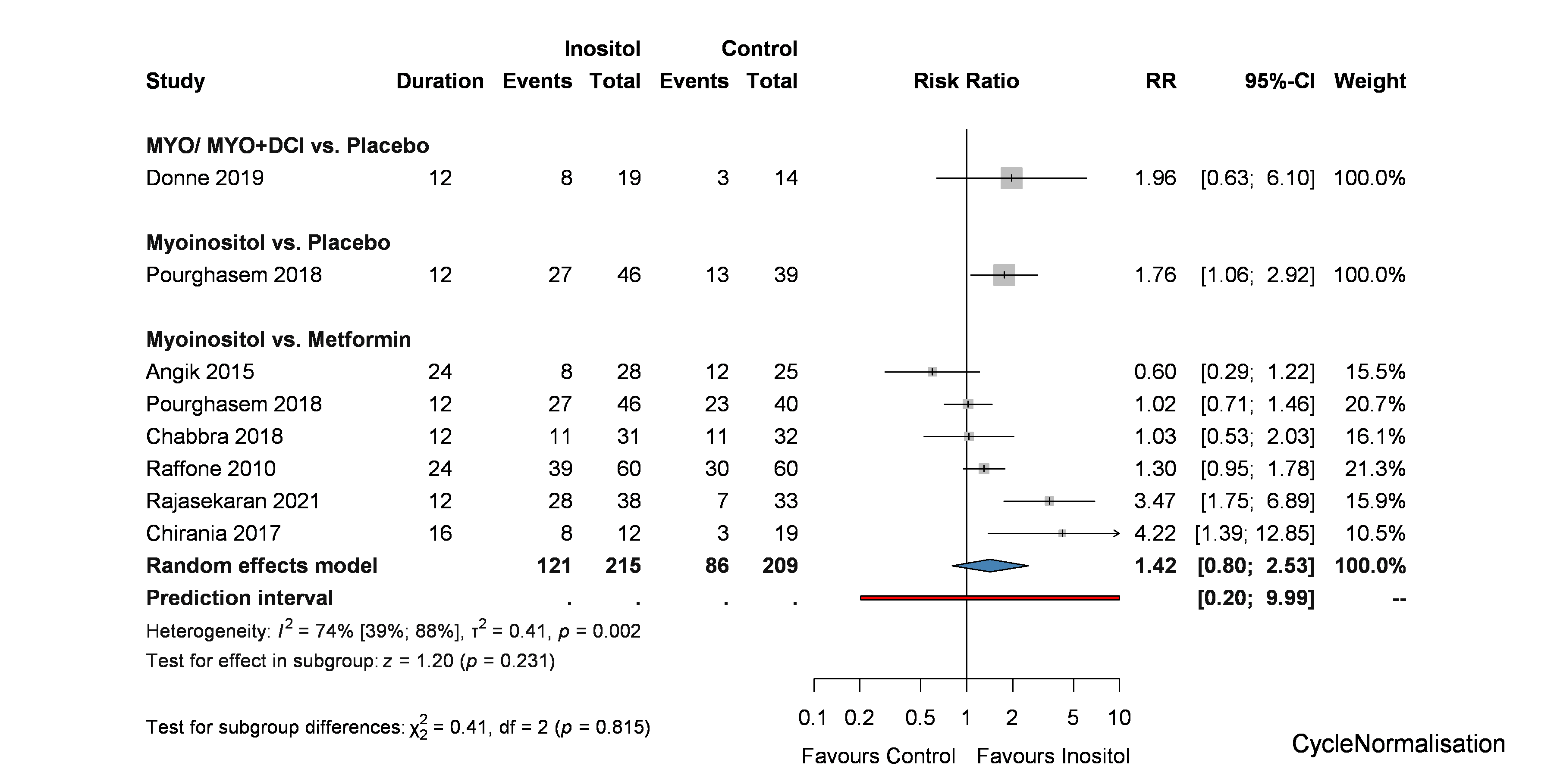


**Figure S2a.** Forest plots representing the mean difference of weight loss in the groups treated with inositols compared to placebo.


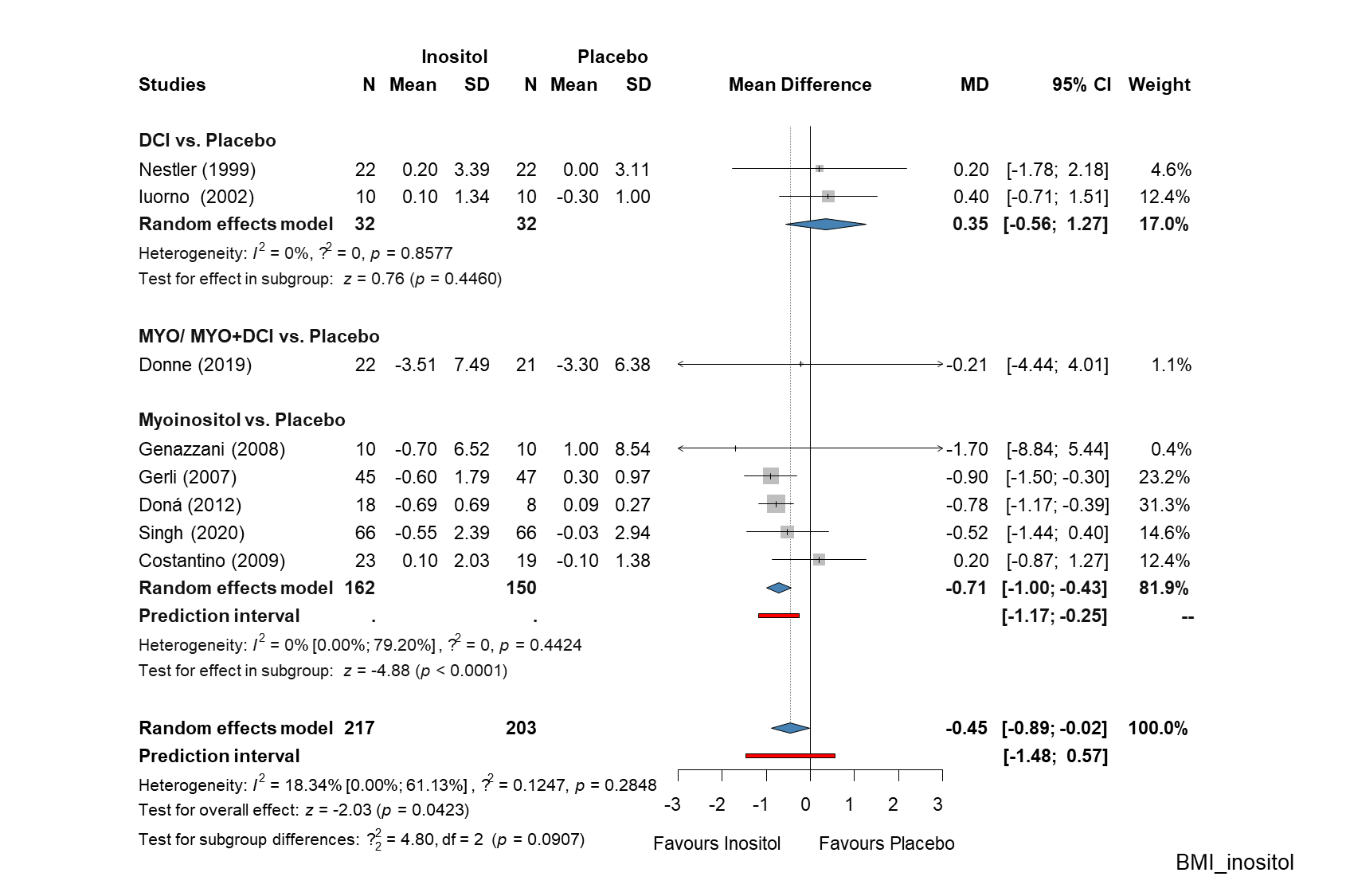


**
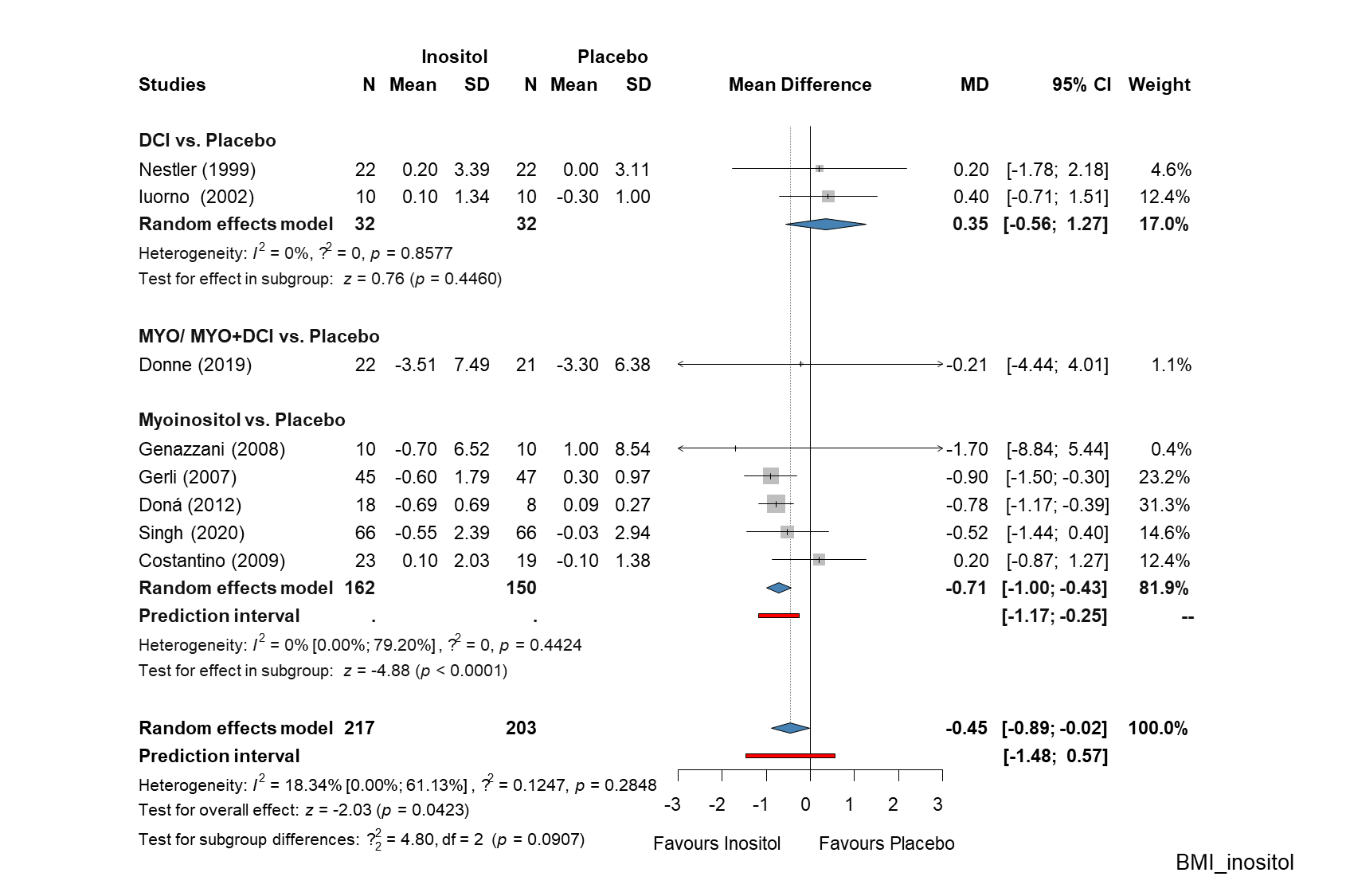
Figure S2b.** Forest plots representing the mean difference of weight loss in the groups treated with different inositol stereoisomers compared to placebo.

**Figure S2c.** Forest plots representing the mean difference of weight loss in the groups treated with inositols compared to metformin.


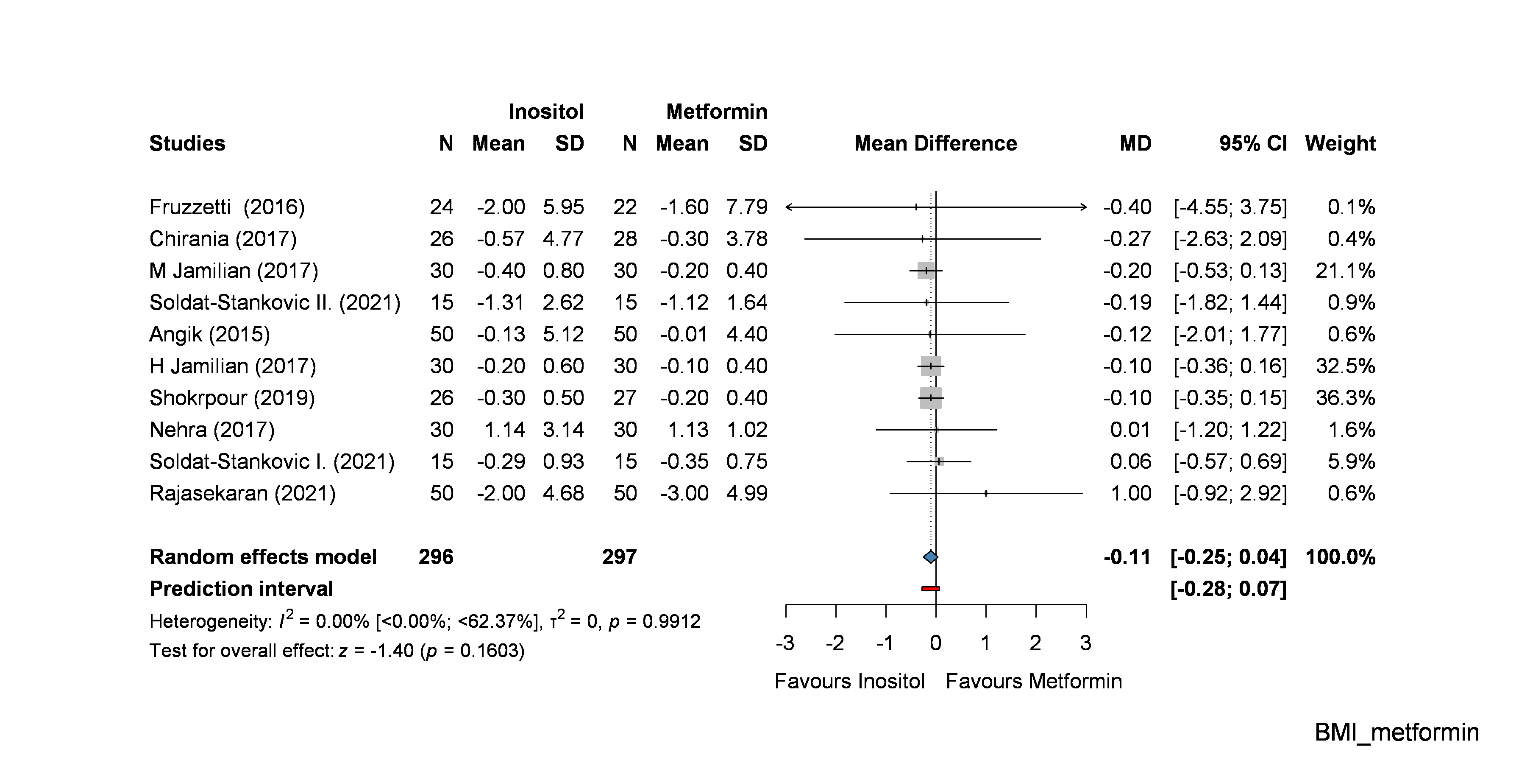


Soldat-Stankovic I. (2021) : BMI < 25kg/m^2^  ; Soldat-Stankovic II. (2021): BMI > 25kg/m^2^


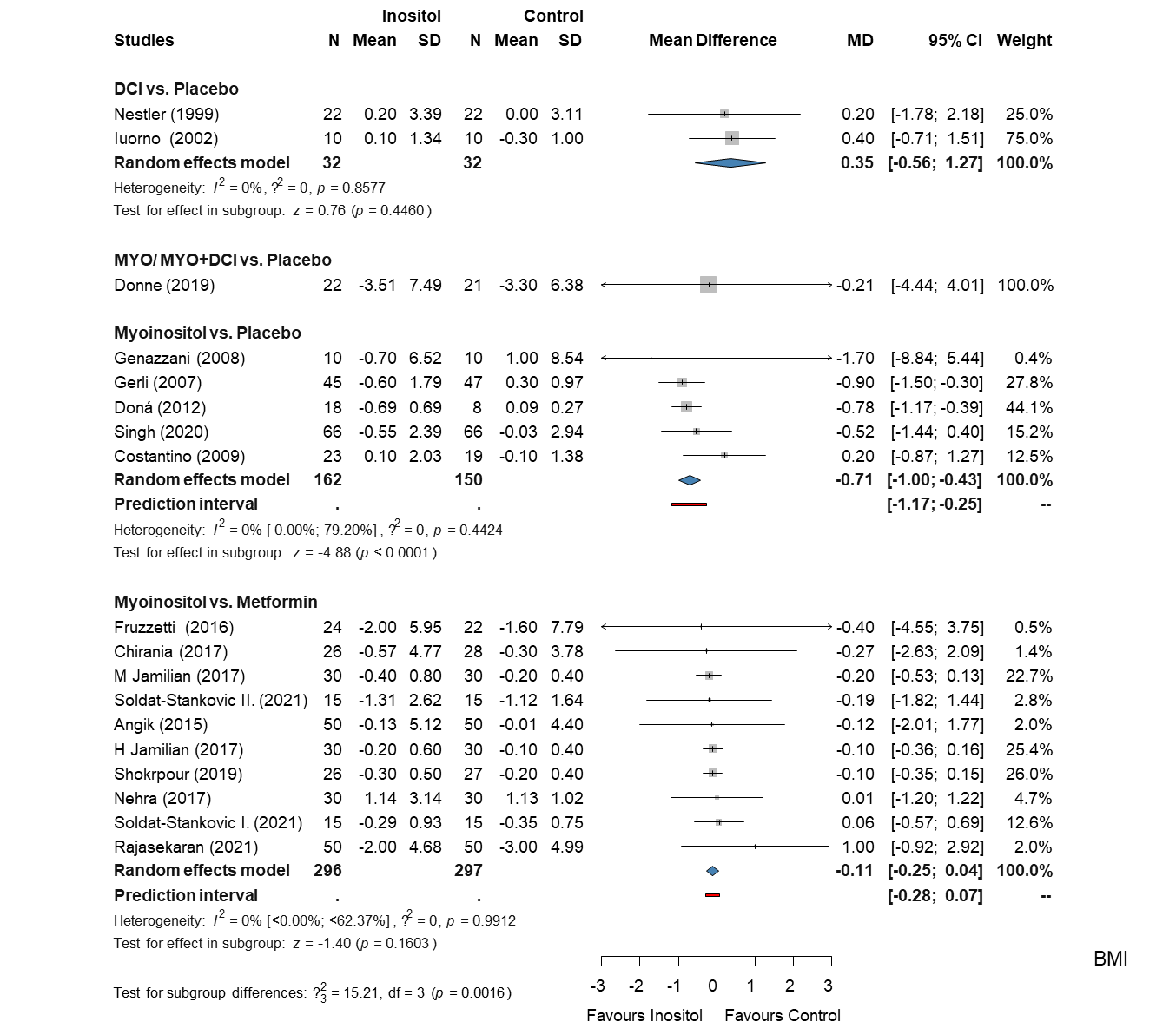
**Figure S2d.** Forest plots representing the mean difference of weight loss in the groups treated with inositols compared to placebo or metformin.

Soldat-Stankovic I. (2021) : BMI < 25kg/m^2^  ; Soldat-Stankovic II. (2021): BMI > 25kg/m^2^


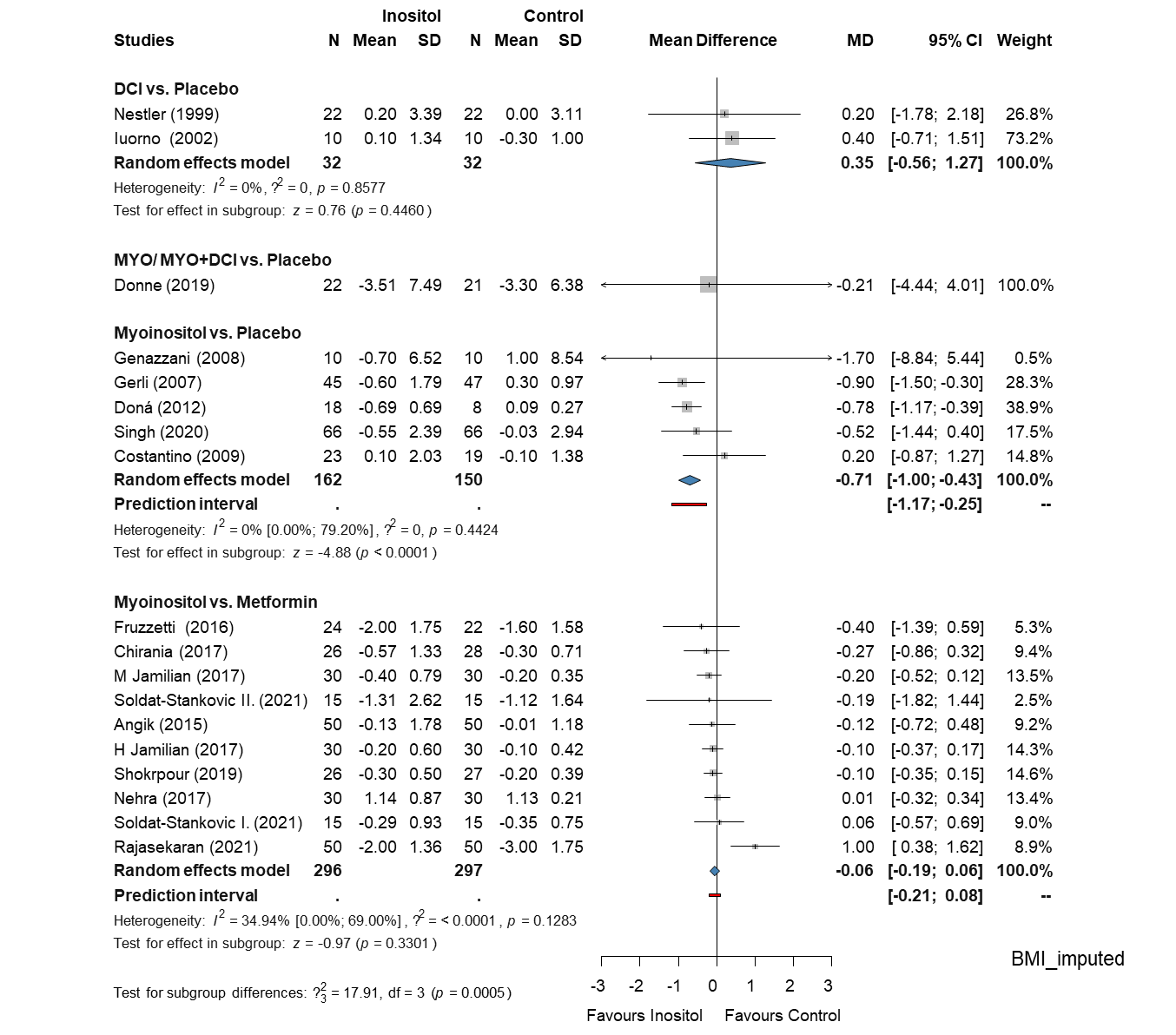
**Figure S2e.** Forest plots representing the mean difference of weight loss in the groups treated with inositols compared to placebo or metformin with imputed correlations.

Soldat-Stankovic I. (2021) : BMI < 25kg/m^2^  ; Soldat-Stankovic II. (2021): BMI > 25kg/m^2^

**Figure S3a.** Forest plots representing the mean difference of total testosterone levels in the groups treated with different inositol stereoisomers compared to placebo.


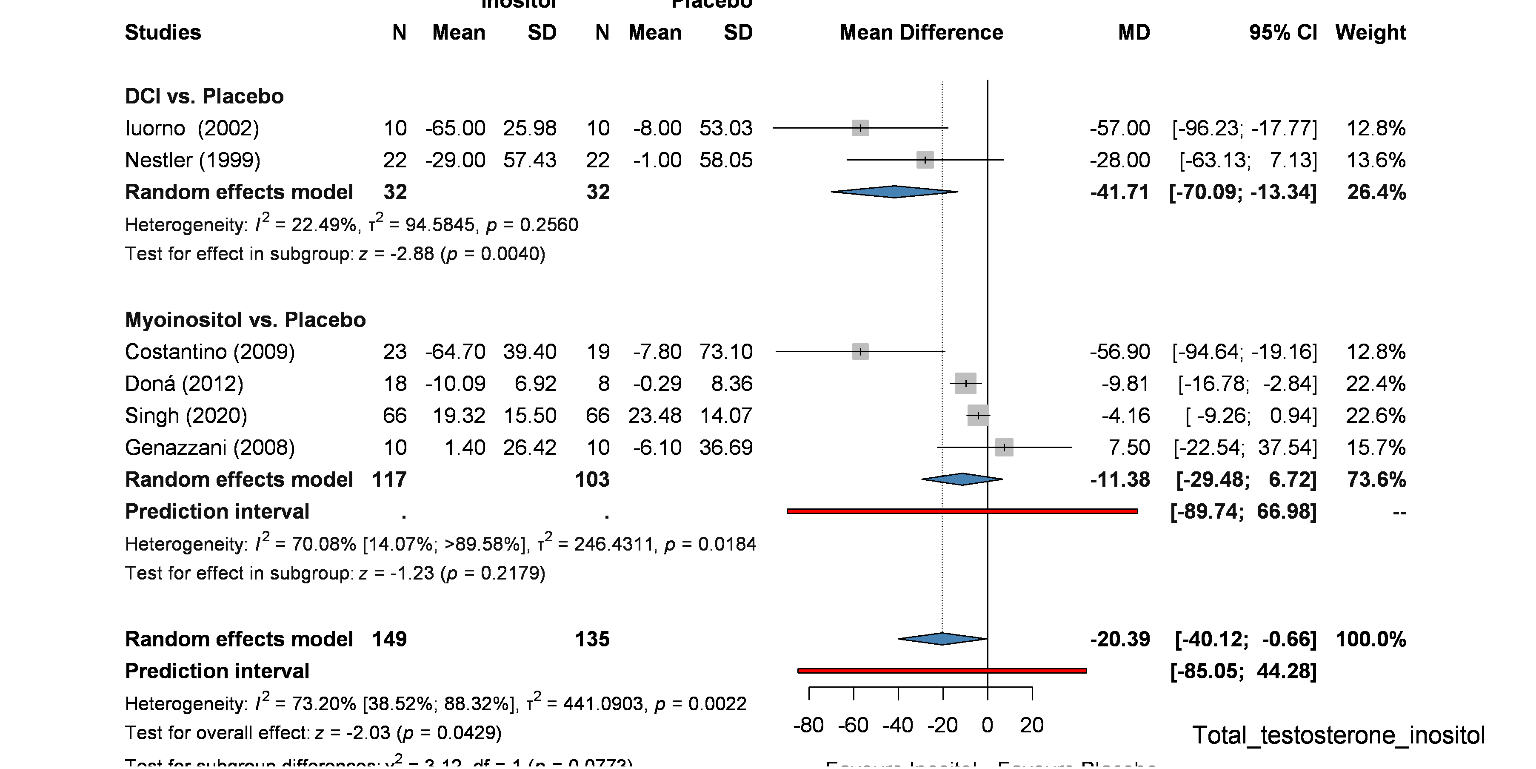


**Figure S3b.** Forest plots representing the mean difference of total testosterone levels in the groups treated with inositols compared to metformin.


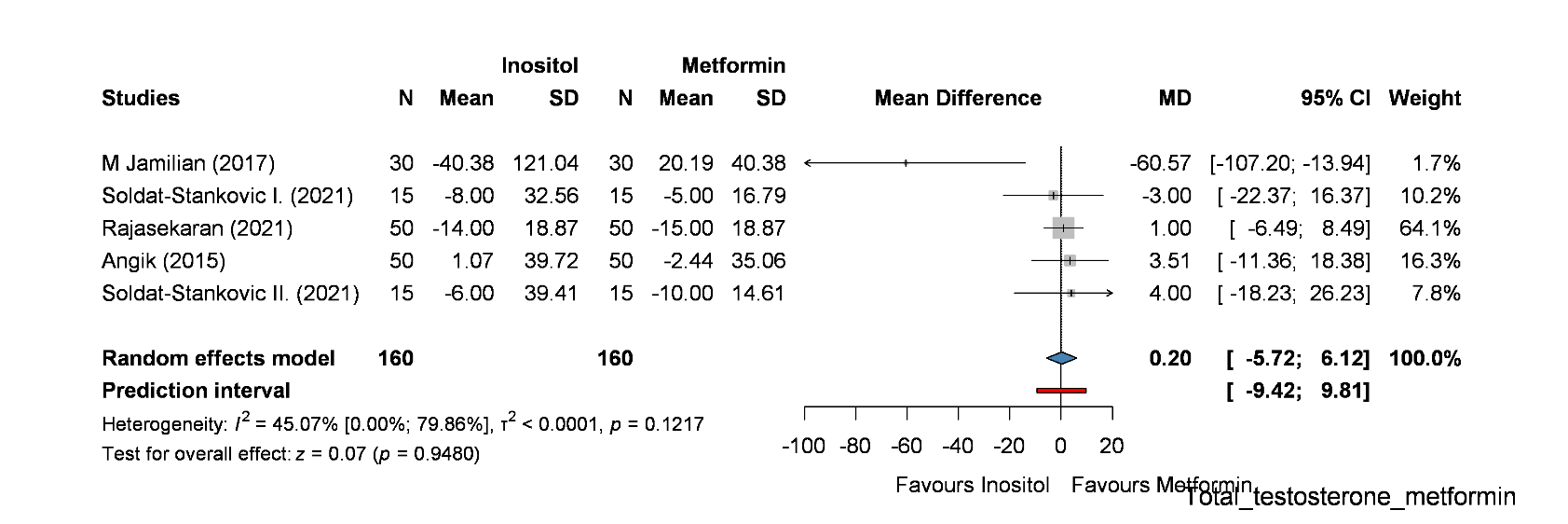


Soldat-Stankovic I. (2021) : BMI < 25kg/m^2^  ; Soldat-Stankovic II. (2021): BMI > 25kg/m^2^

**Figure S3c.** Forest plots representing the mean difference of total testosterone levels in the groups treated with inositols compared to placebo or metformin.


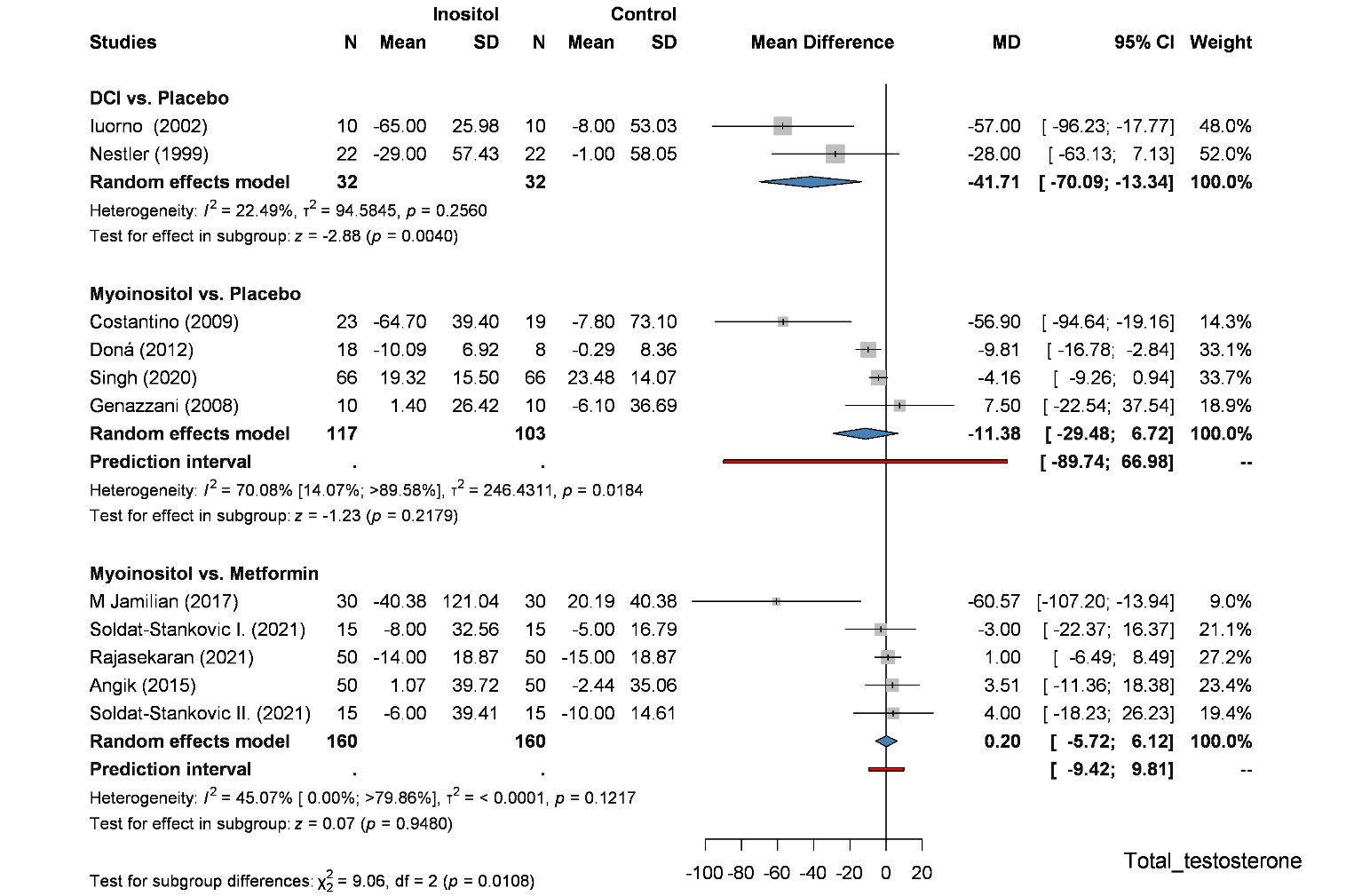


Soldat-Stankovic I. (2021) : BMI < 25kg/m^2^  ; Soldat-Stankovic II. (2021): BMI > 25kg/m^2^

**Figure S3d.** Forest plots representing the mean difference of total testosterone levels in the groups treated with inositols compared to placebo or metformin. (Summary of different inositols into one ‘combined inositols’group.)

**
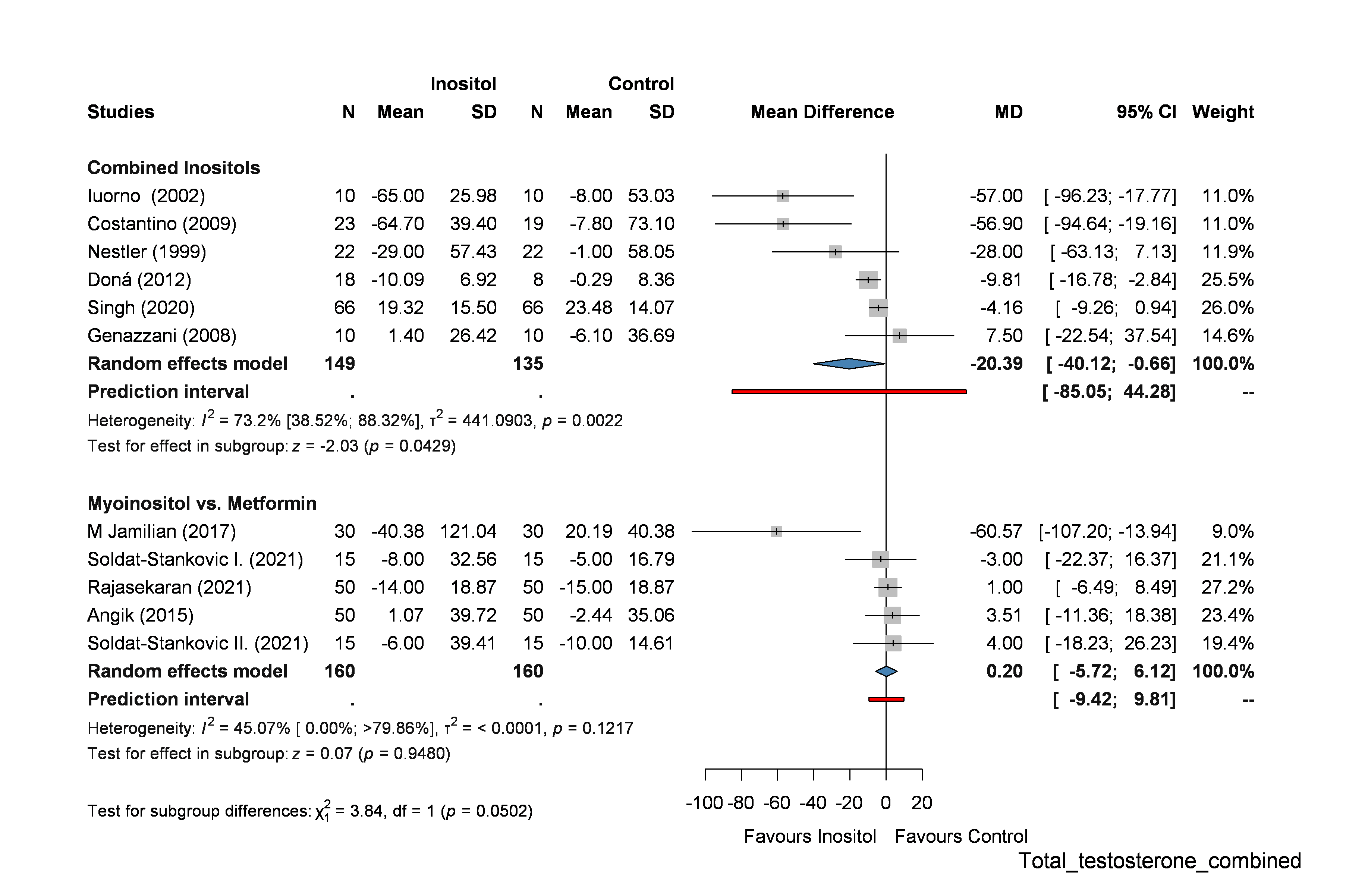
**

Soldat-Stankovic I. (2021) : BMI < 25kg/m^2^  ; Soldat-Stankovic II. (2021): BMI > 25kg/m^2^

**Figure S4.** Forest plots representing the mean difference of free testosterone levels in the groups treated with inositols compared to placebo.


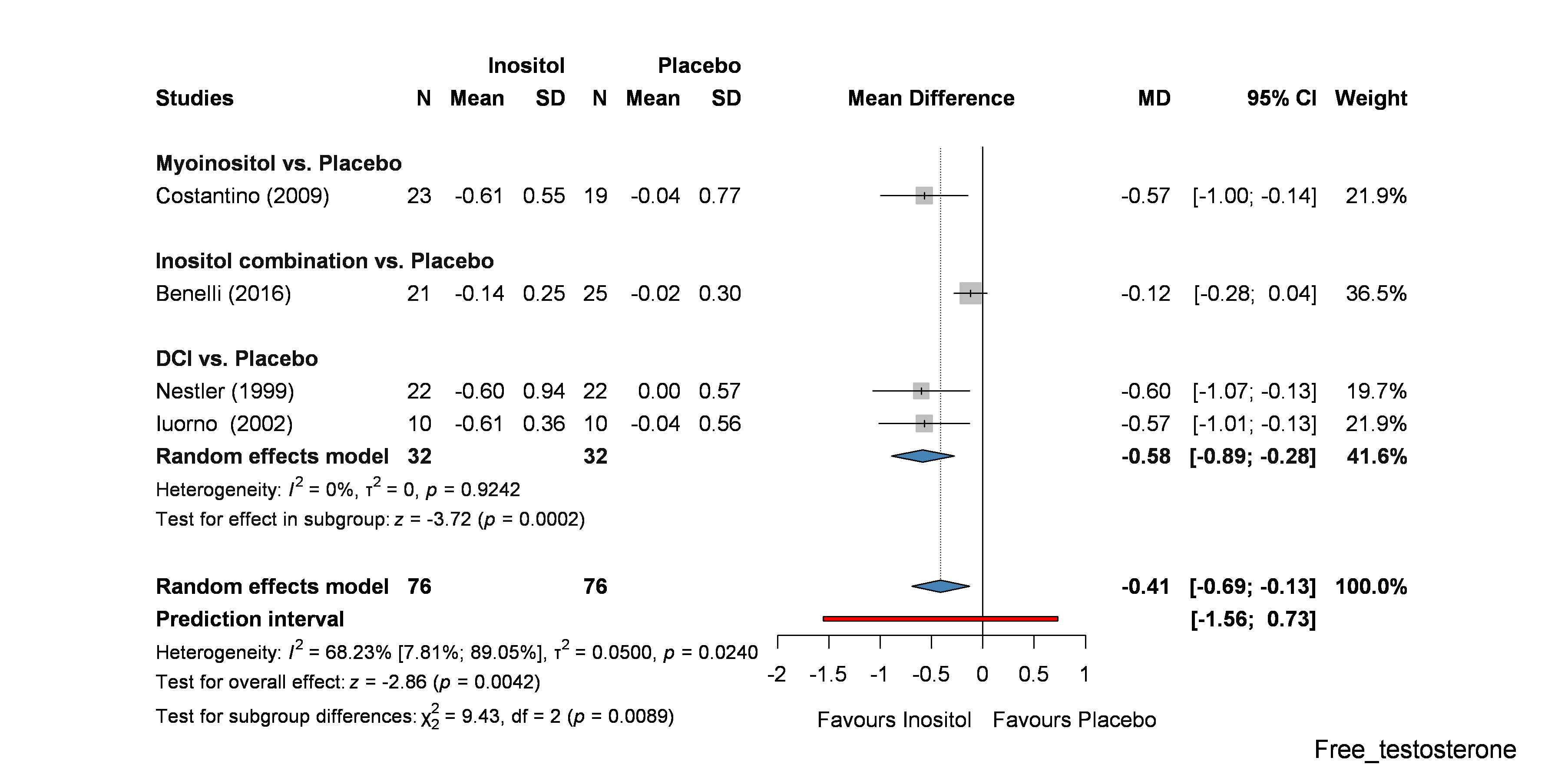


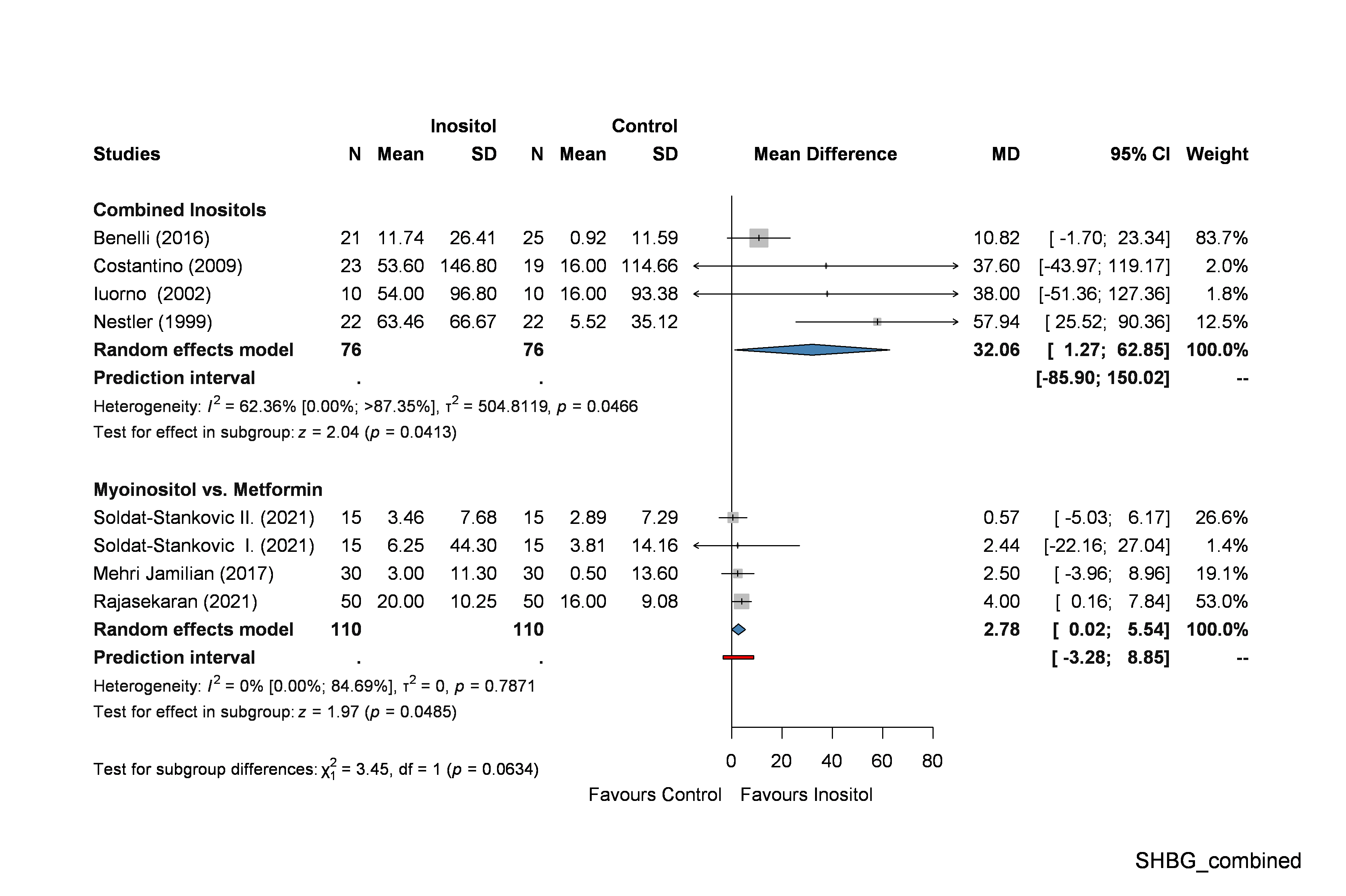
**Figure S5a.** Forest plots representing the mean difference of SHBG levels in the groups treated with inositols compared to placebo or metformin. (Summary of different inositols into one ‘combined inositols’group.

Soldat-Stankovic I. (2021) : BMI < 25kg/m^2^  ; Soldat-Stankovic II. (2021): BMI > 25kg/m^2^


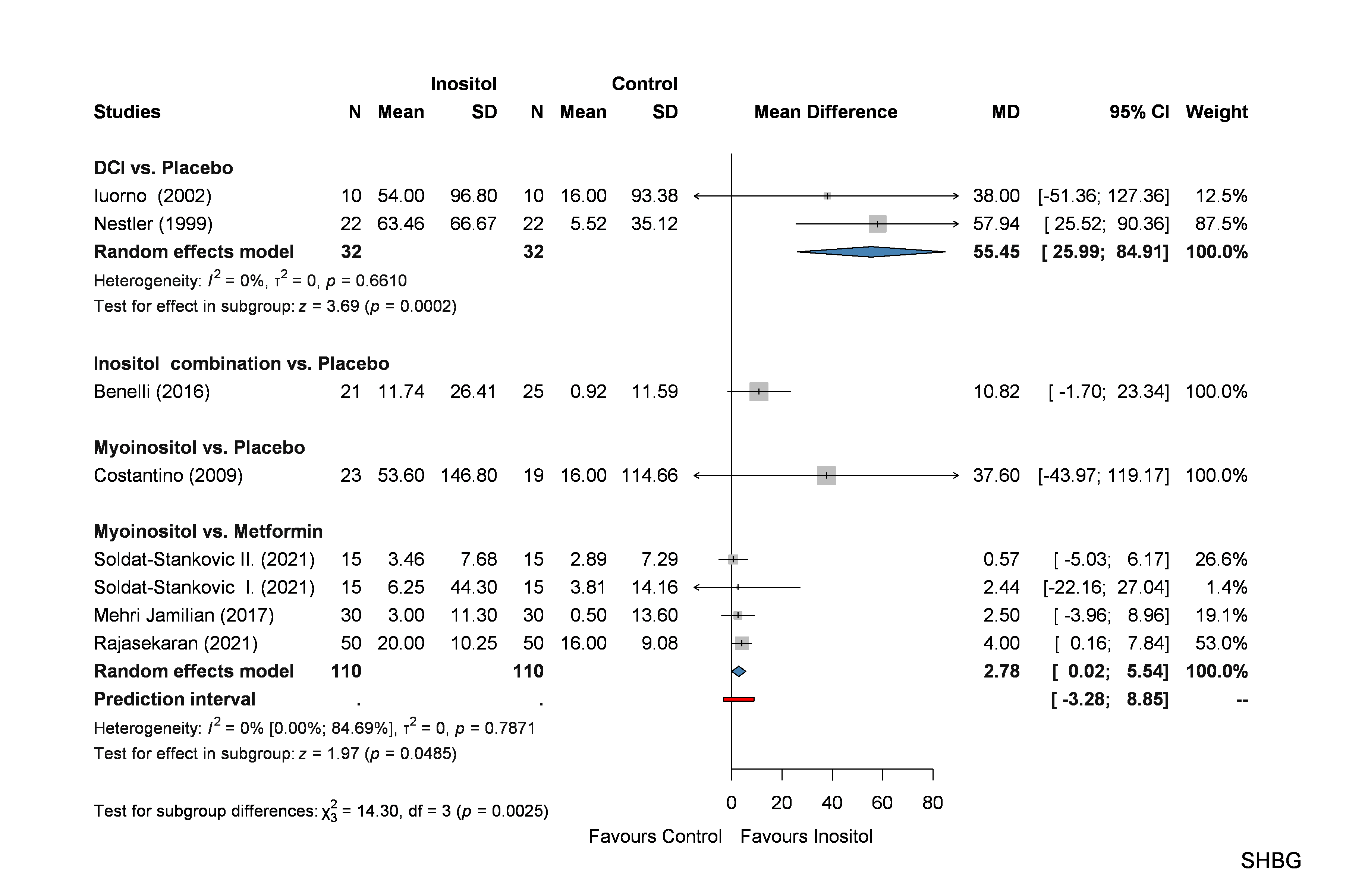
**Figure S5b.** Forest plots representing the mean difference of SHBG levels in the groups treated with inositols compared to placebo or metformin.

Soldat-Stankovic I. (2021) : BMI < 25kg/m^2^  ; Soldat-Stankovic II. (2021): BMI > 25kg/m^2^


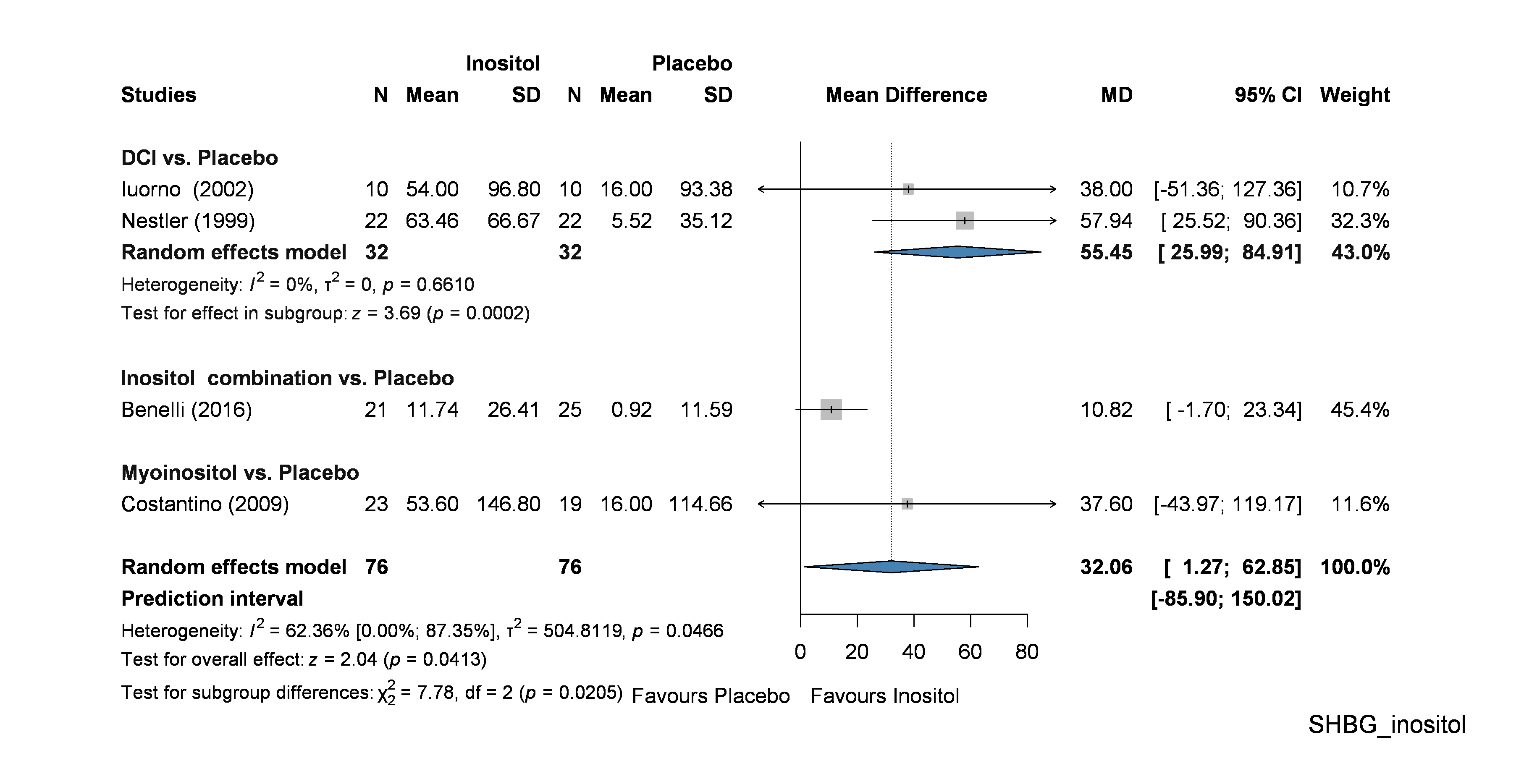
**Figure S5c.** Forest plots representing the mean difference of SHBG levels in the groups treated with different inositol stereoismers compared to placebo.


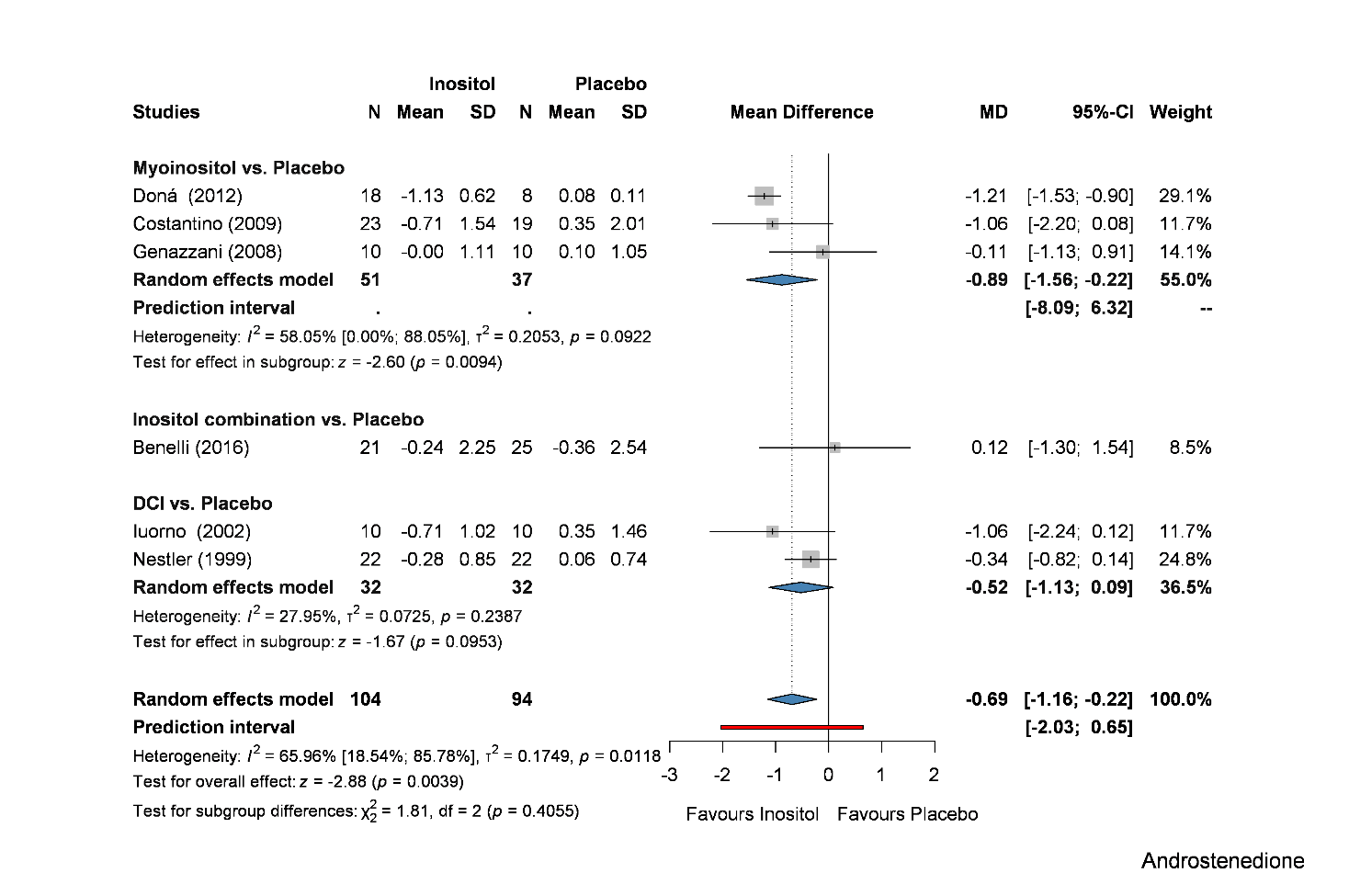
**Figure S6.** Forest plots representing the mean difference of androstenedione levels in the groups treated with inositols compared to placebo.


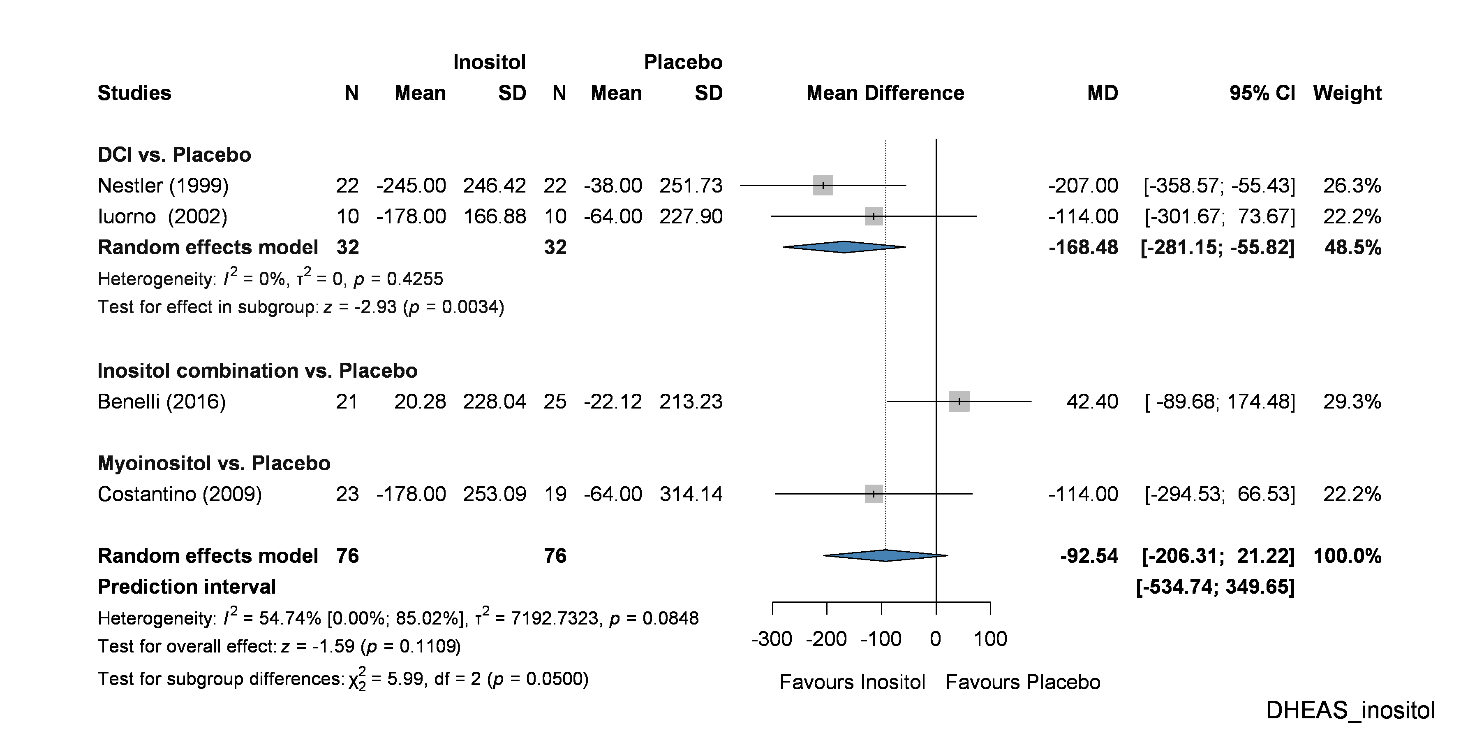
**Figure S7a.** Forest plots representing the mean difference of DHEAS levels in the groups treated with different inositol stereoisomers compared to placebo.

**Figure S7b.** Forest plots representing the mean difference of DHEAS levels in the groups treated with inositols compared to placebo or metformin.


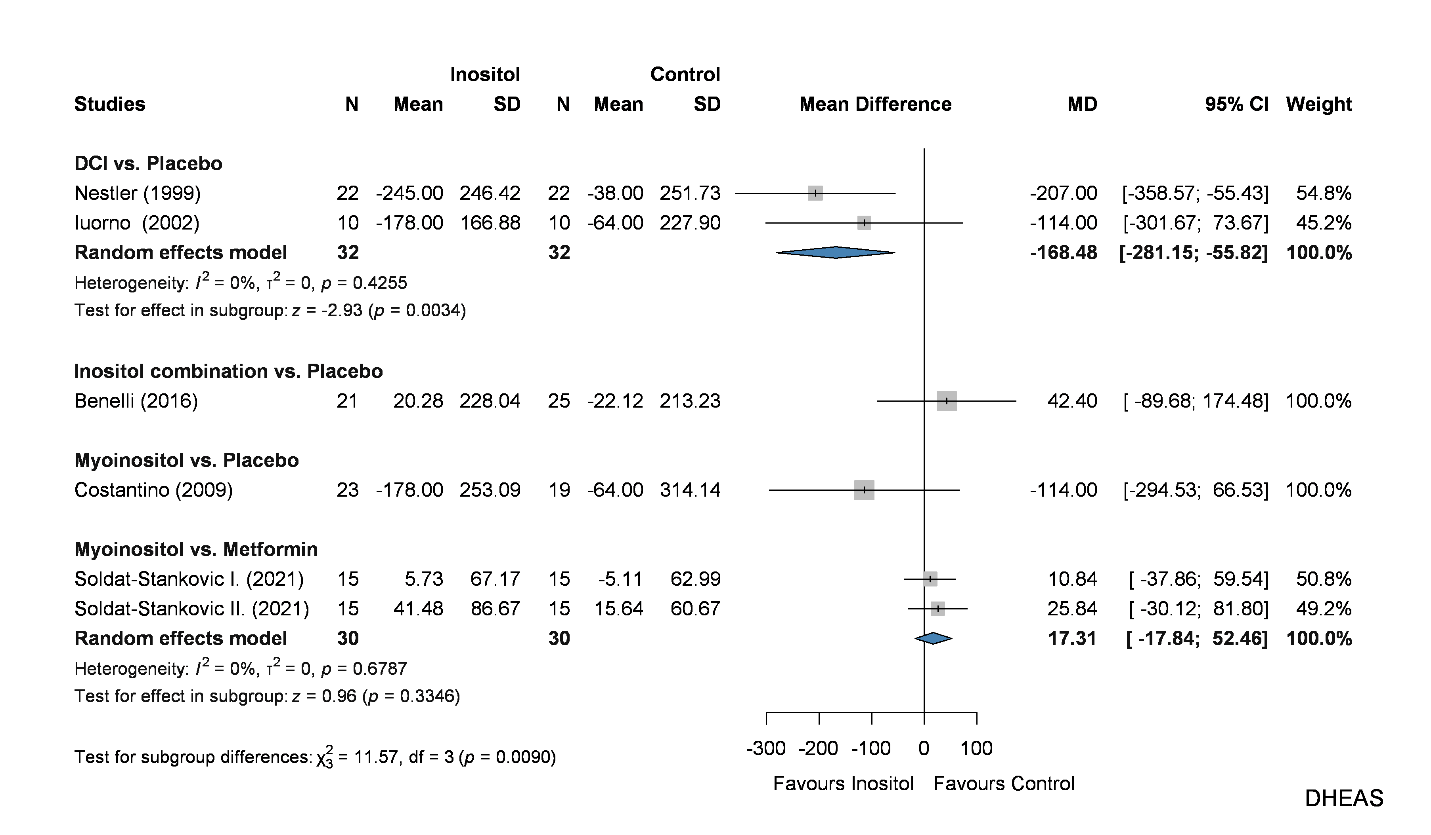


Soldat-Stankovic I. (2021) : BMI < 25kg/m^2^  ; Soldat-Stankovic II. (2021): BMI > 25kg/m^2^

**Figure S7c.** Forest plots representing the mean difference of DHEAS levels in the groups treated with inositols compared to placebo or metformin. (Summary of different inositols into one ‘combined inositols’group.)


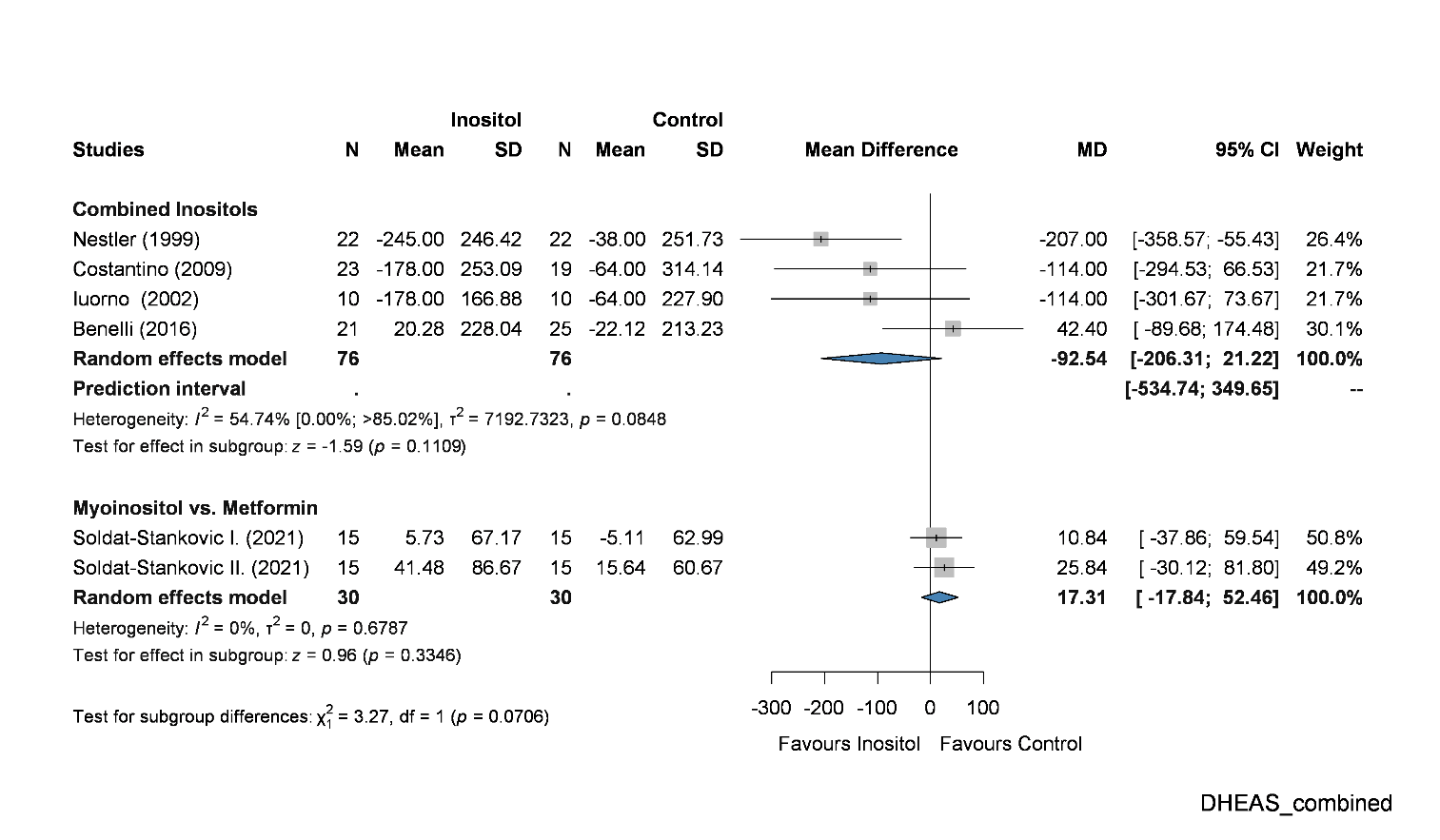


Soldat-Stankovic I. (2021) : BMI < 25kg/m^2^  ; Soldat-Stankovic II. (2021): BMI > 25kg/m^2^

**Figure S8.** Forest plots representing the mean difference of Ferriman–Gallwey score in the groups treated with inositols compared to placebo or metformin.


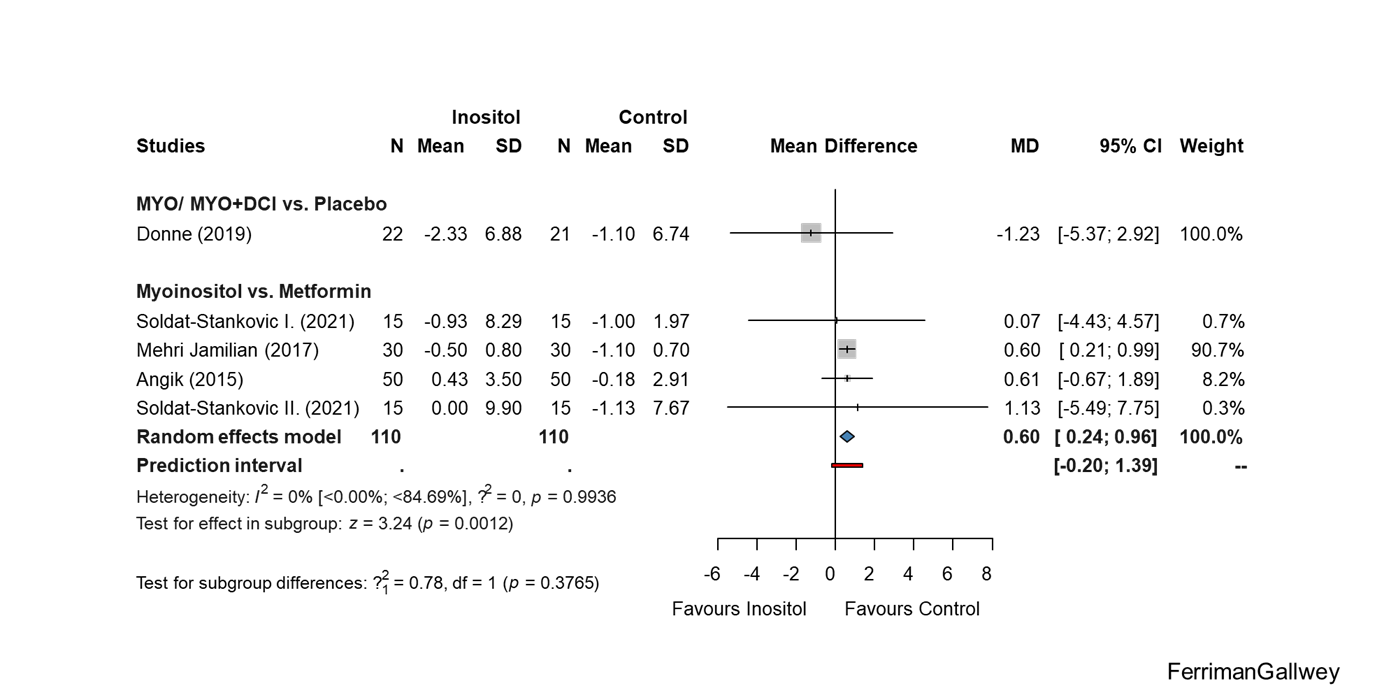


Soldat-Stankovic I. (2021) : BMI < 25kg/m^2^  ; Soldat-Stankovic II. (2021): BMI > 25kg/m^2^


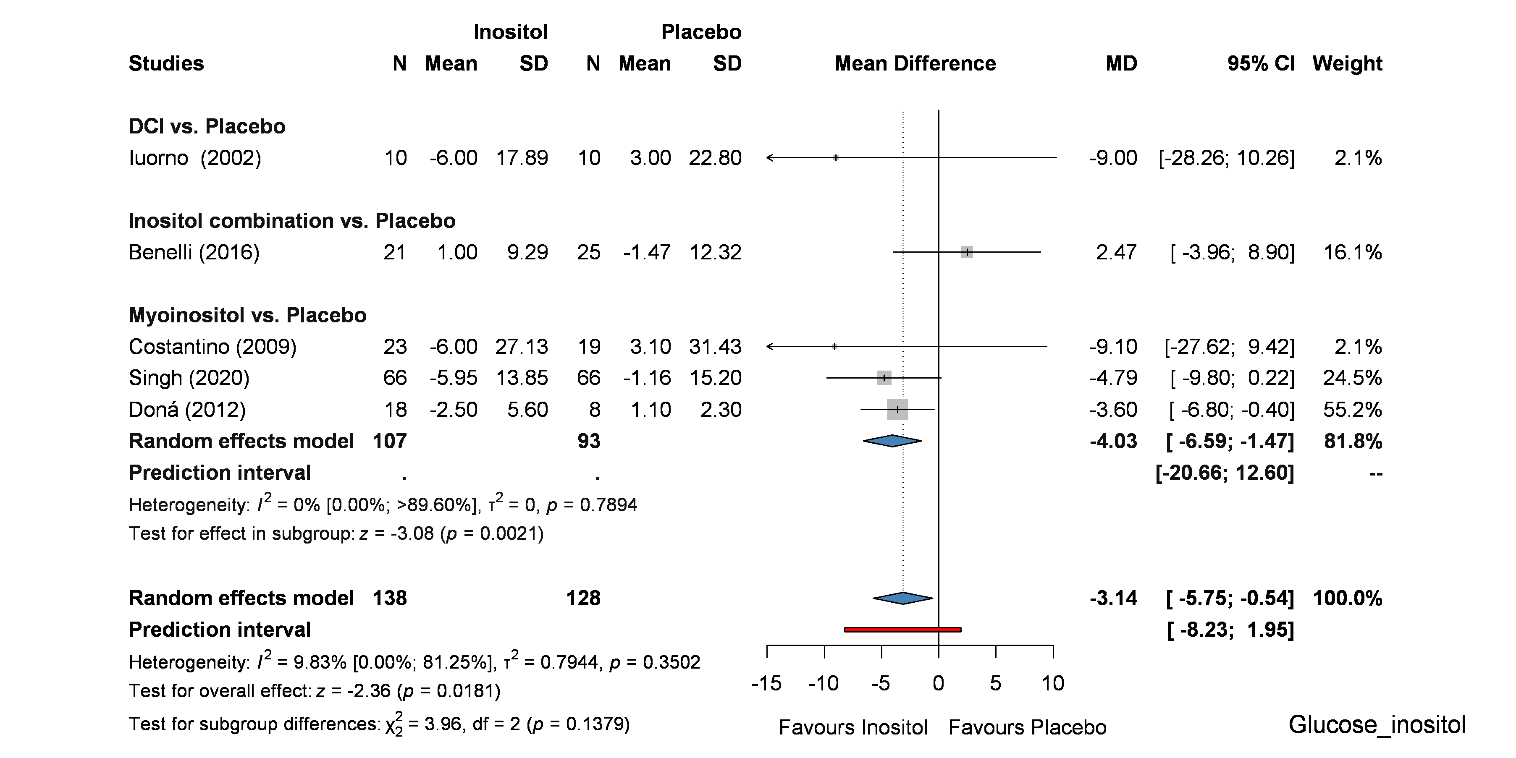
**Figure S9a.** Forest plots representing the mean difference of fasting plasma glucose in the groups treated with different inositol stereoisomers compared to placebo.

**Figure S9b.** Forest plots representing the mean difference of fasting plasma glucose in the groups treated with inositols compared to metformin.


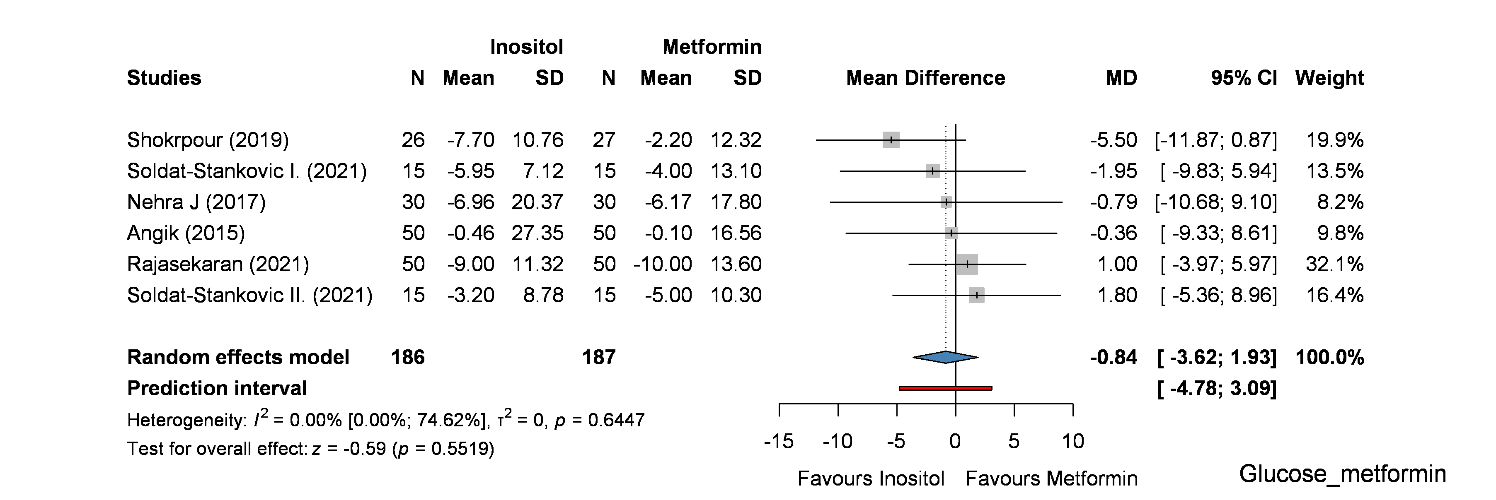


Soldat-Stankovic I. (2021) : BMI < 25kg/m^2^  ; Soldat-Stankovic II. (2021): BMI > 25kg/m^2^

**Figure S9c.** Forest plots representing the mean difference of fasting plasma glucose in the groups treated with inositols compared to placebo or metformin.


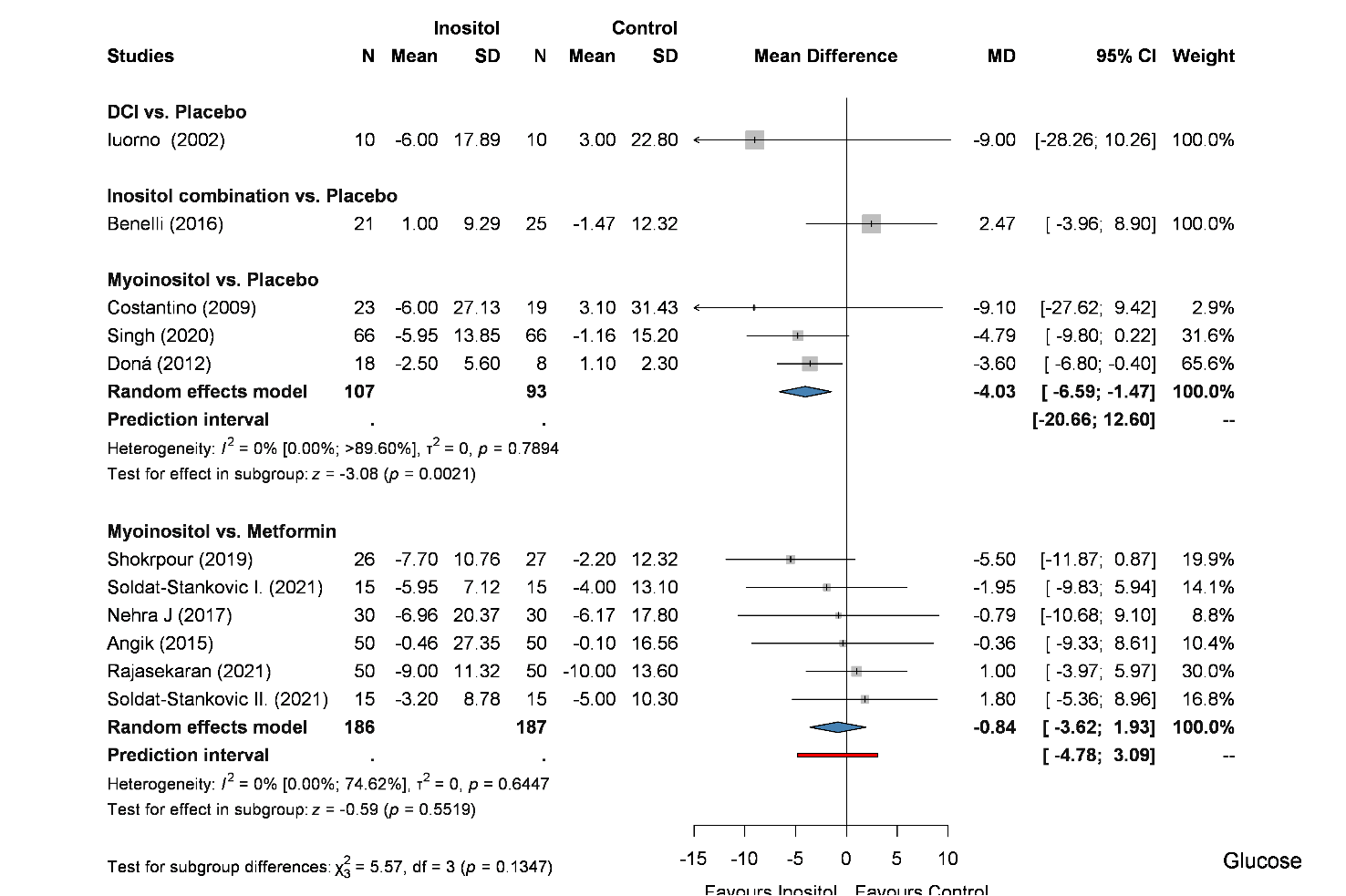


Soldat-Stankovic I. (2021) : BMI < 25kg/m^2^  ; Soldat-Stankovic II. (2021): BMI > 25kg/m^2^


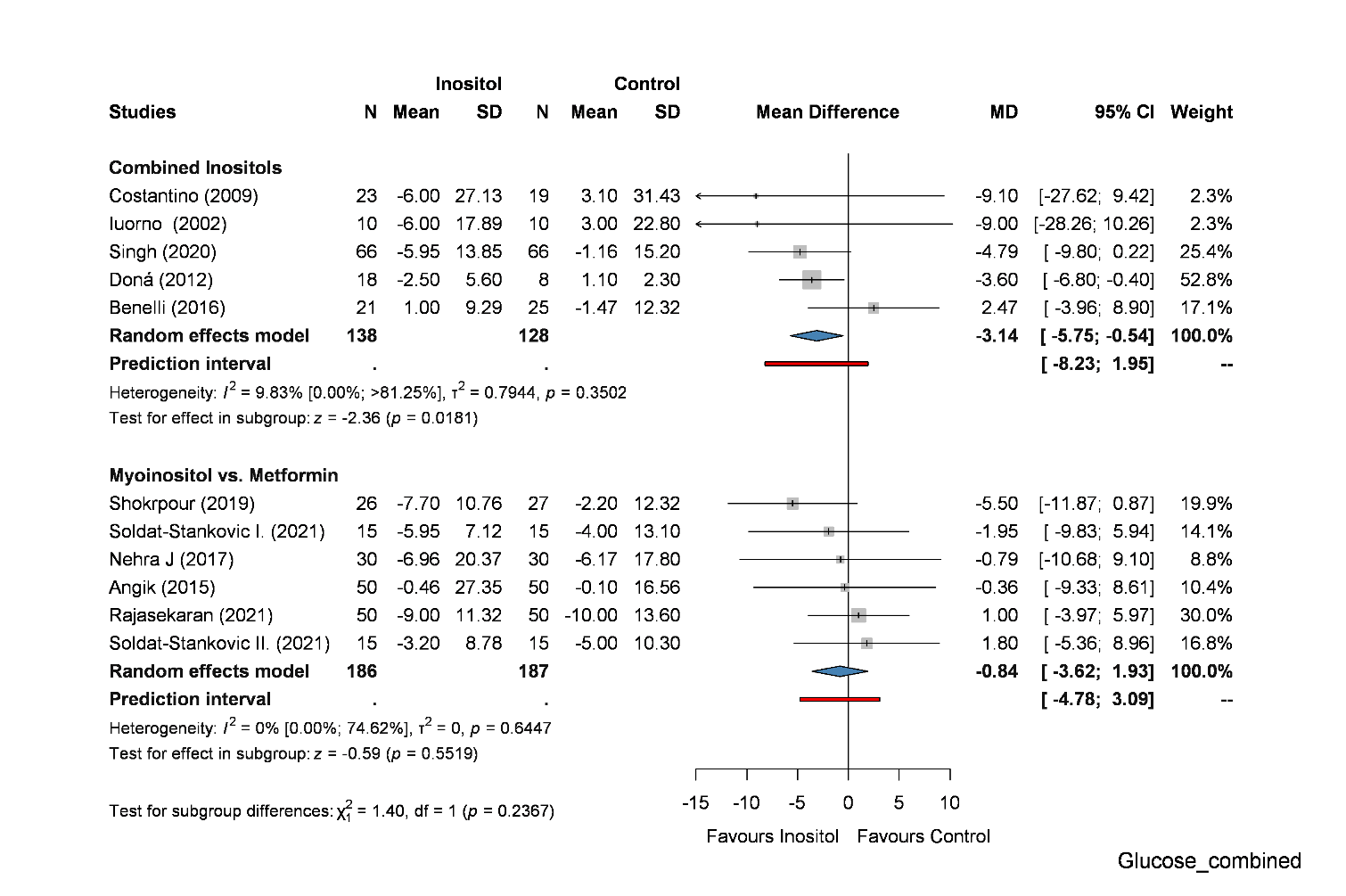
**Figure S9d.** Forest plots representing the mean difference of fasting plasma glucose in the groups treated with inositols compared to placebo or metformin. (Summary of different inositols into one ‘combined inositols’group.)

Soldat-Stankovic I. (2021) : BMI < 25kg/m^2^  ; Soldat-Stankovic II. (2021): BMI > 25kg/m^2^

**Figure S10a.** Forest plots representing the mean difference of fasting plasma insulin in the groups treated with different inositol stereoisomers compared to placebo.


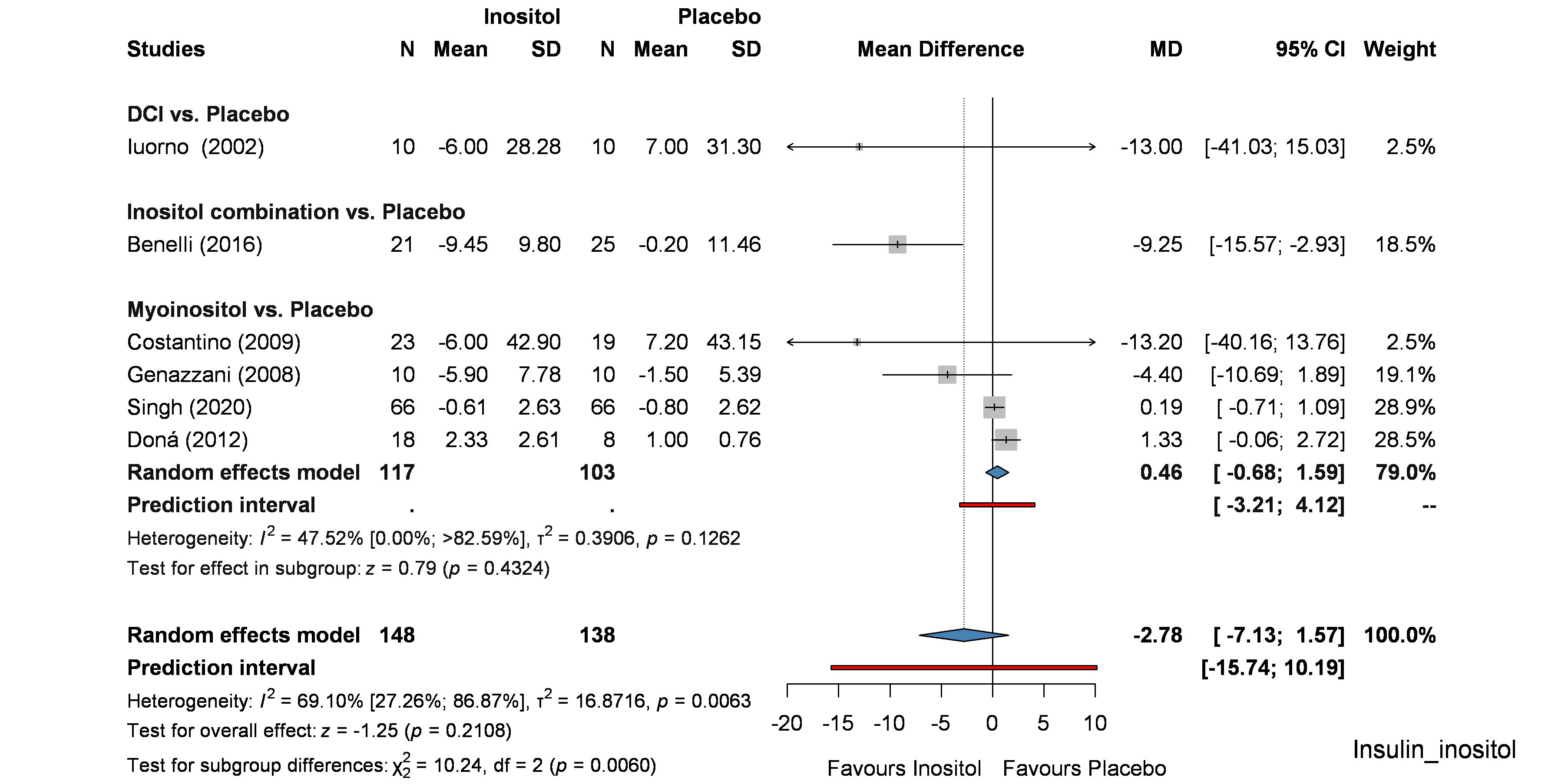


**Figure S10b.** Forest plots representing the mean difference of fasting plasma insulin in the groups treated with inositols compared to metformin.


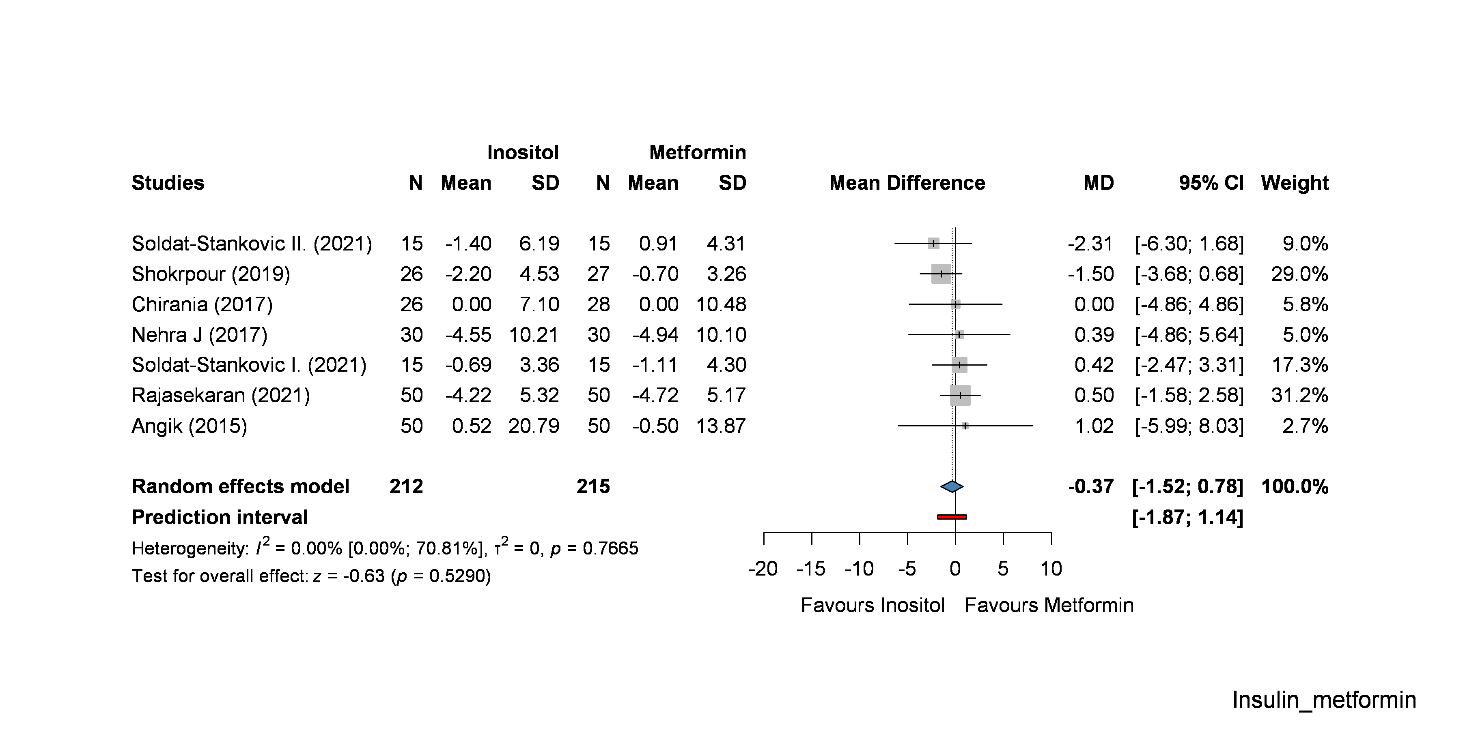


Soldat-Stankovic I. (2021) : BMI < 25kg/m^2^  ; Soldat-Stankovic II. (2021): BMI > 25kg/m^2^

**Figure S10c.** Forest plots representing the mean difference of fasting plasma insulin in the groups treated with inositols compared to placebo or metformin.


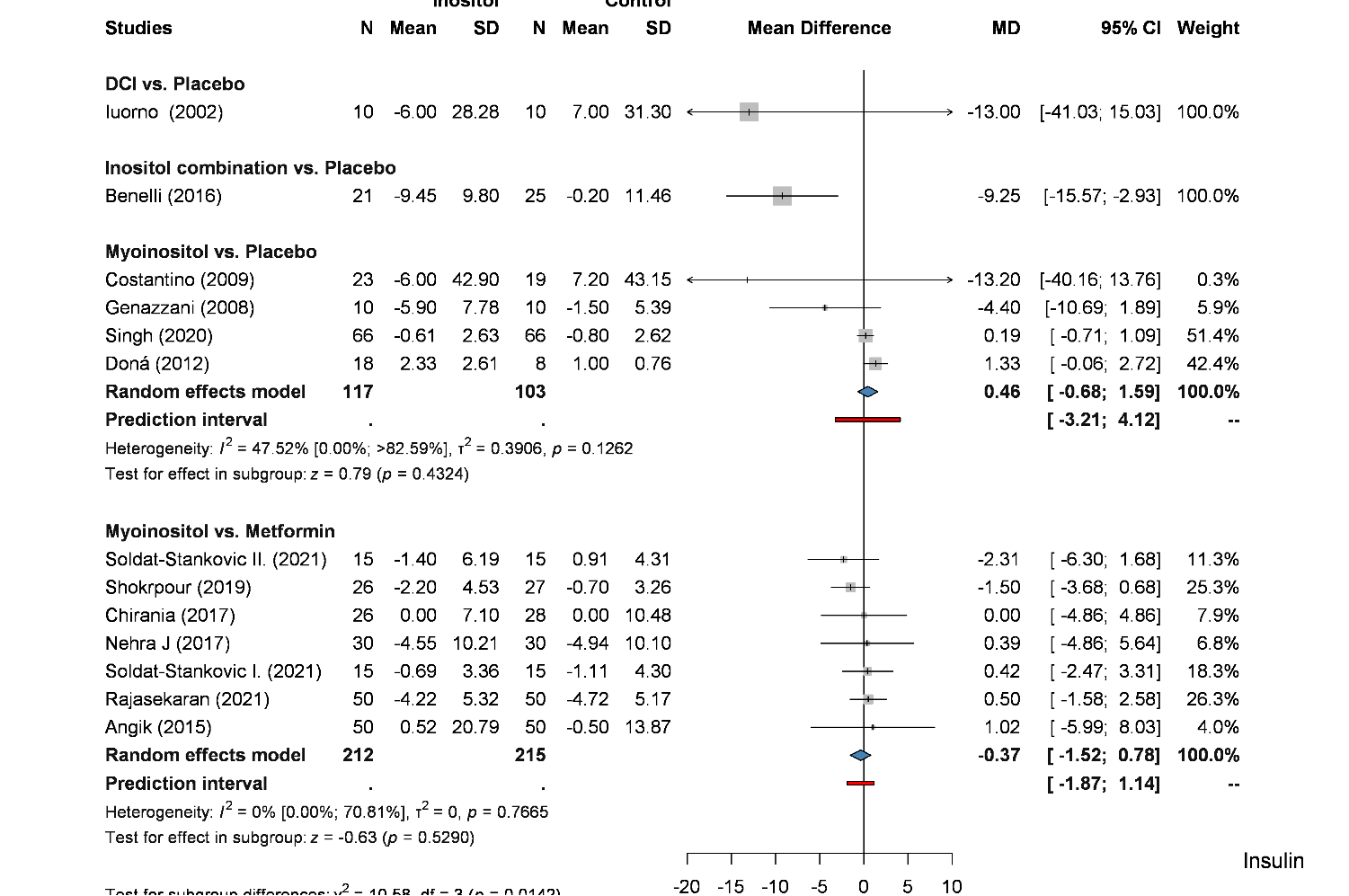


Soldat-Stankovic I. (2021) : BMI < 25kg/m^2^  ; Soldat-Stankovic II. (2021): BMI > 25kg/m^2^

**Figure S10d.** Forest plots representing the mean difference of fasting plasma insulin in the groups treated with inositols compared to placebo or metformin. (Summary of different inositols into one ‘combined inositols’group.)


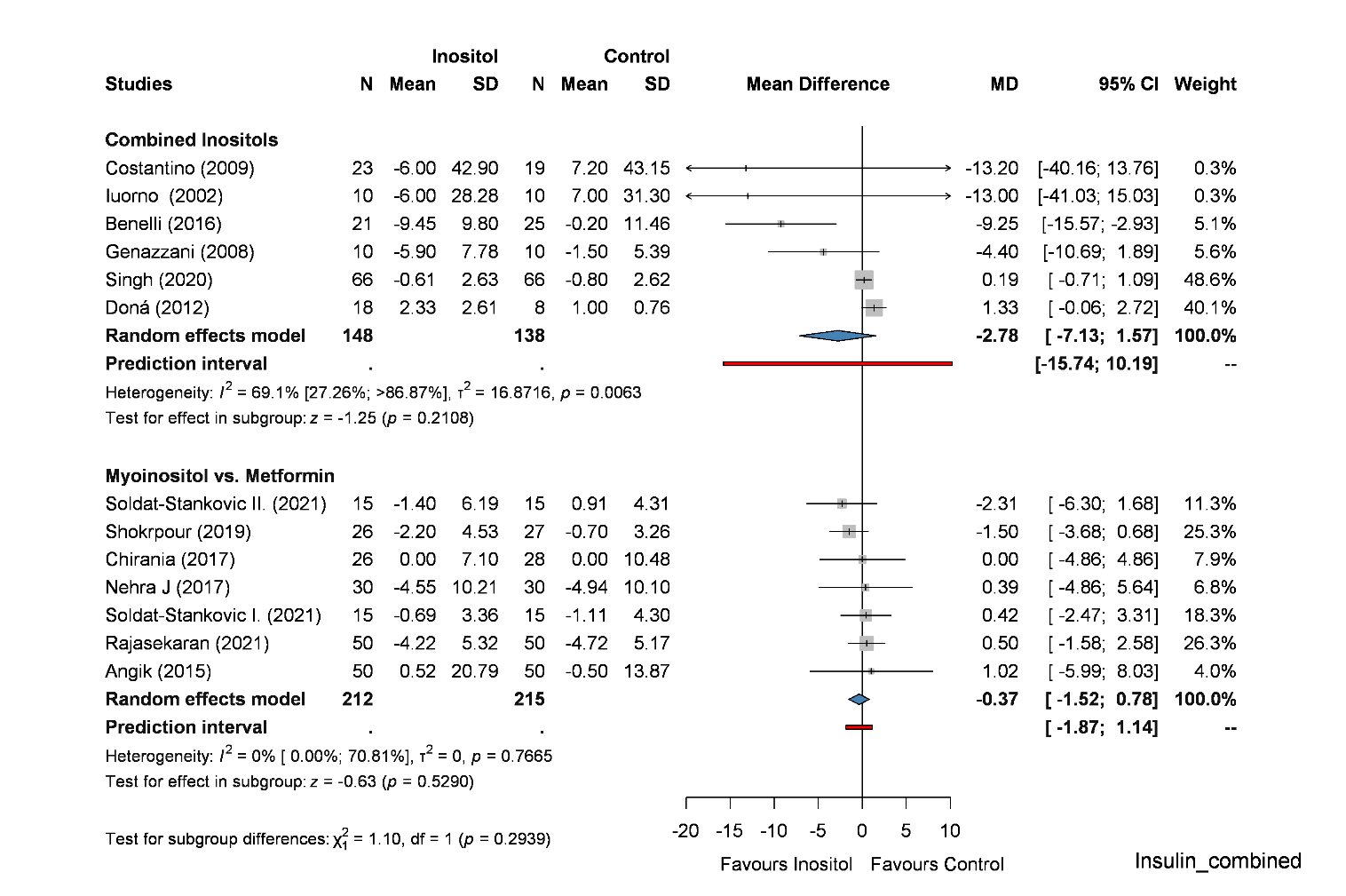


Soldat-Stankovic I. (2021) : BMI < 25kg/m^2^  ; Soldat-Stankovic II. (2021): BMI > 25kg/m^2^

**Figure S11a.** Forest plots representing the mean difference of HOMA-IR in the groups treated with different inositol stereoisomers compared to placebo.


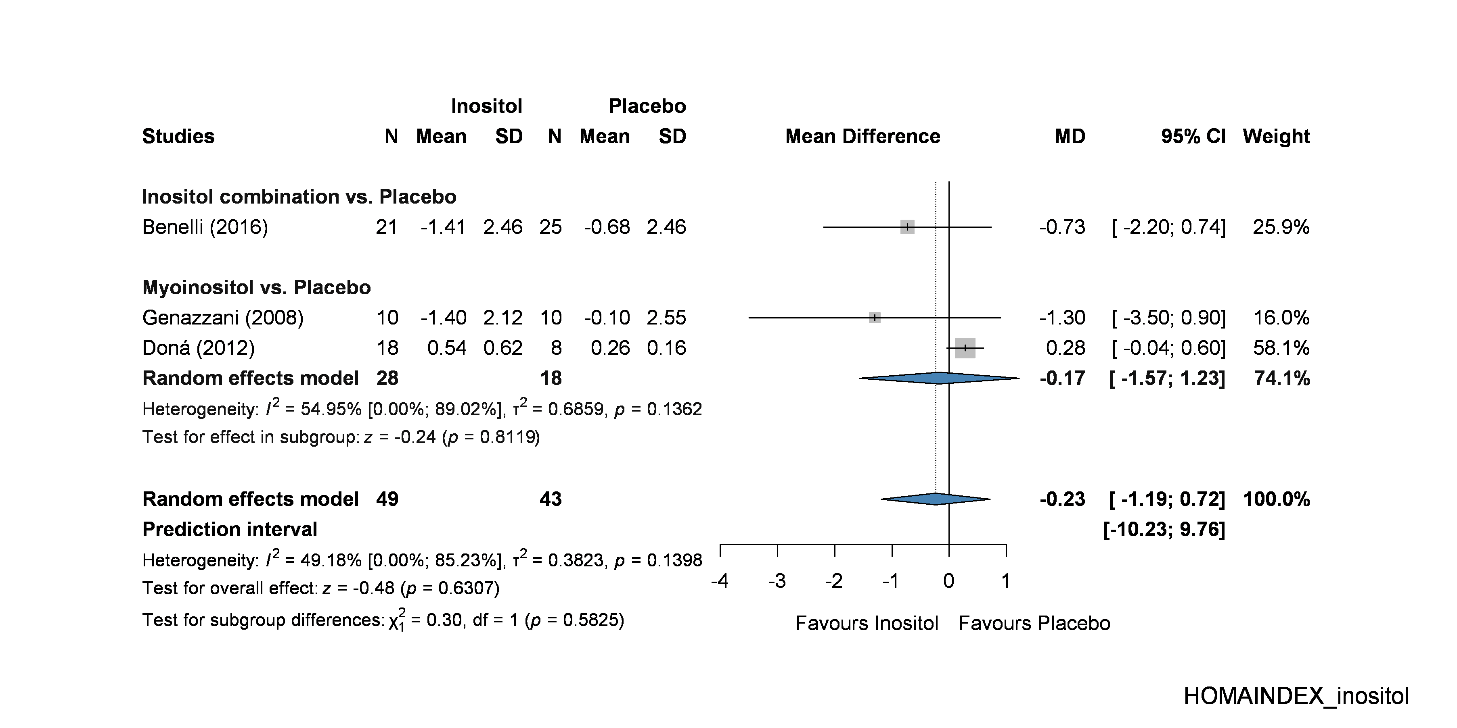


**Figure S11b.** Forest plots representing the mean difference of HOMA-IR in the groups treated with inositols compared to metformin.


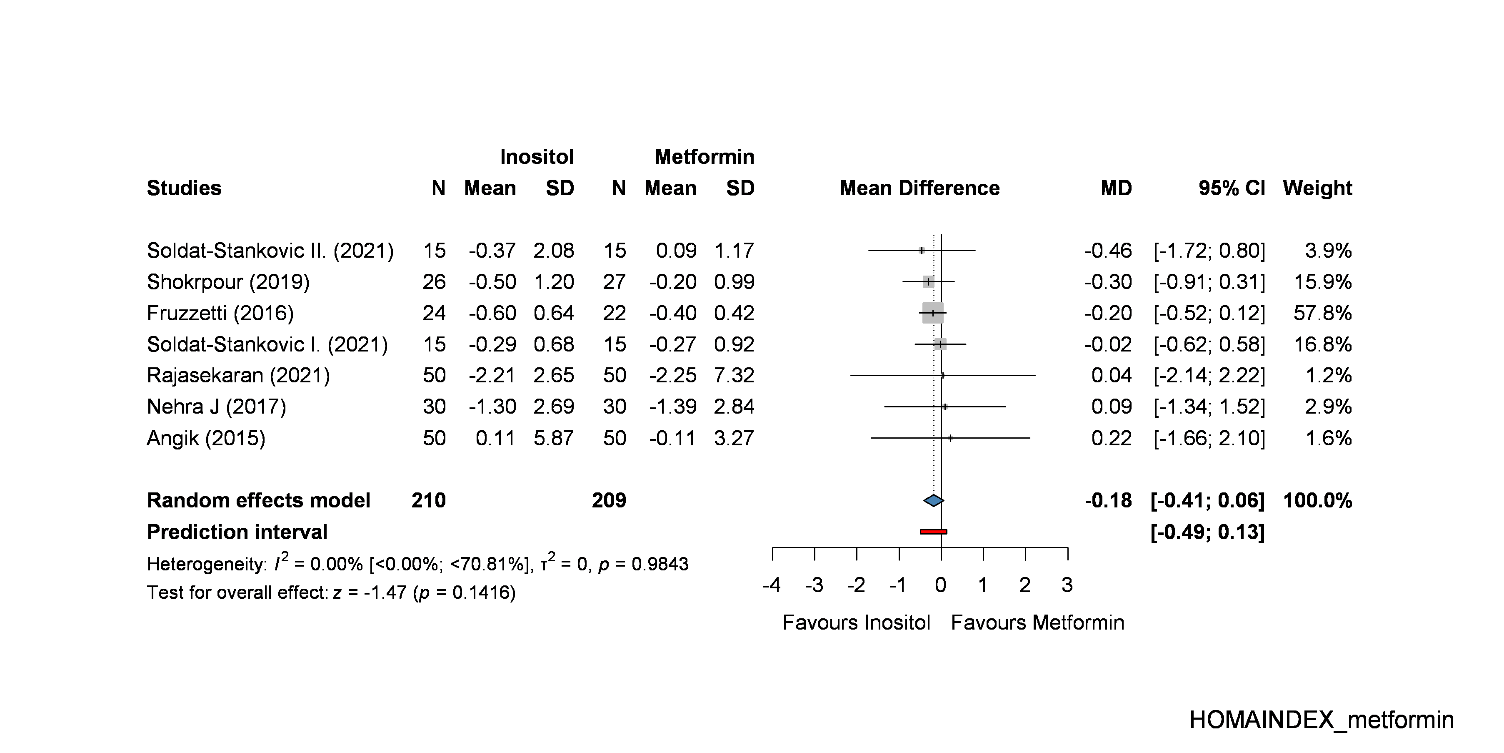


Soldat-Stankovic I. (2021) : BMI < 25kg/m^2^  ; Soldat-Stankovic II. (2021): BMI > 25kg/m^2^

**Figure S11c.** Forest plots representing the mean difference of HOMA-IR in the groups treated with inositols compared to placebo or metformin.


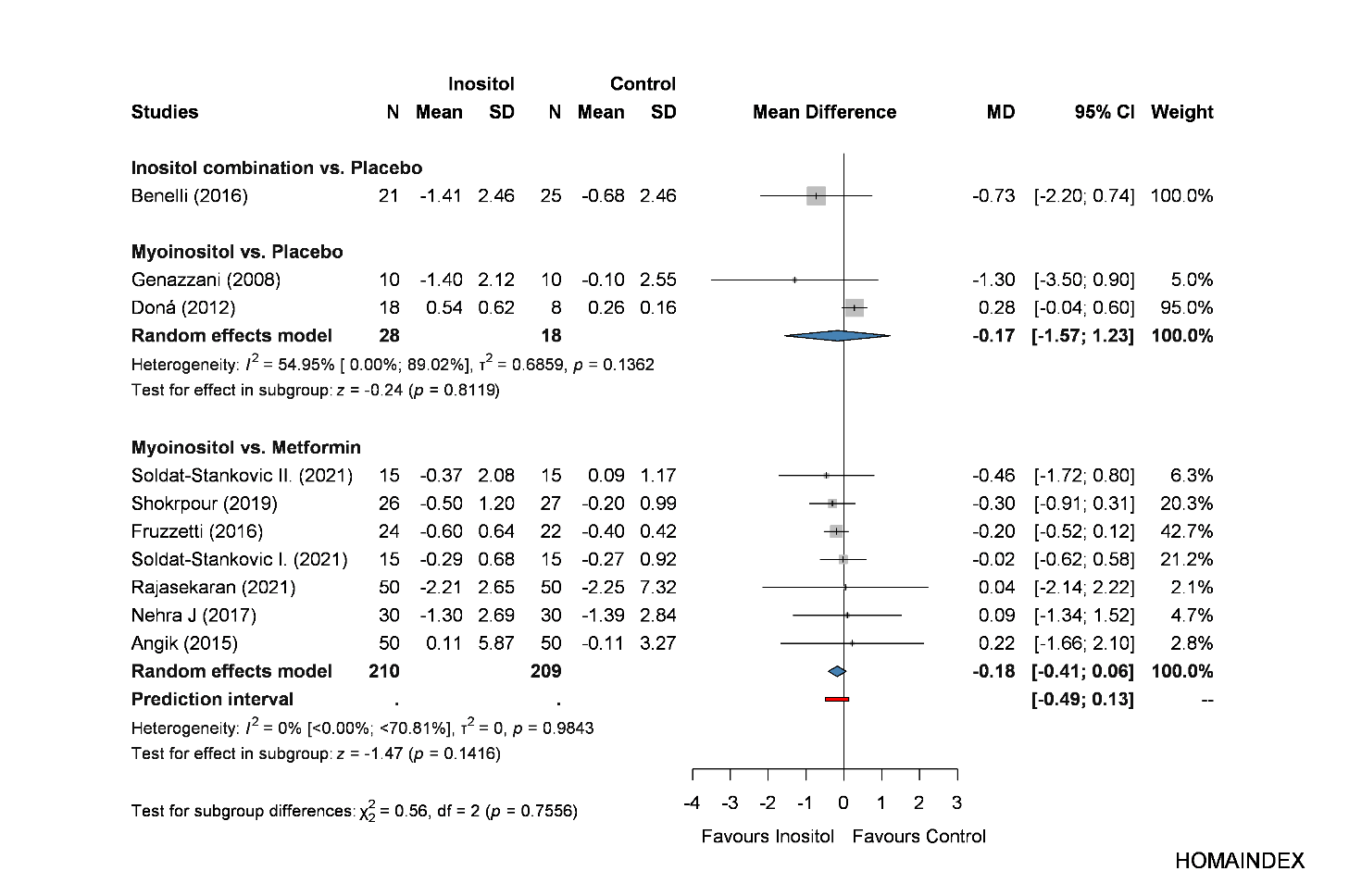


Soldat-Stankovic I. (2021) : BMI < 25kg/m^2^  ; Soldat-Stankovic II. (2021): BMI > 25kg/m^2^

**Figure S11d.** Forest plots representing the mean difference of HOMA-IR in the groups treated with inositols compared to placebo or metformin. (Summary of different inositols into one ‘combined inositols’group).


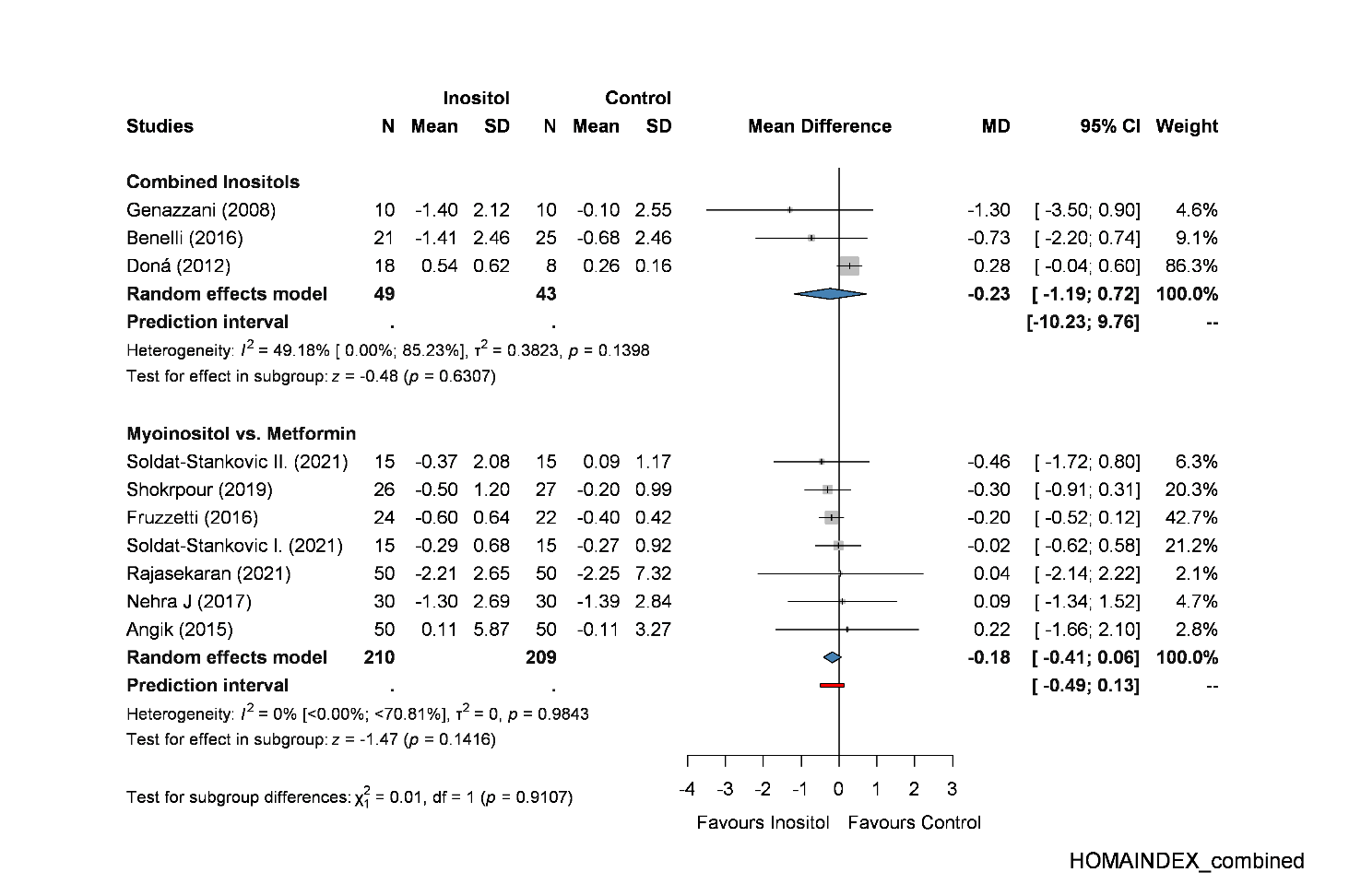


Soldat-Stankovic I. (2021) : BMI < 25kg/m^2^  ; Soldat-Stankovic II. (2021): BMI > 25kg/m^2^

**Figure S12a.** Forest plots representing the mean difference of AUC Glucose in the groups treated with different inositol stereoisomers compared to placebo.


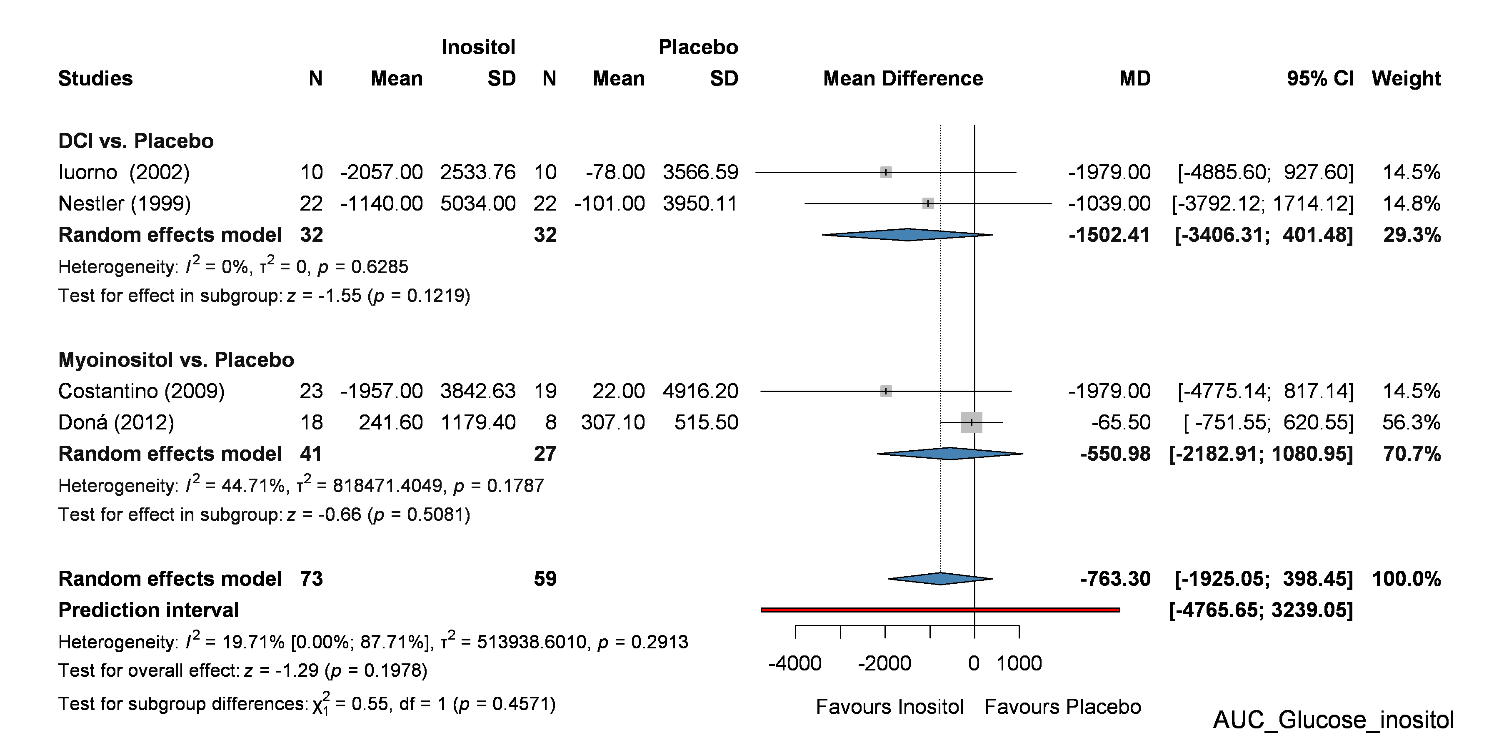


**Figure S12b**. Forest plots representing the mean difference of AUC Glucose in the groups treated with inositols compared to metformin.


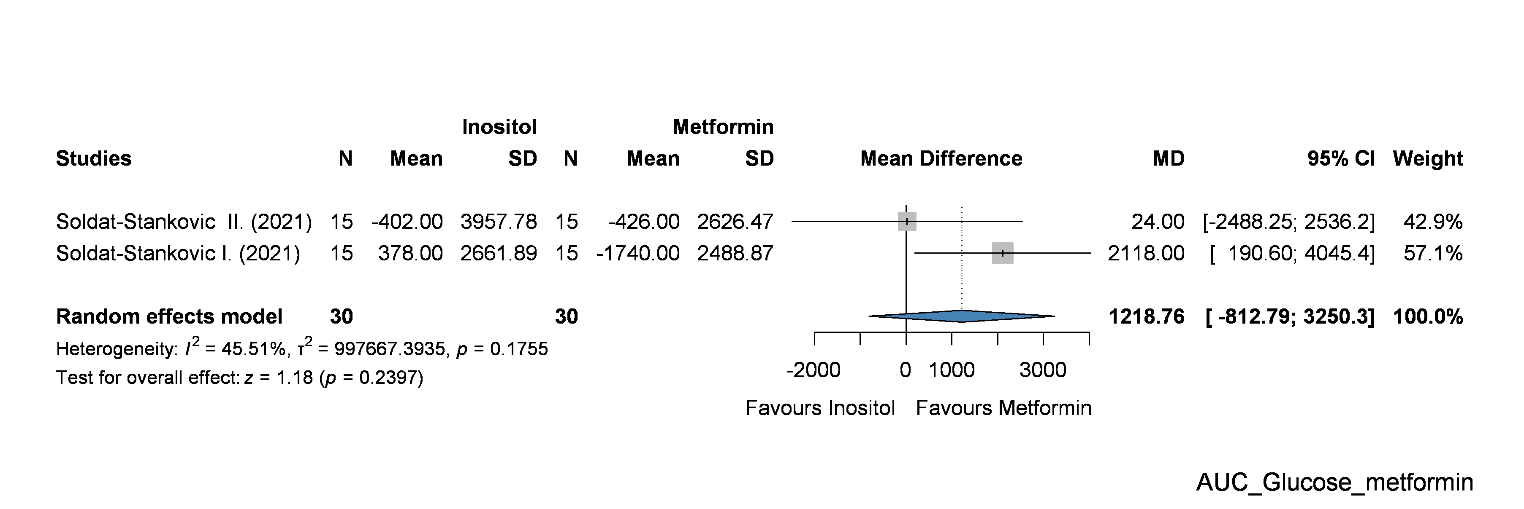


Soldat-Stankovic I. (2021): BMI < 25kg/m^2^ ; Soldat-Stankovic II. (2021): BMI > 25kg/m^2^

**Figure S12c.** Forest plots representing the mean difference of AUC Glucose in the groups treated with inositols compared to placebo or metformin.


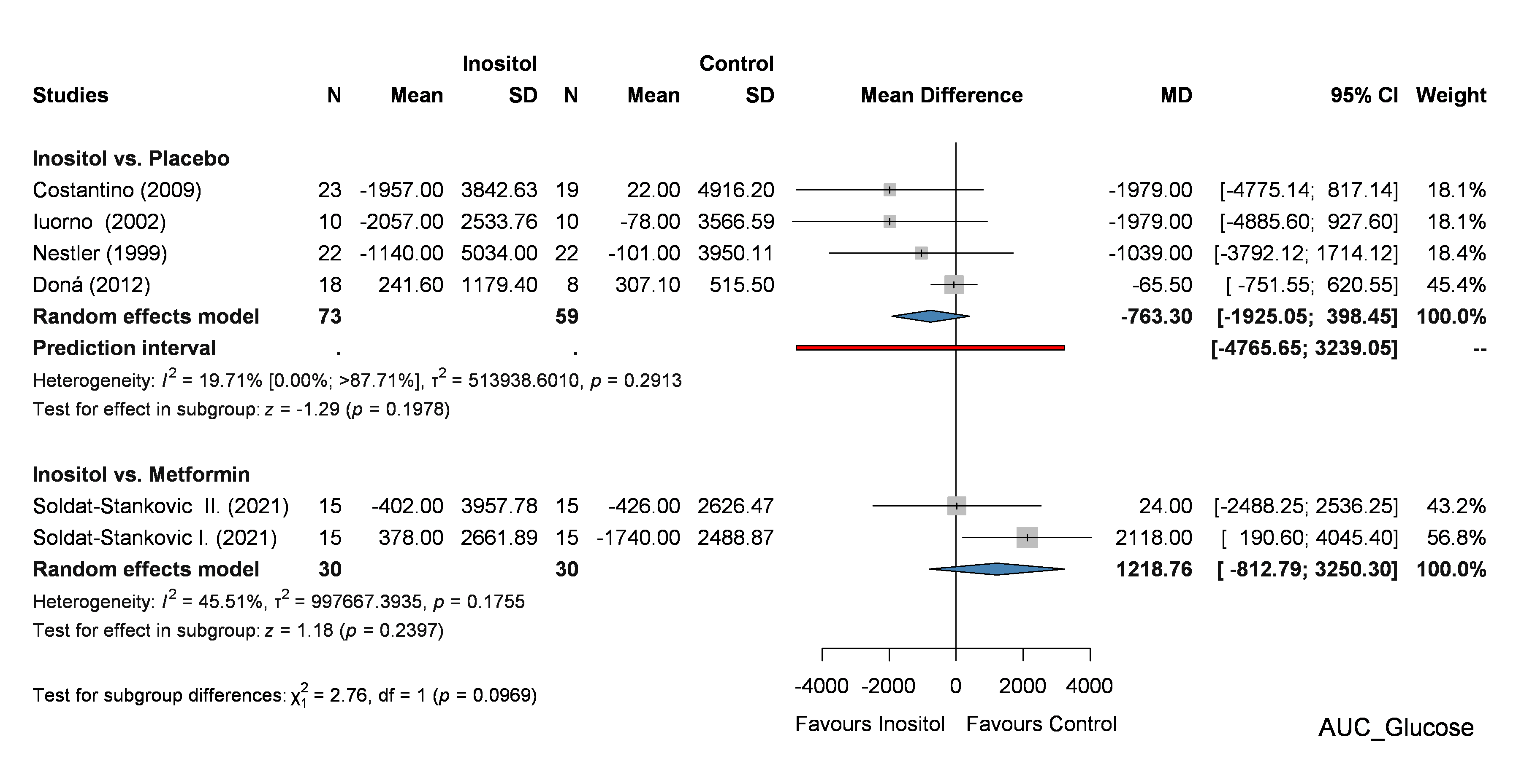


Soldat-Stankovic I. (2021) : BMI < 25kg/m^2^  ; Soldat-Stankovic II. (2021): BMI > 25kg/m^2^

**Figure S13a.** Forest plots representing the mean difference of AUC insulin in the groups treated with different inositol stereoisomers compared to placebo.


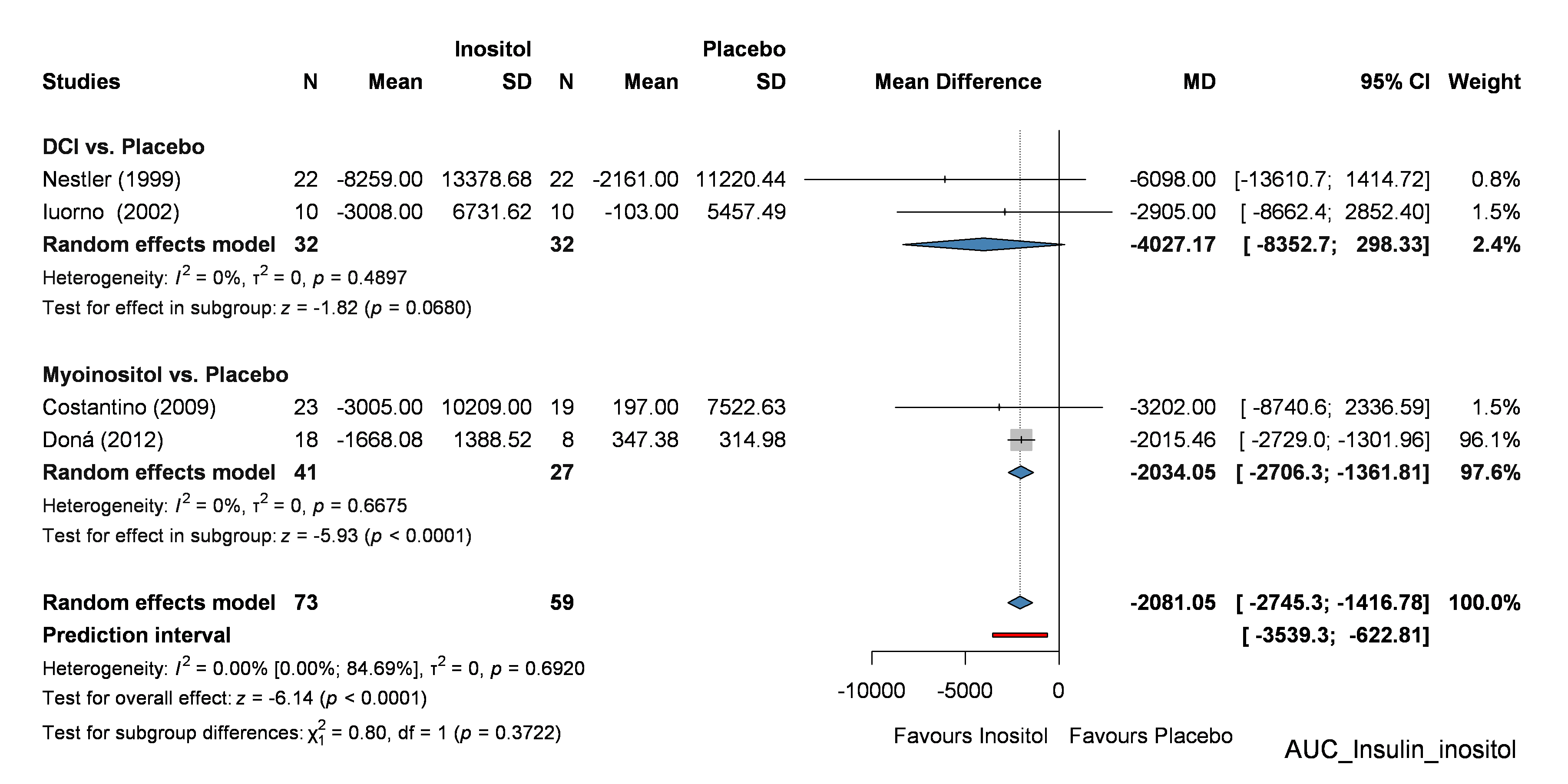


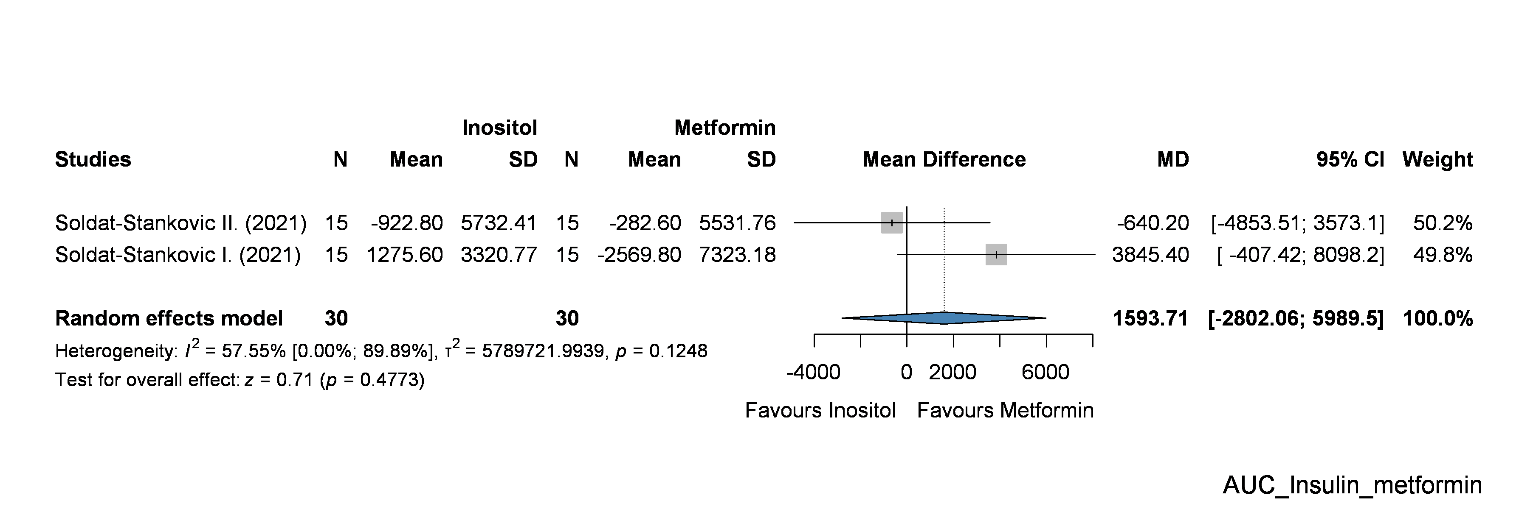
**Figure S13b.** Forest plots representing the mean difference of AUC Insulin in the groups treated with inositols compared to metformin.

Soldat-Stankovic I. (2021) : BMI < 25kg/m^2^  ; Soldat-Stankovic II. (2021): BMI > 25kg/m^2^

**Figure S13c.** Forest plots representing the mean difference of AUC Insulin in the groups treated with inositols compared to placebo or metformin.


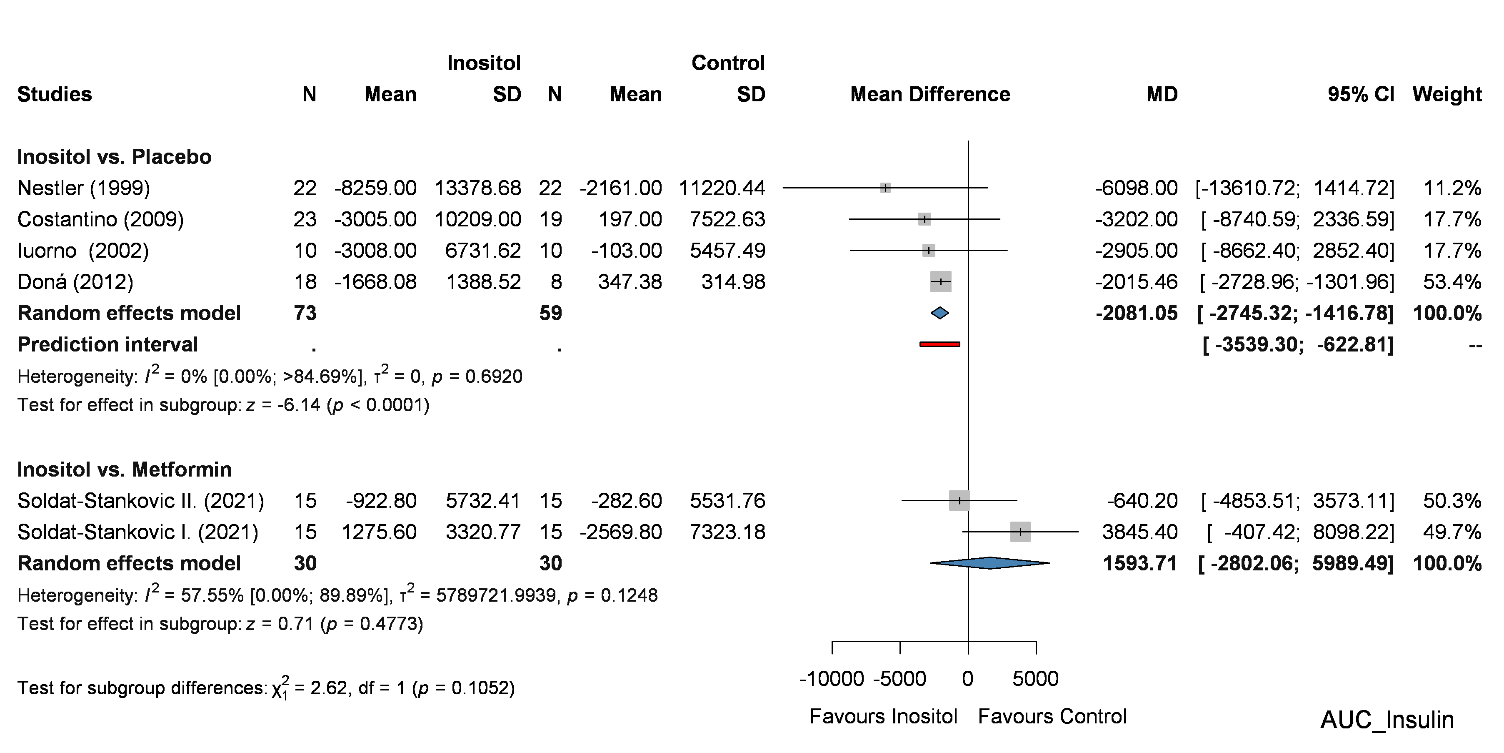


Soldat-Stankovic I. (2021) : BMI < 25kg/m^2^  ; Soldat-Stankovic II. (2021): BMI > 25kg/m^2^

**Figure S13d.** Forest plots representing the mean difference of AUC Insulin in the groups treated with inositols compared to placebo or metformin. (Summary of different inositols into one ‘combined inositols’group).


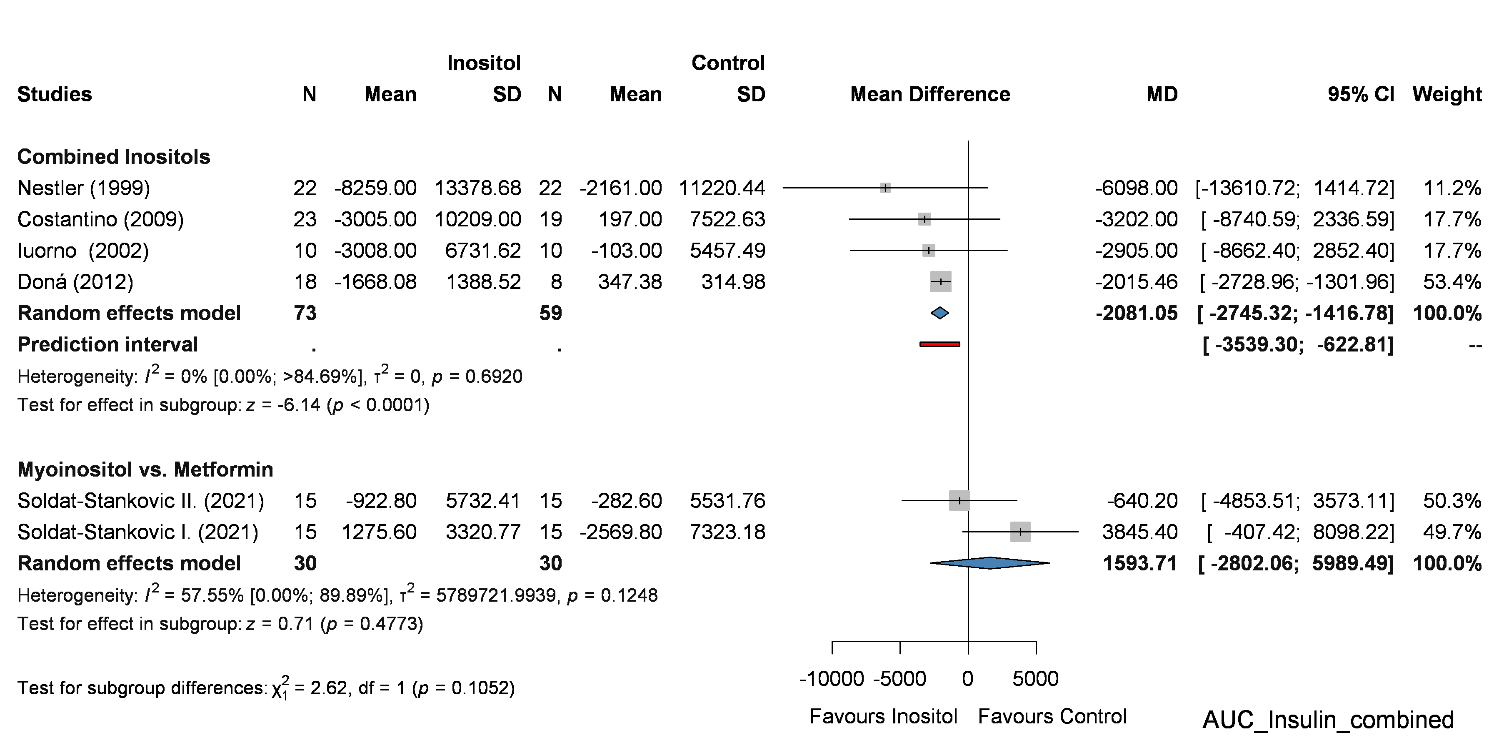


Soldat-Stankovic I. (2021) : BMI < 25kg/m^2^  ; Soldat-Stankovic II. (2021): BMI > 25kg/m^2^

**Figure S14.** Forest plots representing the risk of pregnancy in the groups treated with inositols compared to placebo or metformin (without any other additional treatment).


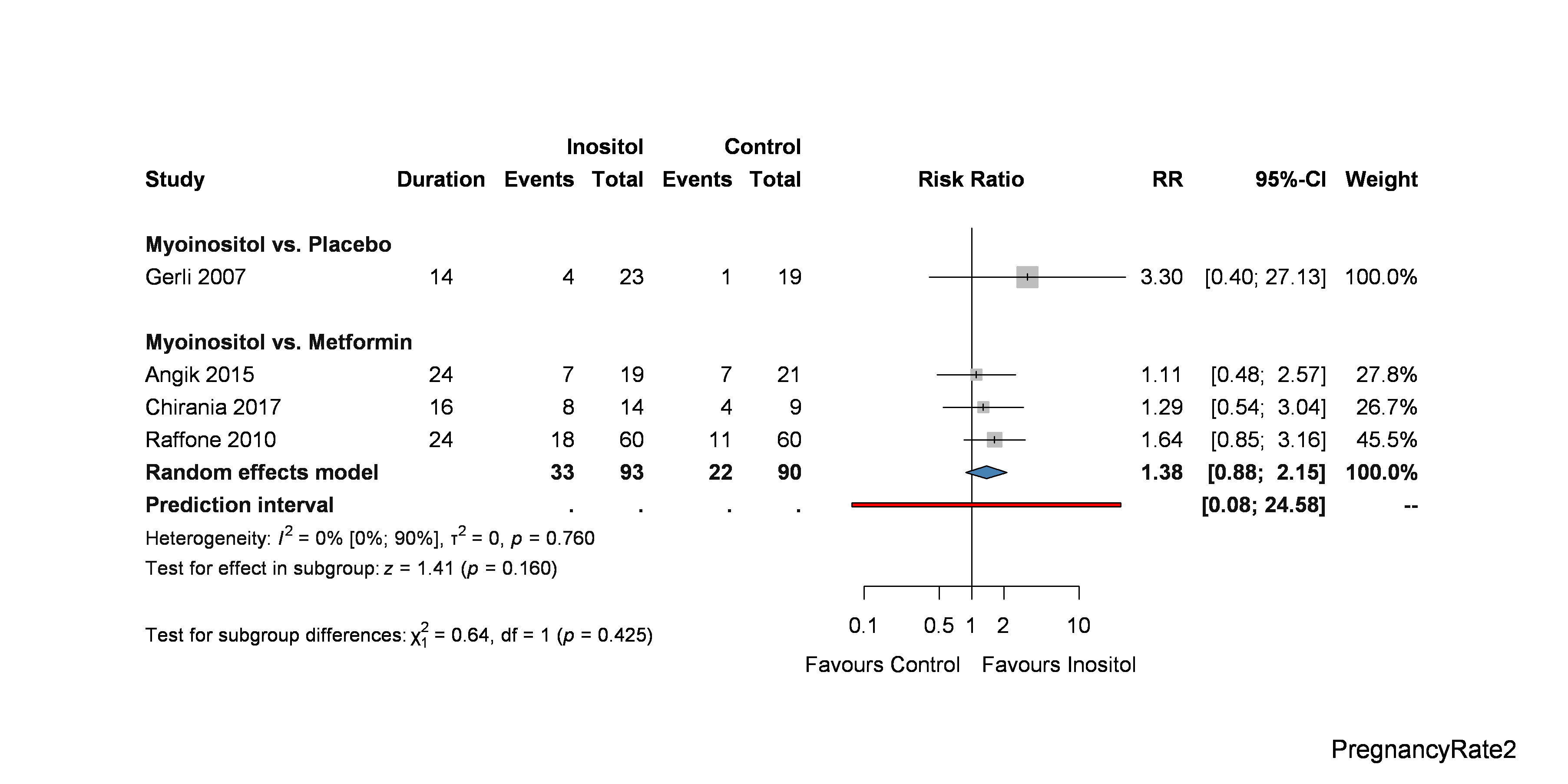


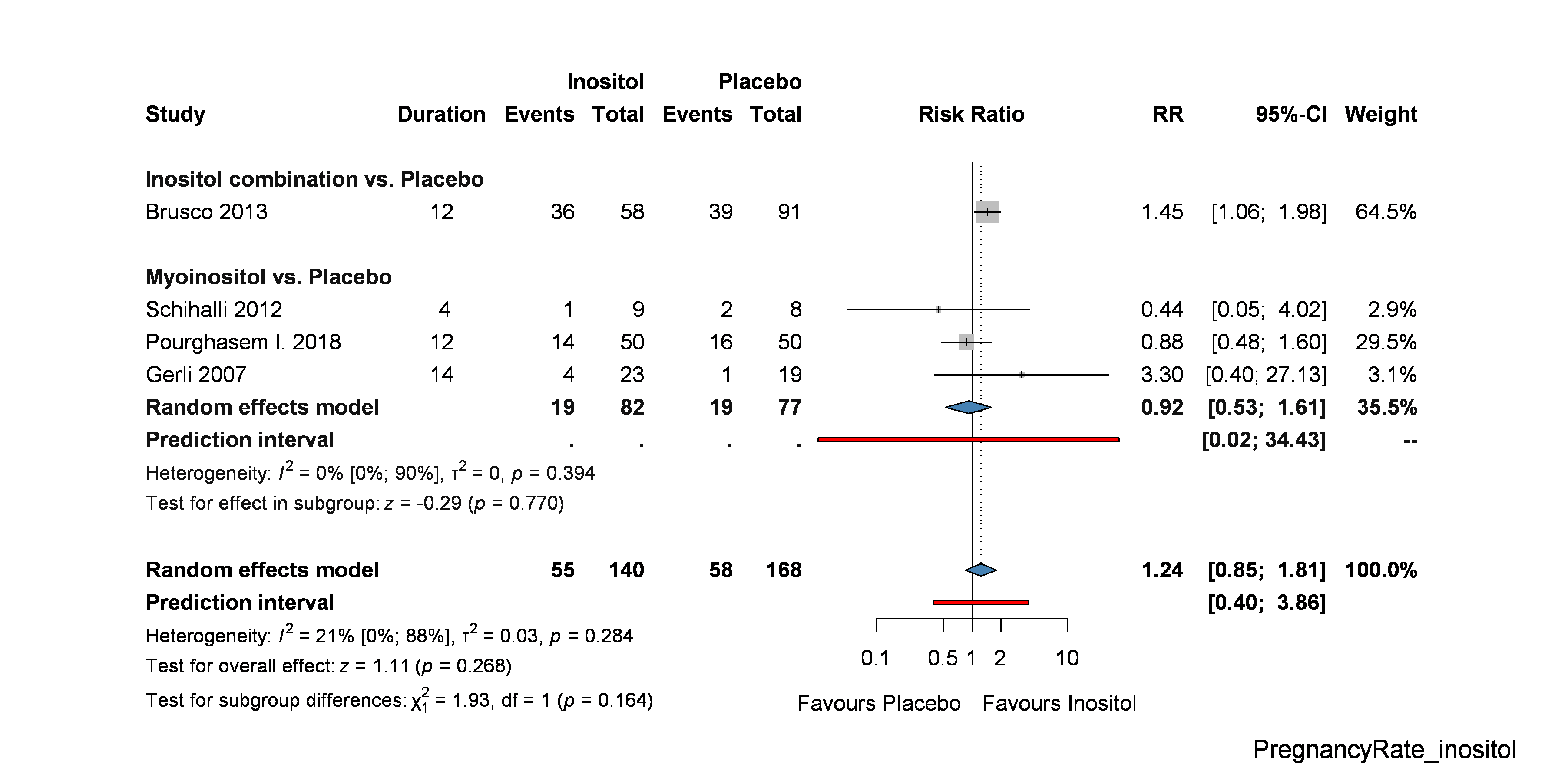
**Figure S15a.** Forest plots representing the risk of pregnancy in the groups treated with different inositol stereoisomers compared to placebo.

Pourghasem I. : Myoinositol and placebo treated group were compared.


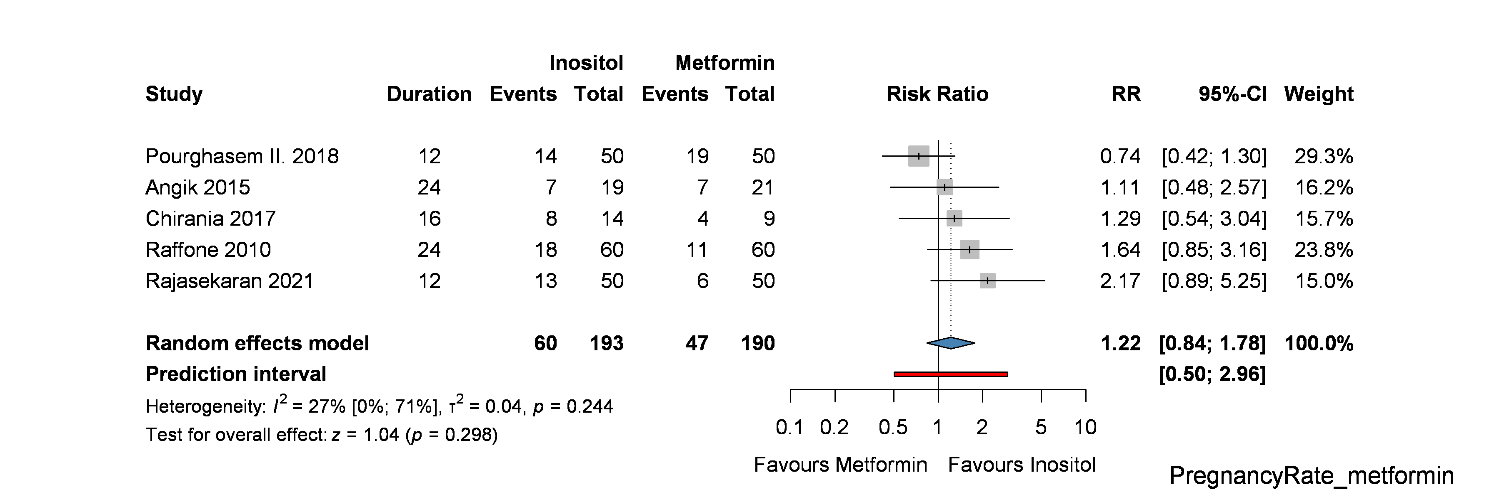
**Figure S15b.** Forest plots representing the risk of pregnancy in the groups treated with inositols compared to metformin.

Pourghasem II. : Myoinositol and metformin treated group were compared.


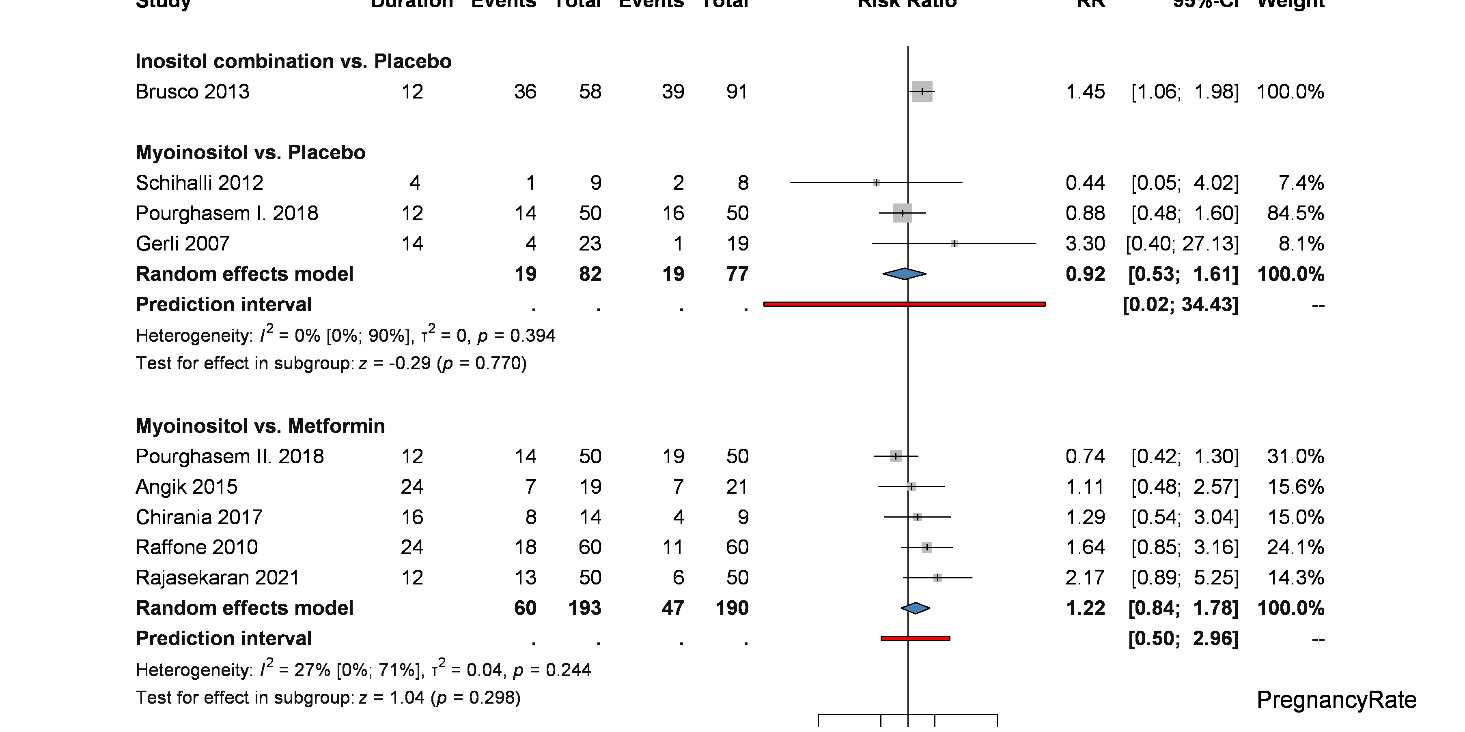
**Figure S15c.** Forest plots representing the risk of pregnancy in the groups treated with inositols compared to placebo or metformin.

Pourhgasem I. (2018) Myoinositol and placebo treated group were compared. Pourhgasem II. (2018) Myoinositol and metformin treated group were compared.

**Figure S15d.** Forest plots representing the risk of pregnancy in the groups treated with inositols compared to placebo or metformin. (Summary of different inositols into one ‘combined inositols’group).


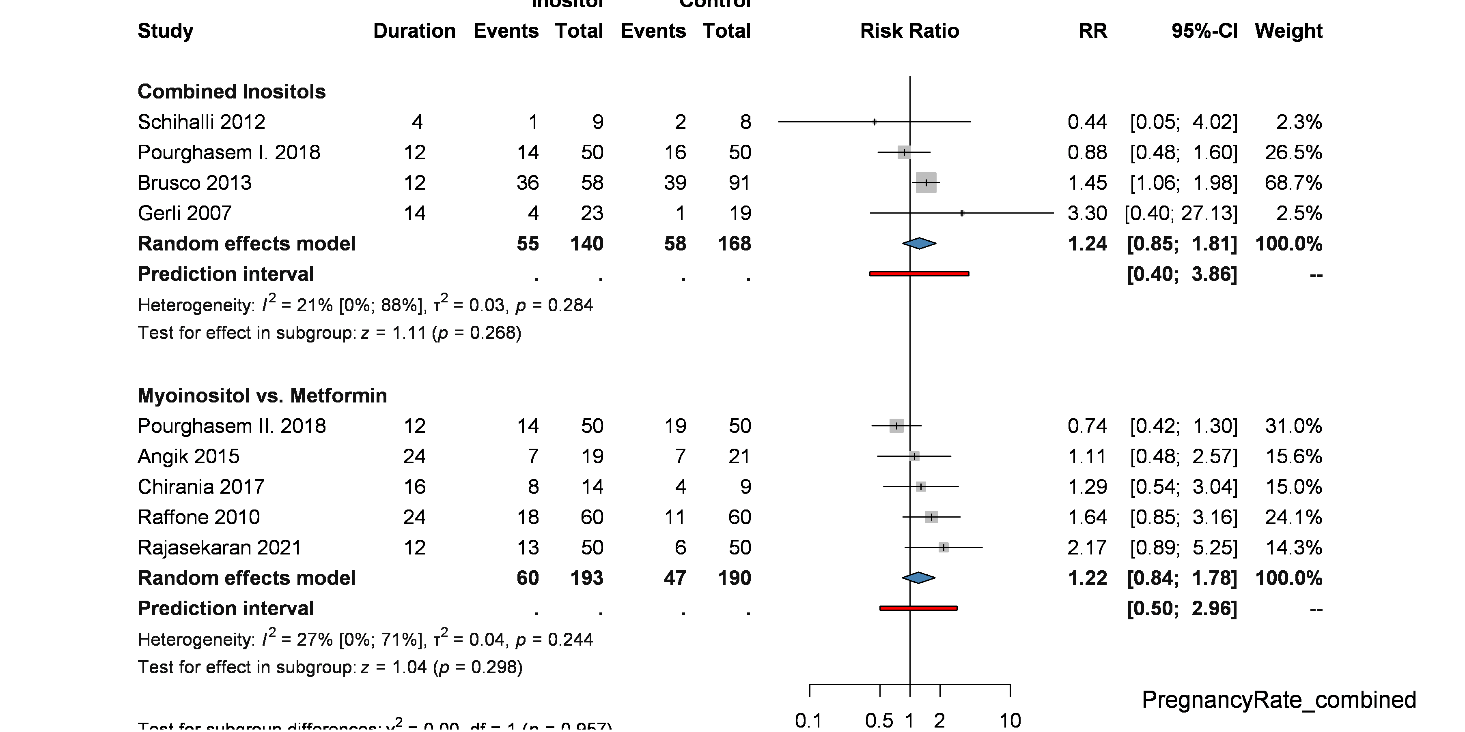


Pourhgasem I. (2018) Myoinositol and placebo treated group were compared. Pourhgasem II. (2018) Myoinositol and metformin treated group were compared.

**Figure S16.** Forest plots representing the risk of side effect in the groups treated with inositols compared to placebo or metformin.


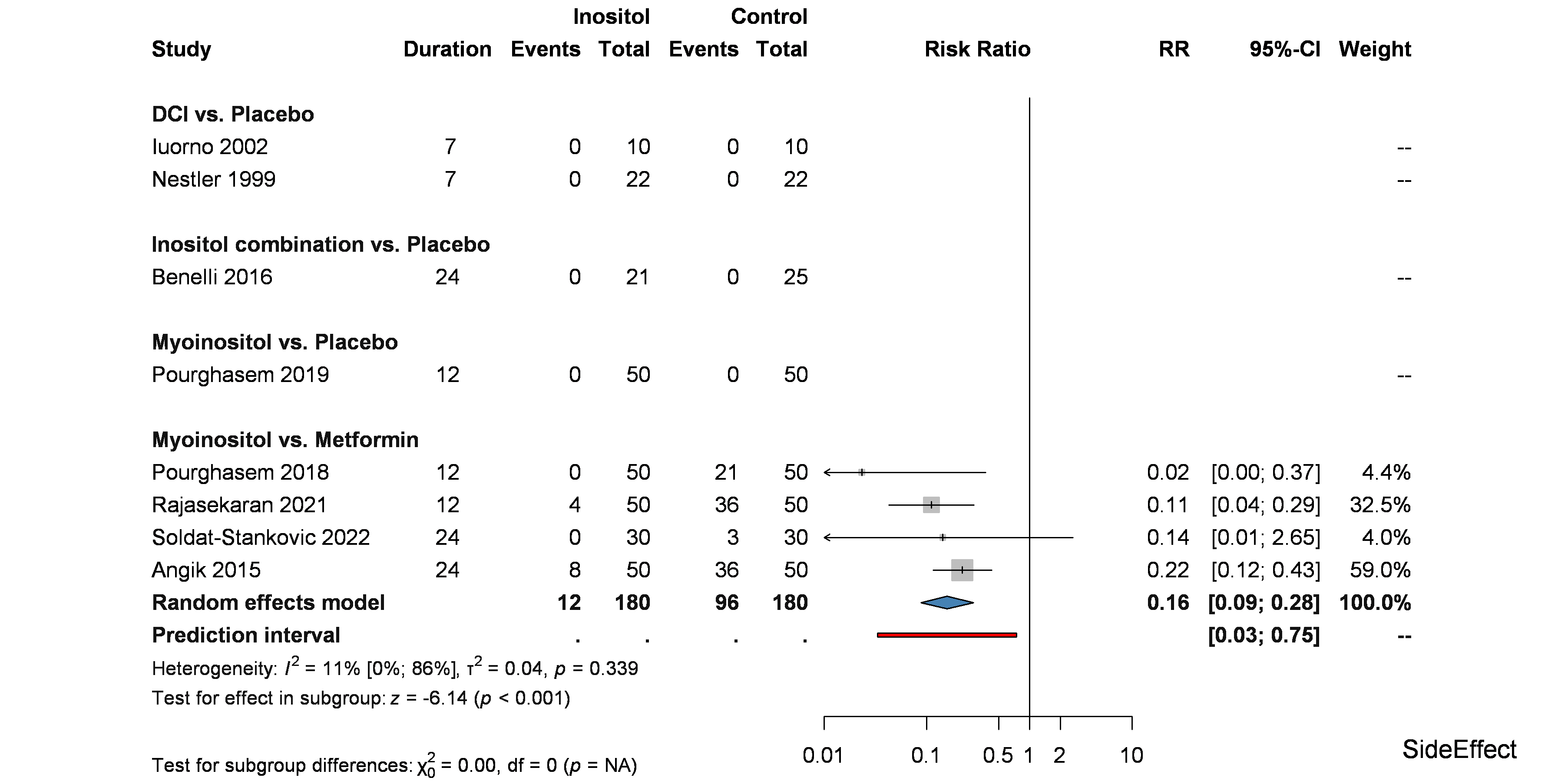


**Figure S17.** Visualisation of ROB2. Comparison of inositol treatment to placebo.


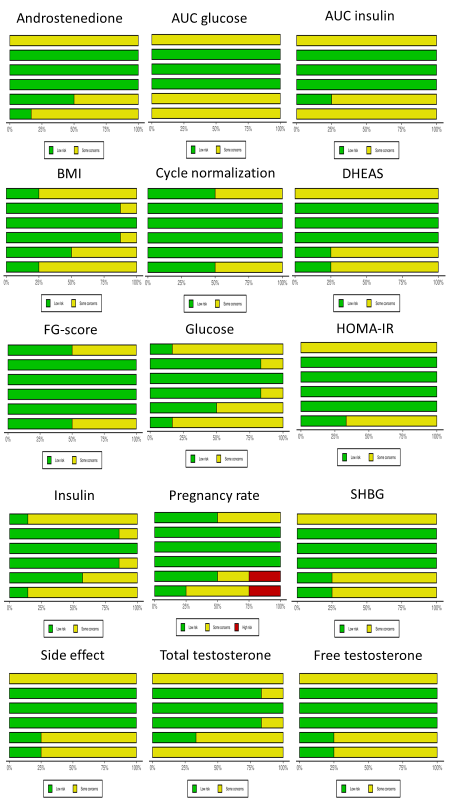


**Figure S18.** Visualisation of ROB2. Comparison of inositol treatment to metformin.


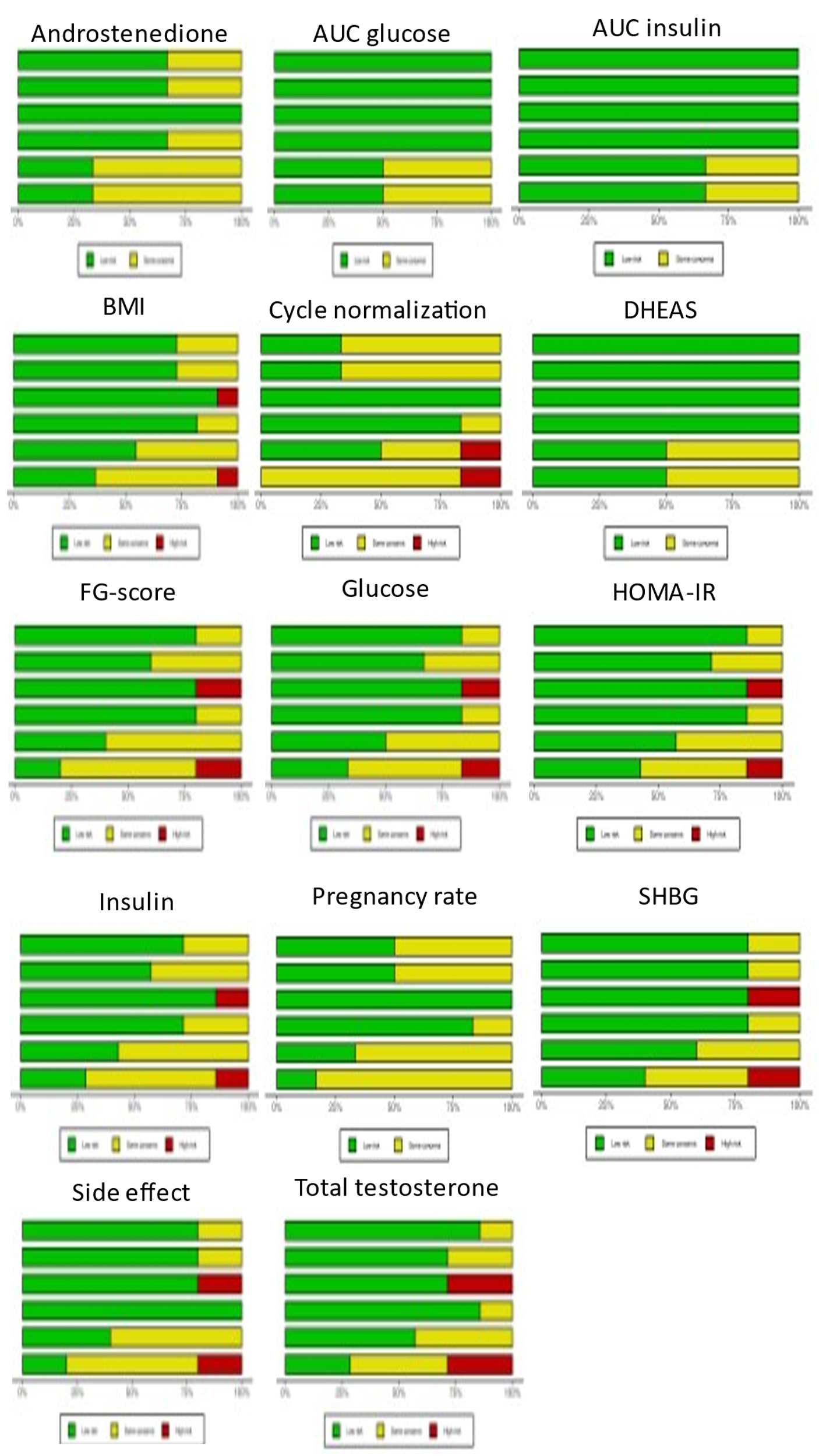


**Table S1.** PRISMA checklist

| **Section and topic** | **Item #** | **Checklist item** | **Location where item is reported** |
| --- | --- | --- | --- |
| **Title** | | | |
| Title | 1 | Identify the report as a systematic review. | 2 |
| **Abstract** | | | |
| Abstract | 2 | See the PRISMA 2020 for Abstracts checklist. |  |
| **Introduction** | | | |
| Rationale | 3 | Describe the rationale for the review in the context of existing knowledge. | 61-91 |
| Objectives | 4 | Provide an explicit statement of the objective(s) or question(s) the review addresses. | 89-91 |
| **Methods** | | | |
| Eligibility criteria | 5 | Specify the inclusion and exclusion criteria for the review and how studies were grouped for the syntheses. | 101-118 |
| Information sources | 6 | Specify all databases, registers, websites, organisations, reference lists and other sources searched or consulted to identify studies. Specify the date when each source was last searched or consulted. | 120-126 |
| Search strategy | 7 | Present the full search strategies for all databases, registers and websites, including any filters and limits used. | 120-126 |
| Selection process | 8 | Specify the methods used to decide whether a study met the inclusion criteria of the review, including how many reviewers screened each record and each report retrieved, whether they worked independently, and if applicable, details of automation tools used in the process. | 127-131 |
| Data collection process | 9 | Specify the methods used to collect data from reports, including how many reviewers collected data from each report, whether they worked independently, any processes for obtaining or confirming data from study investigators, and if applicable, details of automation tools used in the process. | 133-143 |
| Data items | 10a | List and define all outcomes for which data were sought. Specify whether all results that were compatible with each outcome domain in each study were sought (e.g. for all measures, time points, analyses), and if not, the methods used to decide which results to collect. | 133-143 |
|  | 10b | List and define all other variables for which data were sought (e.g. participant and intervention characteristics, funding sources). Describe any assumptions made about any missing or unclear information. | 133-143 |
| Study risk of bias assessment | 11 | Specify the methods used to assess risk of bias in the included studies, including details of the tool(s) used, how many reviewers assessed each study and whether they worked independently, and if applicable, details of automation tools used in the process. | 145-147 |
| Effect measures | 12 | Specify for each outcome the effect measure(s) (e.g. risk ratio, mean difference) used in the synthesis or presentation of results. | 140-143 |
| Synthesis methods | 13a | Describe the processes used to decide which studies were eligible for each synthesis (e.g. tabulating the study intervention characteristics and comparing against the planned groups for each synthesis (item #5)). | 149-168 |
|  | 13b | Describe any methods required to prepare the data for presentation or synthesis, such as handling of missing summary statistics, or data conversions. | 149-168 |
|  | 13c | Describe any methods used to tabulate or visually display results of individual studies and syntheses. | 166 |
|  | 13d | Describe any methods used to synthesise results and provide a rationale for the choice(s). If meta-analysis was performed, describe the model(s), method(s) to identify the presence and extent of statistical heterogeneity, and software package(s) used. | 149-168 |
|  | 13e | Describe any methods used to explore possible causes of heterogeneity among study results (e.g. subgroup analysis, meta-regression). | 149-168 |
|  | 13f | Describe any sensitivity analyses conducted to assess robustness of the synthesised results. |  |
| Reporting bias assessment | 14 | Describe any methods used to assess risk of bias due to missing results in a synthesis (arising from reporting biases). |  |
| Certainty assessment | 15 | Describe any methods used to assess certainty (or confidence) in the body of evidence for an outcome. | 171-172 |
| **Results** | | | |
| Study selection | 16a | Describe the results of the search and selection process, from the number of records identified in the search to the number of studies included in the review, ideally using a flow diagram (see fig 1). | 176-179 |
|  | 16b | Cite studies that might appear to meet the inclusion criteria, but which were excluded, and explain why they were excluded. | 177-178 |
| Study characteristics | 17 | Cite each included study and present its characteristics. | 182-190 |
| Risk of bias in studies | 18 | Present assessments of risk of bias for each included study. | Table S4 |
| Results of individual studies | 19 | For all outcomes, present, for each study: (a) summary statistics for each group (where appropriate) and (b) an effect estimate and its precision (e.g. confidence/credible interval), ideally using structured tables or plots. | Tables S5-9 |
| Results of syntheses | 20a | For each synthesis, briefly summarise the characteristics and risk of bias among contributing studies. | Table S4 |
|  | 20b | Present results of all statistical syntheses conducted. If meta-analysis was done, present for each the summary estimate and its precision (e.g. confidence/credible interval) and measures of statistical heterogeneity. If comparing groups, describe the direction of the effect. | 195-258 |
|  | 20c | Present results of all investigations of possible causes of heterogeneity among study results. |  |
|  | 20d | Present results of all sensitivity analyses conducted to assess the robustness of the synthesised results. |  |
| Reporting biases | 21 | Present assessments of risk of bias due to missing results (arising from reporting biases) for each synthesis assessed. | 260-261 |
| Certainty of evidence | 22 | Present assessments of certainty (or confidence) in the body of evidence for each outcome assessed. | 261-262 |
| **Discussion** | | | |
| Discussion | 23a | Provide a general interpretation of the results in the context of other evidence. | 264-329 |
|  | 23b | Discuss any limitations of the evidence included in the review. | 330-342 |
|  | 23c | Discuss any limitations of the review processes used. | 330-342 |
|  | 23d | Discuss implications of the results for practice, policy, and future research. | 344-347 |
| **Other information** | | | |
| Registration and protocol | 24a | Provide registration information for the review, including register name and registration number, or state that the review was not registered. | 98 |
|  | 24b | Indicate where the review protocol can be accessed, or state that a protocol was not prepared. | 98 |
|  | 24c | Describe and explain any amendments to information provided at registration or in the protocol. | 97-98 |
| Support | 25 | Describe sources of financial or non-financial support for the review, and the role of the funders or sponsors in the review. | 393-397 |
| Competing interests | 26 | Declare any competing interests of review authors. | 391-392 |
| Availability of data, code, and other materials | 27 | Report which of the following are publicly available and where they can be found: template data collection forms; data extracted from included studies; data used for all analyses; analytic code; any other materials used in the review. | 388-390 |

**Table S2.** Eligibility criteria of each included studies

| **Study (year)** | **Inclusion criteria** | **Exclusion criteria** |
| --- | --- | --- |
| Angik, 2015 (1) | **“**All the females of age group 15-40 years attending OPD of Obstetrics and Gynaecology at AVBRH fulfilling the Rotterdam criteria.” | **“-**Patients already on other drug treatment for PCOS (like oral contraceptive pills).  - Deranged kidney or liver function tests  - Thyroid disorders  - Known hypersensitivity to myoinositol „ |
| Benelli, 2016 (2) | “Rotterdam criteria” | “Patients with diabetes, smokers, and alcohol users were ruled out from the study.” |
| Brusco, 2013 (3) | “Women being under 40 years old, at least one previous failed attempt with ICSI with low-quality oocyte recovery, diagnosis of PCOS (i.e., with oligomenorrhea, hyperandrogenism and pelvic ultrasonographic appearance characterized by multiple anechoic areas) diagnosis of “poor responders” (i.e., with poor ovarian response to hormonal stimulation, an age greater than 37 years and the need for high doses of FSH stimulation in previous cycles).“ | “Patients with a partner with a diagnosis of severe male infertility such as cryptozoospermia (i.e., retrieval of sperm in the semen after centrifugation) and azoospermia (i.e., eventual retrieval of sperm from the testicle or epididymis) were excluded from the study.” |
| Chirania, 2017 (4) | „Women presenting in the OBGYN OPD with irregular cycles or oligo/amenorrhea, infertility, hirsutism and excessive acne were evaluated. PCOS was diagnosed based on the rotterdam criteria. Both lean and obese PCOS patients were included in the study.”  „-Patient with oligomenorrhea/amenorrhea  -With polycystic ovaries on USG as described in rotterdam criteria - With or without hyperandrogenism and/or obesity.” | „-Abnormal TSH, PRL, AMH  -Any chronic illness in the past or present- TB, thyroid disease, malabsorption  - History of chemotherapy or radiotherapy in childhood. „ |
| Chhabra, 2018 (5) | “Infertile patients with PCOS as defined by the Rotterdam criteria, namely at least two out of the following three:  1. Menstrual disorders defined as oligomenorrhea (cycle (cycle length >12 weeks)  2. Clinical length >35 days) or amenorrhea hyperandrogenism (modified Ferriman–Gallwey score [mFGS] of ≥6, presence of acne, or seborrhea) and/or biochemical evidence of hyperandrogenemia.  3. Patients with Polycystic ovaries on ultrasound (12 or more measuring 2–9 mm in diameter (mean of both ovaries) and day 2 (D2)/D3 serum AMH levels >5 ng/ml, irrespective of the body mass index (BMI), were included. “ | “1. Women with other causes of PCOs on ultrasound due to anovulation such as hyperprolactinemia, thyroid disorders, late‑onset congenital adrenal hyperplasia, and Cushing’s syndrome by the use of appropriate tests.  2. PCOS patients with AMH <5 ng/ml.  3. Patients with contraindications to use of insulin sensitizers such as metformin due to prior adverse effects.  4. Usage of oral contraceptive and/or any other insulin sensitizer in the previous 3 months.” |
| Costantino, 2009 (6) | “Women 18 to 40 years of age. They were PCOS affected with oligomenorrhea, high serum free testosterone level and/or hirsutism. Women were observed by pelvic, ultrasonography and PCOS was found.” | NR |
| Doná, 2012 (7) | “22-30 years of age with PCOS was defined when two of the following criteria were fulfilled: oligo- and/or anovulation (8 or less menstrual periods in the previous year), clinical and/or biochemical signs of hyperandrogenism, polycystic ovaries and exclusion of other etiologies.” | “Exclusion criteria included pregnancy, body mass index (BMI) >25 kg/m2, hyperprolactinemia, thyroid dysfunction, Cushing’s syndrome, and late-onset adrenal hyperplasia. None of the women had diabetes or had taken oral contraceptives, anti-inflammatory drugs, or other hormonal drugs during the previous 3 months.” |
| Donne, 2019 (8) | “Rotterdam criteria: age between 16 and 45 years; BMI ≥ 25kg/m2 no hormone therapy for less than 6 months; no concurrent medical disease and taking no medications or over-the-counter products at baseline with commitment not to take any throughout the 6-month duration of the study.” | NR |
| Fruzetti, 2016 (9) | “Rotterdam criteria. No subject was using medication known to influence the endocrine and the metabolic profiles. Only subjects affected by insulin resistance and/or hyperinsulinemia were included in the study. No subjects had an abnormal glucose response to the OGTT. All women were affected by acne and/or hirsutism.” | “Subjects with hyperprolactinemia, hypo or hyperthyroidism, congenital adrenal hyperplasia, Cushing’s syndrome or androgen-secreting tumors were excluded from this study” |
| Genazzani, 2008 (10) | 1. “Presence of micropolycystic ovaries at ultrasound 2. Mild to severe hirsutism and/or acne 3. Oligomenorrhea or amenorrhea 4. Absence of enzymatic adrenal deficiency and/or other endocrine disease 5. Normal PRL levels (range 5-25 ng/mL) 6. No hormonal treatment for at least 6 months before the study.” | NR |
| Gerli, 2007 (11) | “oligomenorrhea (cycle length 41d; 8 cycles for year) or amenorrhea and PCOS, aged less than 35 years old, were recruited..” | “Significant hyperprolactinemia, abnormal thyroid function tests, and congenital adrenal hyperplasia.” |
| H.Jamiliam, 2017 (12) | “Rotterdam criteria; 25-65 years of age” | “Smokers, the intake of folate supplements within the last 3 months, pregnant women, individuals with metabolic diseases, thyroid disease, hyperprolactinemia and hypercortisolemia were excluded in the study.” |
| Iuorno, 2002 (13) | “Women 18-40 years. Polycystic ovary syndrome, as defined by the presence of oligomenorrhea (6 menstrual periods during the previous year) and hyperandrogenism (high serum free testosterone levels or hirsutism).” | NR |
| Leo, 2013 (14) | „Patients, aged between 24 and 32 years..”  „All patients met the PCOS criterion of the recent ESHRE/ASRM consensus...” | „..exclusion of other endocrinopathies.” |
| M.Jamiliam, 2017 (15) | “Rotterdam criteria,18-40 years of age.” | “We excluded women who were pregnant during the intervention, and those with adrenal hyperplasia, androgen- secreting tumours, hyperprolactinaemia, thyroid dysfunction, diabetes or impaired glucose tolerance at enrolment.” |
| Nehra J., 2017 (16) | “15-45 y; diagnosed with PCOS according to Androgen Excess Society (AES)/2006 criteria: presence of hyperandrogenism (clinical and/or biochemical), oligo or anovulation, polycystic ovarian morphology (PCOM)-at least one ovary with 12 or more follicles (2-9 mm in diameter) or ovarian volume>10 ml and those willing to give a written informed consent.” | “Women suffering from any neoplastic disease, hyperprolactinemia, Cushing’s disease, hypothyroidism/hyperthyroidism, pregnant and nursing mothers, active liver disease, renal impairment, established type 1 or type 2 diabetes mellitus, any history of drug intake of antidiabetic or estrogen and progesterone, history of any treatment taken in last 3 months, smokers and alcoholic subjects, inability to come for regular follow ups.” |
| Nehra, 2017 (17) | “15-45 y; diagnosed with PCOS according to Androgen Excess Society (AES)/2006 criteria: presence of hyperandrogenism (clinical and/or biochemical), oligo or anovulation, polycystic ovarian morphology (PCOM)-at least one ovary with 12 or more follicles (2-9 mm in diameter) or ovarian volume>10 ml and those willing to give a written informed consent.” | “Women suffering from any neoplastic disease, hyperprolactinemia, Cushing’s disease, hypothyroidism/hyperthyroidism, pregnant and nursing mothers, active liver disease, renal impairment, established type 1 or type 2 diabetes mellitus, any history of drug intake of antidiabetic or estrogen and progesterone, history of any treatment taken in last 3 months, smokers and alcoholic subjects and patients who were unable to come for regular follow-ups. “ |
| Nestler, 1999 (18) | “18 to 40 years of age, with the polycystic ovary syndrome, indicated by the presence of oligomenorrhea (eight or fewer menstrual periods in the previous year) and hyperandrogenism (high serum concentrations of free testosterone or hirsutism). Other inclusion criteria were obesity, defined as a body mass index of more than 28, normal results on thyroid function tests, and normal serum prolactin concentrations.” | NR |
| Pourghasem, 2018 (19) | “Individuals 15–38 years old with PCOS defined according to Rotterdam criteria as having at least two of the following three features: oligo and/or anovulation, hyperandrogenism (clinical and biochemical), and polycystic ovaries on ultrasound scan; inability to get pregnant despite having frequent, unprotected intercourse for at least a year; absence of tubal, anatomic and male factors; intact uterine cavity and normal level of thyroid hormones.” | “The study excluded patients diagnosed with the other endocrine disorders like hyperprolactinemia as well as the patients who have no desire for cooperation.” |
| Raffone, 2010 (20) | “A total of 120 women, aged 535 years, with PCOS, defined by Rotterdam Criteria, were enrolled in the study from June 2006 and June 2008. All patients attended our IVF Department for infertility that lasted for a period of more than 14–16 months.” | „-Other medical condition causing ovulatory dysfunction: hyperprolactinemia or hypothyroidism, or androgen excess, adrenal hyperplasia or Cushing’s syndrome, were excluded by hormonal tests.  -Tubal defects: in fact all women underwent assessment of tubal patency.  -Semen parameters defects: all male partners were evaluated with two different sperm semen samples, without finding any defect.” |
| Rajasekaran, 2021 (21) | “Women with PCOS, as defined by the Rotterdam’s criteria, aged between 21 and 38 years and undergoing their first IVF cycle.” | “Women with conditions unfavorable for implantation (fibroids distorting cavity and thin endometrium), prior ovarian drilling, known diabetes mellitus or previously on hypoglycemic agents and disturbed renal or liver function tests were excluded from the study.” |
| Schihalli,2012 (22) | “In the first group, PCOS has been diagnosed on the basis of Rotterdam/ASRM criteria, in particular according to the presence of at least two of the following symptoms / signs: a) oligomenorrhea (defined as <6 menstrual cycles for year); b) hyperandrogenism signs (hirsutism, acne, alopecia) or hyperandrogenemia (increased total or free T); c) US ovarian morphovolumetry pathognomonic for PCOS.  PCOS group inclusion criteria were:   1. Age 40 or younger 2. Infertility duration ≥2 years 3. Documented anovulation (defined by follicular growth US monitoring and associated progesterone assays for at least 3 months) 4. CC-resistance (defined as no response to CC ≥150 mg from day 3 to day 7 of menstrual cycle, for at least 3 months 5. Negative screening for recurrent pregnancy loss (chromosome mapping, ANA, ENA, APA, thrombophilic screening 6. No other sensitizing or ovarian stimulating therapy for at least 3 months (metformin, ovulation inductors).” | “Excluded hypothyroidism cases and other known hyperandrogenism causes.” |
| Shokrpour, 2021 (23) | “Rotterdam criteria, 18-40 years old.” | “Exclusion criteria were as follows: pregnancy, adrenal hyperplasia, androgen-secreting tumors, hyperprolactinemia, thyroid dysfunction, and diabetes at enrollment.” |
| Singh, 2020 (24) | “Women satisfying “The Rotterdams criteria” for diagnosis of PCOS; no other known causes of infertility; no known metabolic disorder.” | “Women less than 18 years or more than 40 years old; patients on hormonal medication; history of some addiction.” |
| Soldat-Stankovic, 2021 (25) | “Rotterdam criteria, 18-40 years old.” | “Women suffering from thyroid dysfunction, hyperprolactinemia, Cushing syndrome, non-classical congenital adrenal hyperplasia (NCAH), and androgen-secreting tumors were excluded. Women suffering from diabetes, hepatic, renal and cardiovascular disorders, or having a history of alcohol or drug abuse or medical history of breast or uterine cancer were excluded from the study. None of the subjects had received oral contraceptives, glucocorticoids, antiandrogens, and other hormonal agents within the 3 months prior to the initiation of the study.” |
| Tagliaferri, 2017 (26) | “Overweight/obese women (BMI > 25). “  “Rotterdam criteria.” | “The presence of a late-onset adrenal enzyme defect was excluded by an ACTH test. Significant liver or renal impairment, pregnancy and nursing, neoplasm, cardiovascular disease and other hormonal dysfunctions were considered as exclusion criteria.” |

**Table S3.** Intervention and control in each group

| **Study (year)** | **Intervention** | **Control** |
| --- | --- | --- |
| Angik, 2015 (1) | 2g MI; 24w | 1g Metformin; 24w |
| Benelli, 2016 (2) | 1,1g of MI + 27,6 mg of DCI + 400 ug FA*; 24w  *Physiologic ratio of the two isomers is 40:1 | 400 ug FA; 24w |
| Brusco, 2013 (3) | 2g MI+ 400 mg DCI+ 400 ug FA; 12w | 400 mg FA; 12w |
| Chirania, 2017 (4) | 1g MI; 16w | 1g Metformin; 16w |
| Chhabra, 2018 (5) | 4g MI; 12w | 1700 mg Metformin; 12w |
| Costantino, 2009 (6) | 4 g MI + 400 mg FA for 12-16 weeks | 400 mg FA for 12-16 weeks |
| Doná, 2012 (7) | 1200 mg MI + dietary supplement powder; 12w | Placebo powder; 12w |
| Donne, 2019 (8) | 1. 4g MI + 400 ug FA + diet; 24w | Diet; 24w |
|  | 2. 1,1g MI + 400ug FA + 27,6mg DCI+ diet; 24w |  |
| Fruzetti, 2016 (9) | 4 g MI + 400 mg FA; 24w | 1500 mg Metformin; 24w |
| Genazzani, 2008 (10) | 2 g MI + 200 ug FA; 12w | 200 ug FA; 12w |
| Gerli, 2007 (11) | 4 g MI + 400 mg FA; 14w | 400 mg FA; 14w |
| H.Jamiliam, 2017 (12) | 4 g MI + 400 ug FA; 12w | 1500 mg Metformin; 12w |
| Iuorno, 2002 (13) | 600 mg DCI; 6-8 w | Placebo |
| Leo, 2013 (14) | 3g ;24w | 1700mg Metformin;24w |
| M.Jamiliam, 2017 (15) | 4 g MI + 400 ug FA; 12w | 1500 mg Metformin; 12w |
| Nehra J., 2017 (16) | 2g MI; 24w | 1500 mg Metformin; 24w |
| Nehra, 2017 (17) | 2g MI; 24w | 1500 mg Metformin; 24w |
| Nestler, 1999 (18) | 1200 mg DCI; 7 w | Placebo ; 7 w |
| Pourghasem, 2018 (19) | 4g MI + 400 ug FA 12w + 7,5mg letorozol  “7.5 mg letrozole was prescribed daily from the 3^rd^ day of menstruation for 5 days”  “With the observation of at least one mature follicle (≥17 mm),10,000 units of hCG were injected.” | 1. 400 ug FA +7,5 mg letrozol |
|  |  | 1. 3g Metformin + 400 ug FA + 7,5mg letrozole   “7.5 mg letrozole was prescribed daily from the 3^rd^ day of menstruation for 5 days”  “With the observation of at least one mature follicle (≥17 mm),10,000 units of hCG were injected.” |
| Raffone, 2010 (20) | 4g MI+400mcg FA  „until the end of the study (24w)  or a positive pregnancy test.” | 1500mg Metformin  „until the end of the study (24w)  or a positive pregnancy test.” |
| Rajasekaran, 2021 (21) | “4 g MI at least 12 weeks prior to IVF cycle”  “All participating women were advised antagonist protocol: recombinant FSH (77mg/1.75mL) subcutaneously from second day of period or withdrawal bleed”  “GnRH-antagonist, Cetrorelix 0.25 mg/mL subcutaneously until the day of trigger”  “Ovulation trigger given with recombinant hCG (Ovitrelle 250 ug/0.5mL solution for injection in prefilled syringe)”  “GnRH agonist trigger (Injection Lupride 2g – LUPRIDE 0.5 mL (1mg INJ)” | “1700 mg Metformin, at least 12 weeks prior to IVF cycle”  “All participating women were advised antagonist protocol: recombinant FSH (77mg/1.75mL) subcutaneously from second day of period or withdrawal bleed”  “GnRH-antagonist, Cetrorelix 0.25 mg/mL subcutaneously until the day of trigger”  “Ovulation trigger given with recombinant hCG (Ovitrelle 250 ug/0.5mL solution for injection in prefilled syringe)”  “GnRH agonist trigger (Injection Lupride 2g – LUPRIDE 0.5 mL (1mg INJ)” |
| Schihalli,2012 (22) | 4 g MI + 400 ug FA for at least one month before GnRH-agonist administration  “In all patients, timing of stimulation was obtained by inducing a withdrawal bleeding with oral EP or progestin administration 10 mg once a day for seven days. All PCO women enrolled were down-regulated with a GnRH-a (Decapeptyl 0.1 mg *Ipsen*, France; 0.05 mg a day) from mid-luteal phase onwards and, when optimally down-regulated, were stimulated with recombinant FSH with a starting dose of 50-150 IU a day.”  “..in presence of ≤ 2 follicles with a diameter > 16 mm at hCG administration day, cycle was cancelled or converted into Intra Uterine Insemination, on the basis of semen analysis result. Intramuscolar hCG (Gonasi HP 5000 IU, Merck Serono, Switzerland) was administered at a dose of 10,000 IU, and oocyte retrieval was programmed 30 hours later. As a luteal support, vaginal natural progesterone (Progeffik 200 mg, EFFIK ITALIA, Italy) was prescribed to all patients on the day of embryo transfer for 14 days. “ | 400 ug FA; min 4w  “In all patients, timing of stimulation was obtained by inducing a withdrawal bleeding with oral EP or progestin administration 10 mg once a day for seven days. All PCO women enrolled were down-regulated with a GnRH-a (Decapeptyl 0.1 mg *Ipsen*, France; 0.05 mg a day) from mid-luteal phase onwards and, when optimally down-regulated, were stimulated with recombinant FSH with a starting dose of 50-150 IU a day.”  “..in presence of ≤ 2 follicles with a diameter > 16 mm at hCG administration day, cycle was cancelled or converted into Intra Uterine Insemination, on the basis of semen analysis result. Intramuscolar hCG (Gonasi HP 5000 IU, Merck Serono, Switzerland) was administered at a dose of 10,000 IU, and oocyte retrieval was programmed 30 hours later. As a luteal support, vaginal natural progesterone (Progeffik 200 mg, EFFIK ITALIA, Italy) was prescribed to all patients on the day of embryo transfer for 14 days. “ |
| Shokrpour, 2021 (23) | 4g MI + 400 ug FA;12w | 1500 mg Metformin; 12w |
| Singh, 2020 (24) | 4g MI; 12w | 500mcg FA; 12w |
| Soldat-Stankovic, 2021 (25) | 4 g MI + 400 mg FA; 24w | 1500 mg Metformin; 24w |
| Tagliaferri, 2017 (26) | 1 g MI; 24w  *washout interval of 3 months, patients then treated with metformin for 6 months | 1700 mg Metformin; 24w  *washout interval of 3 months, patients then treated with MI for 6 months |

**Table S4.** Risk of bias assessment using the Risk of Bias 2 tool

| **Study ID** | **Experimental** | **Comparator** | **Outcome** | **Randomization process** | **Deviations from intended interventions** | **Missing outcome data** | **Measurement of the outcome** | **Selection of the reported result** | **Overall Bias** |
| --- | --- | --- | --- | --- | --- | --- | --- | --- | --- |
| Fruzetti | Myoinositol | Metformin | Androstenedione | Low | Low | Low | Low | Low | Low |
| Costantino | Myoinositol | Placebo | Androstenedione | Some concerns | Low | Low | Low | Some concerns | Some concerns |
| Genazzani | Myoinositol | Placebo | Androstenedione | Some concerns | Low | Low | Low | Low | Some concerns |
| Tagliaferri | Myoinositol | Metformin | Androstenedione | Low | Low | Low | Low | Some concerns | Some concerns |
| Benelli | Myoinositol+DCI | Placebo | Androstenedione | Some concerns | Low | Low | Low | Low | Low |
| Iuorno | DCI | Placebo | Androstenedione | Some concerns | Low | Low | Low | Some concerns | Some concerns |
| Doná | Myoinositol | Placebo | Androstenedione | Some concerns | Low | Low | Low | Low | Some concerns |
| Nestler | DCI | Placebo | Androstenedione | Some concerns | Low | Low | Low | Some concerns | Some concerns |
| Leo | Myoinositol | Metformin | Androstenedione | Some concerns | Some concerns | Low | Some concerns | Some concerns | Some concerns |
| Costantino | Myoinositol | Placebo | AUC glucose | Some concerns | Low | Low | Low | Some concerns | Some concerns |
| Soldat-Stankovic | Myoinositol | Metformin | AUC Glucose | Low | Low | Low | Low | Low | Low |
| Tagliaferri | Myoinositol | Metformin | AUC Glucose | Low | Low | Low | Low | Some concerns | Some concerns |
| Iuorno | DCI | Placebo | AUC Glucose | Some concerns | Low | Low | Low | Some concerns | Some concerns |
| Nestler | DCI | Placebo | AUC Glucose | Some concerns | Low | Low | Low | Some concerns | Some concerns |
| Fruzetti | Myoinositol | Metformin | AUC Insulin | Low | Low | Low | Low | Low | Low |
| Costantino | Myoinositol | Placebo | AUC insulin | Some concerns | Low | Low | Low | Some concerns | Some concerns |
| Soldat-Stankovic | Myoinositol | Metformin | AUC insulin | Low | Low | Low | Low | Low | Low |
| Tagliaferri | Myoinositol | Metformin | AUC Insulin | Low | Low | Low | Low | Some concerns | Some concerns |
| Iuorno | DCI | Placebo | AUC Insulin | Some concerns | Low | Low | Low | Some concerns | Some concerns |
| Doná | Myoinositol | Placebo | AUC Insulin | Some concerns | Low | Low | Low | Low | Some concerns |
| Nestler | DCI | Placebo | AUC Insulin | Some concerns | Low | Low | Low | Some concerns | Some concerns |
| Fruzetti | Myoinositol | Metformin | BMI | Low | Low | Low | Low | Low | Low |
| Costantino | Myoinositol | Placebo | BMI | Some concerns | Low | Low | Low | Some concerns | Some concerns |
| Genazzani | Myoinositol | Placebo | BMI | Some concerns | Low | Low | Low | Low | Some concerns |
| Gerli,2007 | Myoinositol | Placebo | BMI | Low | Low | Low | Low | Low | Low |
| Hamidreza J. | Myoinositol | Metformin | BMI | Some concerns | Low | Low | Low | Low | Some concerns |
| Mehri Jamilian | Myoinositol | Metformin | BMI | Low | Low | High | Low | Low | High |
| Shokrpour | Myoinositol | Metformin | BMI | Low | Low | Low | Low | Some concerns | Some concerns |
| Soldat-Stankovic | Myoinositol | Metformin | BMI | Low | Low | Low | Low | Low | Low |
| Tagliaferri | Myoinositol | Metformin | BMI | Low | Low | Low | Low | Some concerns | Some concerns |
| Iuorno | DCI | Placebo | BMI | Some concerns | Low | Low | Low | Some concerns | Some concerns |
| Doná | Myoinositol | Placebo | BMI | Some concerns | Low | Low | Low | Low | Some concerns |
| Donne | Myoinositol | Diet | BMI | Low | Low | Low | Low | Low | Low |
| Donne | Myoinositol+DCI | Diet | BMI | Low | Low | Low | Low | Low | Low |
| Nehra J | Myoinositol | Metformin | BMI | Low | Low | Low | Low | Low | Low |
| Nestler | DCI | Placebo | BMI | Some concerns | Low | Low | Low | Some concerns | Some concerns |
| Rajasekaran | Myoinositol | Metformin | BMI | Low | Low | Low | Low | Low | Low |
| Sigh | Myoinositol | Placebo | BMI | Some concerns | Some concerns | Low | Some concerns | Some concerns | Some concerns |
| Angik | Myoinositol | Metformin | BMI | Low | Some concerns | Low | Low | Some concerns | Some concerns |
| Chirania | Myoinositol | Metformin | BMI | Some concerns | Some concerns | Low | Some concerns | Some concerns | Some concerns |
| Leo | Myoinositol | Metformin | BMI | Some concerns | Some concerns | Low | Some concerns | Some concerns | Some concerns |
| Pourghasem | Myoinositol | Metformin | cycle normalisation | Some concerns | Low | Low | Low | Low | Some concerns |
| Pourghasem | Myoinositol | Placebo | cycle normalisation | Some concerns | Low | Low | Low | Low | Some concerns |
| Donne | Myoinositol | Diet | cycle normalisation | Low | Low | Low | Low | Low | Low |
| Donne | Myoinositol+DCI | Diet | cycle normalisation | Low | Low | Low | Low | Low | Low |
| Rajasekaran | Myoinositol | Metformin | cycle normalisation | Low | Low | Low | Low | High | High |
| Chabbra | Myoinositol | Metformin | cycle normalisation | Some concerns | Some concerns | Low | Low | Low | Some concerns |
| Raffone | myoinositol | metformin | cycle normalisation | Some concerns | Some concerns | Low | Low | Low | Some concerns |
| Angik | Myoinositol | Metformin | cycle normalisation | Low | Some concerns | Low | Low | Some concerns | Some concerns |
| Chirania | Myoinositol | Metformin | cycle normalisation | Some concerns | Some concerns | Low | Some concerns | Some concerns | Some concerns |
| Costantino | Myoinositol | Placebo | DHEAS | Some concerns | Low | Low | Low | Some concerns | Some concerns |
| Soldat-Stankovic | Myoinositol | Metformin | DHEAS | Low | Low | Low | Low | Low | Low |
| Tagliaferri | Myoinositol | Metformin | DHEAS | Low | Low | Low | Low | Some concerns | Some concerns |
| Benelli | Myoinositol+DCI | Placebo | DHEAS | Some concerns | Low | Low | Low | Low | Low |
| Iuorno | DCI | Placebo | DHEAS | Some concerns | Low | Low | Low | Some concerns | Some concerns |
| Nestler | DCI | Placebo | DHEAS | Some concerns | Low | Low | Low | Some concerns | Some concerns |
| Genazzani | Myoinositol | Placebo | Ferriman-Gallwey Score | Some concerns | Low | Low | Low | Low | Some concerns |
| Soldat-Stankovic | Myoinositol | Metformin | Ferriman-Gallwey score | Low | Low | Low | Low | Low | Low |
| Tagliaferri | Myoinositol | Metformin | Ferriman-Gallwey Score | Low | Low | Low | Low | Some concerns | Some concerns |
| Donne | Myoinositol | Diet | Ferriman-Gallwey Score | Low | Low | Low | Low | Low | Low |
| Donne | Myoinositol+DCI | Diet | Ferriman-Gallwey Score | Low | Low | Low | Low | Low | Low |
| Leo | Myoinositol | Metformin | Ferriman-Gallwey Score | Some concerns | Some concerns | Low | Some concerns | Some concerns | Some concerns |
| Costantino | Myoinositol | Placebo | Free Testosterone | Some concerns | Low | Low | Low | Some concerns | Some concerns |
| Benelli | Myoinositol+DCI | Placebo | Free testosterone | Some concerns | Low | Low | Low | Low | Low |
| Iuorno | DCI | Placebo | Free Testosterone | Some concerns | Low | Low | Low | Some concerns | Some concerns |
| Nestler | DCI | Placebo | Free Testosterone | Some concerns | Low | Low | Low | Some concerns | Some concerns |
| Leo | Myoinositol | Metformin | Free testosterone | Some concerns | Some concerns | Low | Some concerns | Some concerns | Some concerns |
| Costantino | Myoinositol | Placebo | Glucose | Some concerns | Low | Low | Low | Some concerns | Some concerns |
| Gerli,2007 | Myoinositol | Placebo | Glucose | Low | Low | Low | Low | Low | Some concerns |
| Shokrpour | Myoinositol | Metformin | Glucose | Low | Low | Low | Low | Some concerns | Some concerns |
| Soldat-Stankovic | Myoinositol | Metformin | Glucose | Low | Low | Low | Low | Low | Low |
| Benelli | Myoinositol+DCI | Placebo | Glucose | Some concerns | Low | Low | Low | Low | Low |
| Iuorno | DCI | Placebo | Glucose | Some concerns | Low | Low | Low | Some concerns | Some concerns |
| Doná | Myoinositol | Placebo | Glucose | Some concerns | Low | Low | Low | Low | Some concerns |
| Doná | Myoinositol | Placebo | Glucose | Some concerns | Low | Low | Low | Low | Some concerns |
| Nehra | Myoinositol | Metformin | Glucose | Low | Low | High | Low | Low | High |
| Rajasekaran | Myoinositol | Metformin | Glucose | Low | Low | Low | Low | Low | Low |
| Sigh | Myoinositol | Placebo | Glucose | Some concerns | Some concerns | Low | Some concerns | Some concerns | Some concerns |
| Angik | Myoinositol | Metformin | Glucose | Low | Some concerns | Low | Low | Some concerns | Some concerns |
| Leo | Myoinositol | Metformin | Glucose | Some concerns | Some concerns | Low | Some concerns | Some concerns | Some concerns |
| Genazzani | Myoinositol | Placebo | Glucose/insulin ratio | Some concerns | Low | Low | Low | Low | Some concerns |
| Nehra | Myoinositol | Metformin | Glucose/insulin ratio | Low | Low | High | Low | Low | High |
| Fruzetti | Myoinositol | Metformin | HOMA index | Low | Low | Low | Low | Low | Low |
| Genazzani | Myoinositol | Placebo | HOMA INDEX | Some concerns | Low | Low | Low | Low | Some concerns |
| Shokrpour | Myoinositol | Metformin | HOMA index | Low | Low | Low | Low | Some concerns | Some concerns |
| Soldat-Stankovic | Myoinositol | Metformin | HOMA index | Low | Low | Low | Low | Low | Low |
| Benelli | Myoinositol+DCI | Placebo | HOMA index | Some concerns | Low | Low | Low | Low | Low |
| Doná | Myoinositol | Placebo | HOMA index | Some concerns | Low | Low | Low | Low | Some concerns |
| Nehra | Myoinositol | Metformin | HOMA index | Low | Low | High | Low | Low | High |
| Rajasekaran | Myoinositol | Metformin | HOMA index | Low | Low | Low | Low | Low | Low |
| Angik | Myoinositol | Metformin | HOMA index | Low | Some concerns | Low | Low | Some concerns | Some concerns |
| Leo | Myoinositol | Metformin | HOMA index | Some concerns | Some concerns | Low | Some concerns | Some concerns | Some concerns |
| Costantino | Myoinositol | Placebo | Insulin | Some concerns | Low | Low | Low | Some concerns | Some concerns |
| Genazzani | Myoinositol | Placebo | Insulin | Some concerns | Low | Low | Low | Low | Some concerns |
| Gerli, 2007 | Myoinositol | Placebo | Insulin | Low | Low | Low | Low | Low | Some concerns |
| Shokrpour | Myoinositol | Metformin | Insulin | Low | Low | Low | Low | Some concerns | Some concerns |
| Soldat-Stankovic | Myoinositol | Metformin | Insulin | Low | Low | Low | Low | Low | Low |
| Benelli | Myoinositol+DCI | Placebo | Insulin | Some concerns | Low | Low | Low | Low | Low |
| Iuorno | DCI | Placebo | Insulin | Some concerns | Low | Low | Low | Some concerns | Some concerns |
| Doná | Myoinositol | Placebo | Insulin | Some concerns | Low | Low | Low | Low | Some concerns |
| Nehra | Myoinositol | Metformin | Insulin | Low | Low | High | Low | Low | High |
| Rajasekaran | Myoinositol | Metformin | Insulin | Low | Low | Low | Low | Low | Low |
| Sigh | Myoinositol | Placebo | Insulin | Some concerns | Some concerns | Low | Some concerns | Some concerns | Some concerns |
| Angik | Myoinositol | Metformin | Insulin | Low | Some concerns | Low | Low | Some concerns | Some concerns |
| Chirania | Myoinositol | Metformin | Insulin | Some concerns | Some concerns | Low | Some concerns | Some concerns | Some concerns |
| Leo | Myoinositol | Metformin | Insulin | Some concerns | Some concerns | Low | Some concerns | Some concerns | Some concerns |
| Mehri Jamilian | Myoinositol | Metformin | mFG | Low | Low | High | Low | Low | High |
| Angik | Myoinositol | Metformin | mFG | Low | Some concerns | Low | Low | Some concerns | Some concerns |
| Gerli,2007 | Myoinositol | Placebo | Pregnancy rate | Low | Low | Low | Low | Low | Low |
| Pourghasem | Myoinositol | Metformin | Pregnancy rate | Some concerns | Low | Low | Low | Some concerns | Some concerns |
| Pourghasem | Myoinositol | Placebo | Pregnancy rate | Some concerns | Low | Low | Low | Some concerns | Some concerns |
| Tagliaferri | Myoinositol | Metformin | Pregnancy rate | Low | Low | Low | Low | Some concerns | Some concerns |
| Rajasekaran | Myoinositol | Metformin | Pregnancy rate | Low | Low | Low | Low | Low | Low |
| Schihalli | Myoinositol | Placebo | Pregnancy rate | Some concerns | Low | Low | Low | Low | Some concerns |
| Brusco | Myoinositol | Placebo | Pregnancy rate | Low | Low | Low | Low | High | High |
| Raffone | myoinositol | metformin | Pregnancy rate | Some concerns | Some concerns | Low | Low | Low | Some concerns |
| Angik | Myoinositol | Metformin | Pregnancy rate | Low | Some concerns | Low | Low | Some concerns | Some concerns |
| Chirania | Myoinositol | Metformin | Pregnancy rate | Some concerns | Some concerns | Low | Some concerns | Some concerns | Some concerns |
| Gerli,2007 | Myoinositol | Placebo | Presence of ovulation | Low | Low | Low | Low | Low | Low |
| Nestler | DCI | Placebo | Presence of ovulation | Some concerns | Low | Low | Low | Some concerns | Some concerns |
| Costantino | Myoinositol | Placebo | SHBG | Some concerns | Low | Low | Low | Some concerns | Some concerns |
| Mehri Jamilian | Myoinositol | Metformin | SHBG | Low | Low | High | Low | Low | High |
| Soldat-Stankovic | Myoinositol | Metformin | SHBG | Low | Low | Low | Low | Low | Low |
| Tagliaferri | Myoinositol | Metformin | SHBG | Low | Low | Low | Low | Some concerns | Some concerns |
| Benelli | Myoinositol+DCI | Placebo | SHBG | Some concerns | Low | Low | Low | Low | Low |
| Iuorno | DCI | Placebo | SHBG | Some concerns | Low | Low | Low | Some concerns | Some concerns |
| Nestler | DCI | Placebo | SHBG | Some concerns | Low | Low | Low | Some concerns | Some concerns |
| Rajasekaran | Myoinositol | Metformin | SHBG | Low | Low | Low | Low | Low | Low |
| Leo | Myoinositol | Metformin | SHBG | Some concerns | Some concerns | Low | Some concerns | Some concerns | Some concerns |
| Pourghasem | Myoinositol | Metformin | Side effect | Some concerns | Low | Low | Low | Some concerns | Some concerns |
| Pourghasem | Myoinositol | Placebo | Side effect | Some concerns | Low | Low | Low | Some concerns | Some concerns |
| Soldat-Stankovic | Myoinositol | Metformin | Side effect | Low | Low | High | Low | Low | High |
| Tagliaferri | Myoinositol | Metformin | Side effect | Low | Low | Low | Low | Some concerns | Some concerns |
| Benelli | Myoinositol+DCI | Placebo | Side effect | Some concerns | Low | Low | Low | Low | Low |
| Iuorno | DCI | Placebo | Side effect | Some concerns | Low | Low | Low | Some concerns | Some concerns |
| Nestler | DCI | Placebo | Side effect | Some concerns | Low | Low | Low | Some concerns | Some concerns |
| Rajasekaran | Myoinositol | Metformin | Side effect | Low | Low | Low | Low | Low | Low |
| Angik | Myoinositol | Metformin | Side effect | Low | Some concerns | Low | Low | Some concerns | Some concerns |
| Costantino | Myoinositol | Placebo | Total testosterone | Some concerns | Low | Low | Low | Some concerns | Some concerns |
| Genazzani | Myoinositol | Placebo | Total testosterone | Some concerns | Low | Low | Low | Low | Some concerns |
| Mehri Jamilian | Myoinositol | Metformin | Total testosterone | Low | Low | High | Low | Low | High |
| Soldat-Stankovic | Myoinositol | Metformin | Total Testosterone | Low | Low | Low | Low | Low | Low |
| Tagliaferri | Myoinositol | Metformin | Total Testosterone | Low | Low | Low | Low | Some concerns | Some concerns |
| Iuorno | DCI | Placebo | Total Testosterone | Some concerns | Low | Low | Low | Some concerns | Some concerns |
| Doná | Myoinositol | Placebo | Total Testosterone | Some concerns | Low | Low | Low | Low | Some concerns |
| Nehra | Myoinositol | Metformin | Total testosterone | Low | Low | High | Low | Low | High |
| Nestler | DCI | Placebo | Total Testosterone | Some concerns | Low | Low | Low | Some concerns | Some concerns |
| Rajasekaran | Myoinositol | Metformin | Total Testosterone | Low | Low | Low | Low | Low | Low |
| Sigh | Myoinositol | Placebo | Total Testosterone | Some concerns | Some concerns | Low | Some concerns | Some concerns | Some concerns |
| Angik | Myoinositol | Metformin | Total Testosterone | Low | Some concerns | Low | Low | Some concerns | Some concerns |
| Leo | Myoinositol | Metformin | Total Testosterone | Some concerns | Some concerns | Low | Some concerns | Some concerns | Some concerns |

**Figure S5.**

**Question:** Inositol compared to placebo for the treatment of PCOS

| **Certainty assessment** | | | | | | | **№ of patients** | | **Effect** | | **Certainty** | **Importance** |
| --- | --- | --- | --- | --- | --- | --- | --- | --- | --- | --- | --- | --- |
| **№ of studies** | **Study design** | **Risk of bias** | **Inconsistency** | **Indirectness** | **Imprecision** | **Other considerations** | **inositol** | **placebo** | **Relative (95% CI)** | **Absolute (95% CI)** |  |  |
| **SHBG (follow-up: mean 12 weeks; assessed with: nmol/L)** | | | | | | | | | | | | |
| 4 | randomised trials | not serious | serious^a^ | not serious | serious^b^ | strong association | 76 | 76 | - | **0**  (0 to 0 ) | ⨁⨁⨁◯ Moderate |  |
| **Total testosterone (assessed with: ng/dl)** | | | | | | | | | | | | |
| 6 | randomised trials | not serious | serious | not serious | not serious | none | 149 | 135 | - | **0**  (0 to 0 ) | ⨁⨁⨁◯ Moderate |  |
| **Free testosterone (assessed with: ng/dl)** | | | | | | | | | | | | |
| 4 | randomised trials | serious | serious | not serious | not serious | strong association | 76 | 76 | - | **0**  (0 to 0 ) | ⨁⨁⨁◯ Moderate |  |
| **Androstenedione (assessed with: ng/ml)** | | | | | | | | | | | | |
| 6 | randomised trials | serious | not serious | not serious | not serious | none | 104 | 94 | - | **0**  (0 to 0 ) | ⨁⨁⨁◯ Moderate |  |
| **DHEAS (assessed with: ug/dl)** | | | | | | | | | | | | |
| 4 | randomised trials | not serious | serious | not serious | serious | none | 76 | 76 | - | **0**  (0 to 0 ) | ⨁⨁◯◯ Low |  |
| **Ferriman-Gallwey score** | | | | | | | | | | | | |
| 1 | randomised trials | not serious | not serious | not serious | very serious^b^ | none | 23 | 21 | - | **0**  (0 to 0 ) | ⨁⨁◯◯ Low |  |
| **Glucose (assessed with: mg/dl)** | | | | | | | | | | | | |
| 5 | randomised trials | serious | not serious | not serious | not serious | none | 138 | 128 | - | **0**  (0 to 0 ) | ⨁⨁⨁◯ Moderate |  |
| **Insulin (assessed with: µU/ml)** | | | | | | | | | | | | |
| 6 | randomised trials | serious | serious | not serious | not serious | none | 148 | 138 | - | **0**  (0 to 0 ) | ⨁⨁◯◯ Low |  |
| **HOMA index** | | | | | | | | | | | | |
| 3 | randomised trials | not serious | serious | not serious | serious | none | 49 | 43 | - | **0**  (0 to 0 ) | ⨁⨁◯◯ Low |  |
| **AUC Insulin (assessed with: µU/ml)** | | | | | | | | | | | | |
| 4 | randomised trials | not serious | not serious | not serious | not serious | none | 73 | 59 | - | **0**  (0 to 0 ) | ⨁⨁⨁⨁ High |  |
| **AUC glucose (assessed with: mg/dl)** | | | | | | | | | | | | |
| 4 | randomised trials | not serious | not serious | not serious | serious | none | 73 | 59 | - | **0**  (0 to 0 ) | ⨁⨁⨁◯ Moderate |  |
| **BMI (assessed with: kg/m2)** | | | | | | | | | | | | |
| 8 | randomised trials | not serious | not serious | not serious | not serious | none | 216 | 203 | - | **0**  (0 to 0 ) | ⨁⨁⨁⨁ High |  |
| **cycle normalisation** | | | | | | | | | | | | |
| 2 | randomised trials | serious | not serious | not serious | very serious | publication bias strongly suspected | 35/65 (53.8%) | 16/53 (30.2%) | **RR 1.79** (1.13 to 2.85) | **238 more per 1 000** (from 39 more to 558 more) | ⨁◯◯◯ Very low |  |
| **pregnancy rate** | | | | | | | | | | | | |
| 4 | randomised trials | serious | not serious | not serious | very serious | publication bias strongly suspected | 55/140 (39.3%) | 58/168 (34.5%) | **RR 1.24** (0.85 to 1.81) | **83 more per 1 000** (from 52 fewer to 280 more) | ⨁◯◯◯ Very low |  |
| **pregnancy rate 2** | | | | | | | | | | | | |
| 1 | randomised trials | not serious | not serious | not serious | very serious | none | 4/14 (28.6%) | 1/23 (4.3%) | **RR 3.30** (0.40 to 27.13) | **100 more per 1 000** (from 26 fewer to 1 000 more) | ⨁⨁◯◯ Low |  |

**Table S6.** Myoinositol compared to placebo for the treatment of PCOS

| **Certainty assessment** | | | | | | | **№ of patients** | | **Effect** | | **Certainty** | **Importance** |
| --- | --- | --- | --- | --- | --- | --- | --- | --- | --- | --- | --- | --- |
| **№ of studies** | **Study design** | **Risk of bias** | **Inconsistency** | **Indirectness** | **Imprecision** | **Other considerations** | **myoinositol** | **placebo** | **Relative (95% CI)** | **Absolute (95% CI)** |  |  |
| **Total testosterone (assessed with: ng/dl)** | | | | | | | | | | | | |
| 4 | randomised trials | not serious | serious | not serious | not serious | none | 117 | 103 | - | **0**  (0 to 0 ) | ⨁⨁⨁◯ Moderate |  |
| **Androstenedione (assessed with: ng/ml)** | | | | | | | | | | | | |
| 3 | randomised trials | not serious | serious | not serious | very serious | none | 51 | 37 | - | **0**  (0 to 0 ) | ⨁◯◯◯ Very low |  |
| **SHBG (assessed with: nmol/L)** | | | | | | | | | | | | |
| 1 | randomised trials | not serious | not serious | not serious | serious | none | 23 | 19 | - | **0**  (0 to 0 ) | ⨁⨁⨁◯ Moderate |  |
| **DHEAS (assessed with: µg/dl)** | | | | | | | | | | | | |
| 1 | randomised trials | not serious | not serious | not serious | serious | strong association | 23 | 19 | - | **0**  (0 to 0 ) | ⨁⨁⨁⨁ High |  |
| **AUC Glucose (assessed with: mg/dl/min)** | | | | | | | | | | | | |
| 2 | randomised trials | not serious | serious | not serious | serious | none | 41 | 27 | - | **0**  (0 to 0 ) | ⨁⨁◯◯ Low |  |
| **AUC Insulin (assessed with: µg/ml/min)** | | | | | | | | | | | | |
| 2 | randomised trials | not serious | not serious | not serious | not serious | none | 41 | 27 | - | **0**  (0 to 0 ) | ⨁⨁⨁⨁ High |  |
| **BMI (assessed with: kg/m)** | | | | | | | | | | | | |
| 5 | randomised trials | not serious | not serious | not serious | not serious | none | 106 | 105 | - | **0**  (0 to 0 ) | ⨁⨁⨁⨁ High |  |
| **Pregnancy rate** | | | | | | | | | | | | |
| 3 | randomised trials | serious | not serious | not serious | extremely serious | none | 19/82 (23.2%) | 19/77 (24.7%) | **RR 0.92** (0.53 to 1.61) | **20 fewer per 1 000** (from 116 fewer to 151 more) | ⨁◯◯◯ Very low |  |
| **Free testosterone (assessed with: mg/dl)** | | | | | | | | | | | | |
| 1 | randomised trials | not serious | not serious | not serious | serious | strong association | 23 | 19 | - | **0**  (0 to 0 ) | ⨁⨁⨁⨁ High |  |
| **Glucose (assessed with: mg/dl)** | | | | | | | | | | | | |
| 3 | randomised trials | serious | not serious | not serious | not serious | none | 107 | 93 | - | **0**  (0 to 0 ) | ⨁⨁⨁◯ Moderate |  |
| **Insulin (assessed with: uU/ml)** | | | | | | | | | | | | |
| 4 | randomised trials | serious | serious | not serious | not serious | none | 117 | 103 | - | **0**  (0 to 0 ) | ⨁⨁◯◯ Low |  |
| **HOMA index** | | | | | | | | | | | | |
| 2 | randomised trials | not serious | serious | not serious | not serious | none | 28 | 18 | - | **0**  (0 to 0 ) | ⨁⨁⨁◯ Moderate |  |
| **Pregnancy rate 2** | | | | | | | | | | | | |
| 1 | randomised trials | serious | not serious | not serious | very serious | none | 4/23 (17.4%) | 1/19 (5.3%) | **RR 3.30** (0.40 to 27.13) | **121 more per 1 000** (from 32 fewer to 1 000 more) | ⨁◯◯◯ Very low |  |
| **cycle normalisation** | | | | | | | | | | | | |
| 1 | randomised trials | not serious | not serious | not serious | serious | none | 27/46 (58.7%) | 16/53 (30.2%) | **RR 1.76** (1.06 to 2.92) | **229 more per 1 000** (from 18 more to 580 more) | ⨁⨁⨁◯ Moderate |  |

**Table S7.** DCI compared to placebo for the treatment of PCOS

| **Certainty assessment** | | | | | | | **№ of patients** | | **Effect** | | **Certainty** | **Importance** |
| --- | --- | --- | --- | --- | --- | --- | --- | --- | --- | --- | --- | --- |
| **№ of studies** | **Study design** | **Risk of bias** | **Inconsistency** | **Indirectness** | **Imprecision** | **Other considerations** | **DCI** | **placebo** | **Relative (95% CI)** | **Absolute (95% CI)** |  |  |
| **SHBG (assessed with: nmol/L)** | | | | | | | | | | | | |
| 2 | randomised trials | serious | not serious | not serious | not serious | strong association | 32 | 32 | - | **0**  (0 to 0 ) | ⨁⨁⨁⨁ High |  |
| **Total testosterone (assessed with: ng/dl)** | | | | | | | | | | | | |
| 2 | randomised trials | not serious | not serious | not serious | serious^a^ | strong association | 32 | 32 | - | **0**  (0 to 0 ) | ⨁⨁⨁⨁ High |  |
| **Free testosterone (assessed with: ng/dl)** | | | | | | | | | | | | |
| 2 | randomised trials | not serious | not serious | not serious | not serious | strong association | 32 | 32 | - | **0**  (0 to 0 ) | ⨁⨁⨁⨁ High |  |
| **Androstenedione (assessed with: ng/ml)** | | | | | | | | | | | | |
| 2 | randomised trials | not serious | not serious | not serious | very serious^b^ | none | 32 | 32 | - | **0**  (0 to 0 ) | ⨁⨁◯◯ Low |  |
| **DHEAS (assessed with: ug/dl)** | | | | | | | | | | | | |
| 2 | randomised trials | not serious | not serious | not serious | very serious^b^ | strong association | 32 | 32 | - | **0**  (0 to 0 ) | ⨁⨁⨁◯ Moderate |  |
| **Glucose (assessed with: mg/dl)** | | | | | | | | | | | | |
| 1 | randomised trials | not serious | not serious | not serious | very serious^b^ | none | 10 | 10 | - | **0**  (0 to 0 ) | ⨁⨁◯◯ Low |  |
| **Insulin (assessed with: uU/ml)** | | | | | | | | | | | | |
| 1 | randomised trials | not serious | not serious | not serious | very serious^b^ | none | 10 | 10 | - | **0**  (0 to 0 ) | ⨁⨁◯◯ Low |  |
| **BMI (assessed with: kg/m2)** | | | | | | | | | | | | |
| 2 | randomised trials | not serious | not serious | not serious | very serious^b^ | none | 32 | 32 | - | **0**  (0 to 0 ) | ⨁⨁◯◯ Low |  |
| **AUC Glucose (assessed with: mg/dl/min)** | | | | | | | | | | | | |
| 2 | randomised trials | not serious | not serious | not serious | very serious | none | 32 | 32 | - | **0**  (0 to 0 ) | ⨁⨁◯◯ Low |  |
| **AUC insulin (assessed with: µU/ml/min)** | | | | | | | | | | | | |
| 2 | randomised trials | not serious | not serious | not serious | serious | none | 32 | 32 | - | **0**  (0 to 0 ) | ⨁⨁⨁◯ Moderate |  |

**Table S8.** Inositol combination (DCI+MYO) compared to placebo for the treatment of PCOS

| **Certainty assessment** | | | | | | | **№ of patients** | | **Effect** | | **Certainty** | **Importance** |
| --- | --- | --- | --- | --- | --- | --- | --- | --- | --- | --- | --- | --- |
| **№ of studies** | **Study design** | **Risk of bias** | **Inconsistency** | **Indirectness** | **Imprecision** | **Other considerations** | **inositol combination (DCI+MYO)** | **placebo** | **Relative (95% CI)** | **Absolute (95% CI)** |  |  |
| **SHBG (assessed with: nmol/L)** | | | | | | | | | | | | |
| 1 | randomised trials | not serious | not serious | not serious | serious^a^ | none | 21 | 25 | - | **0**  (0 to 0 ) | ⨁⨁⨁◯ Moderate |  |
| **Free testosterone (assessed with: ng/dl)** | | | | | | | | | | | | |
| 1 | randomised trials | not serious | not serious | not serious | very serious^b^ | none | 21 | 25 | - | **0**  (0 to 0 ) | ⨁⨁◯◯ Low |  |
| **Androstenedione (assessed with: ng/ml)** | | | | | | | | | | | | |
| 1 | randomised trials | not serious | not serious | not serious | very serious^b^ | none | 21 | 25 | - | **0**  (0 to 0 ) | ⨁⨁◯◯ Low |  |
| **DHEAS (assessed with: ug/dl)** | | | | | | | | | | | | |
| 1 | randomised trials | not serious | not serious | not serious | very serious^b^ | none | 21 | 25 | - | **0**  (0 to 0 ) | ⨁⨁◯◯ Low |  |
| **Ferriman-Gallwey score** | | | | | | | | | | | | |
| 1 | randomised trials | not serious | not serious | not serious | very serious^b^ | none | 22 | 21 | - | **0**  (0 to 0 ) | ⨁⨁◯◯ Low |  |
| **Glucose (assessed with: mg/dl)** | | | | | | | | | | | | |
| 1 | randomised trials | not serious | not serious | not serious | very serious^b^ | none | 21 | 25 | - | **0**  (0 to 0 ) | ⨁⨁◯◯ Low |  |
| **Insulin (assessed with: uU/ml)** | | | | | | | | | | | | |
| 1 | randomised trials | not serious | not serious | not serious | serious^c^ | strong association | 21 | 25 | - | **0**  (0 to 0 ) | ⨁⨁⨁⨁ High |  |
| **HOMA Index** | | | | | | | | | | | | |
| 1 | randomised trials | not serious | not serious | not serious | very serious^b^ | none | 21 | 25 | - | **0**  (0 to 0 ) | ⨁⨁◯◯ Low |  |
| **BMI (assessed with: kg/m2)** | | | | | | | | | | | | |
| 1 | randomised trials | not serious | not serious | not serious | very serious^b^ | none | 22 | 21 | - | **0**  (0 to 0 ) | ⨁⨁◯◯ Low |  |
| **Cycle Normalisation** | | | | | | | | | | | | |
| 1 | randomised trials | not serious | not serious | not serious | very serious^b^ | none | 8/19 (42.1%) | 3/14 (21.4%) | **RR 1.96** (0.63 to 6.10) | **206 more per 1 000** (from 79 fewer to 1 000 more) | ⨁⨁◯◯ Low |  |
| **pregnancy rate** | | | | | | | | | | | | |
| 1 | randomised trials | serious | not serious | not serious | serious^c^ | none | 36/58 (62.1%) | 39/91 (42.9%) | **RR 1.45** (1.06 to 1.98) | **193 more per 1 000** (from 26 more to 420 more) | ⨁⨁◯◯ Low |  |

**Table S9.** Inositol compared to metformin for the treatment of the symptoms of PCOS

| **Certainty assessment** | | | | | | | **№ of patients** | | **Effect** | | **Certainty** | **Importance** |
| --- | --- | --- | --- | --- | --- | --- | --- | --- | --- | --- | --- | --- |
| **№ of studies** | **Study design** | **Risk of bias** | **Inconsistency** | **Indirectness** | **Imprecision** | **Other considerations** | **inositol** | **metformin** | **Relative (95% CI)** | **Absolute (95% CI)** |  |  |
| **SHBG (assessed with: nmol/l)** | | | | | | | | | | | | |
| 3 | randomised trials | serious | not serious | not serious | not serious | none | 110 | 110 | - | **0**  (0 to 0 ) | ⨁⨁⨁◯ Moderate |  |
| **Total testosterone (assessed with: ng/dl)** | | | | | | | | | | | | |
| 4 | randomised trials | serious | not serious | not serious | not serious | none | 160 | 160 | - | **0**  (0 to 0 ) | ⨁⨁⨁◯ Moderate |  |
| **Ferriman-Gallwey score** | | | | | | | | | | | | |
| 3 | randomised trials | not serious | not serious | not serious | not serious | none | 110 | 110 | - | **0**  (0 to 0 ) | ⨁⨁⨁⨁ High |  |
| **DHEAS (assessed with: µg/dl)** | | | | | | | | | | | | |
| 1 | randomised trials | not serious | not serious | not serious | very serious | none | 30 | 30 | - | **0**  (0 to 0 ) | ⨁⨁◯◯ Low |  |
| **AUC Glucose (assessed with: mg/dl/min)** | | | | | | | | | | | | |
| 1 | randomised trials | not serious | not serious | not serious | serious | none | 30 | 30 | - | **0**  (0 to 0 ) | ⨁⨁⨁◯ Moderate |  |
| **AUC insulin (assessed with: µU/ml/min)** | | | | | | | | | | | | |
| 1 | randomised trials | not serious | not serious | not serious | serious | none | 30 | 30 | - | **0**  (0 to 0 ) | ⨁⨁⨁◯ Moderate |  |
| **BMI (assessed with: kg/m2)** | | | | | | | | | | | | |
| 9 | randomised trials | not serious | not serious | not serious | not serious | none | 296 | 297 | - | **0**  (0 to 0 ) | ⨁⨁⨁⨁ High |  |
| **cycle normalisation** | | | | | | | | | | | | |
| 6 | randomised trials | serious | very serious | not serious | not serious | publication bias strongly suspected | 121/215 (56.3%) | 86/209 (41.1%) | **RR 1.42** (0.80 to 2.53) | **173 more per 1 000** (from 82 fewer to 630 more) | ⨁◯◯◯ Very low |  |
| **pregnancy rate** | | | | | | | | | | | | |
| 5 | randomised trials | serious | serious | not serious | extremely serious | publication bias strongly suspected | 60/193 (31.1%) | 47/190 (24.7%) | **RR 1.22** (0.84 to 1.78) | **54 more per 1 000** (from 40 fewer to 193 more) | ⨁◯◯◯ Very low |  |
| **Glucose (assessed with: mg/dl)** | | | | | | | | | | | | |
| 5 | randomised trials | not serious | not serious | not serious | very serious | none | 186 | 187 | - | **0**  (0 to 0 ) | ⨁⨁◯◯ Low |  |
| **Insulin (assessed with: µU/ml)** | | | | | | | | | | | | |
| 6 | randomised trials | not serious | not serious | not serious | not serious | none | 212 | 215 | - | **0**  (0 to 0 ) | ⨁⨁⨁⨁ High |  |
| **HOMA index** | | | | | | | | | | | | |
| 6 | randomised trials | not serious | not serious | not serious | not serious | none | 210 | 209 | - | **0**  (0 to 0 ) | ⨁⨁⨁⨁ High |  |
| **Pregnancy rate 2** | | | | | | | | | | | | |
| 3 | randomised trials | serious | not serious | not serious | serious | publication bias strongly suspected | 33/93 (35.5%) | 22/90 (24.4%) | **RR 1.38** (0.88 to 2.15) | **93 more per 1 000** (from 29 fewer to 281 more) | ⨁◯◯◯ Very low |  |

**Supplementary results 1.:** **PCOS patomechanism**

In PCOS, hyperandrogenism influences insulin sensitivity and secretion, but it can also be affected by genetic predisposition, dietary habits, and lifestyle. (27) In PCOS a mixed hyperandrogenic state can be observed. The developing hyperinsulinemia increases the androgen production of the adrenal cortex induced by ACTH stimulus, it leads to elevated DHEA/ DHEA-S levels. Hyperinsulinemia also reduces the production of SHBG by the liver; causing elevated free testosterone levels. (28) In addition, hyperinsulinemia stimulates the androgen overproduction of ovarian theca cells, which results in higher total testosterone levels.(29) Moreover, increased level of LH in PCOS and insulin act synergistically on theca cells, which results more excessive amount of androgen production. (30)Elevated testosterone level is linked to adiposity, IR and higher glucose levels in women.(31)

That is why we can summarize that IR and hyperandrogenemia forms vicious cycle in the pathogenesis of PCOS.(32)


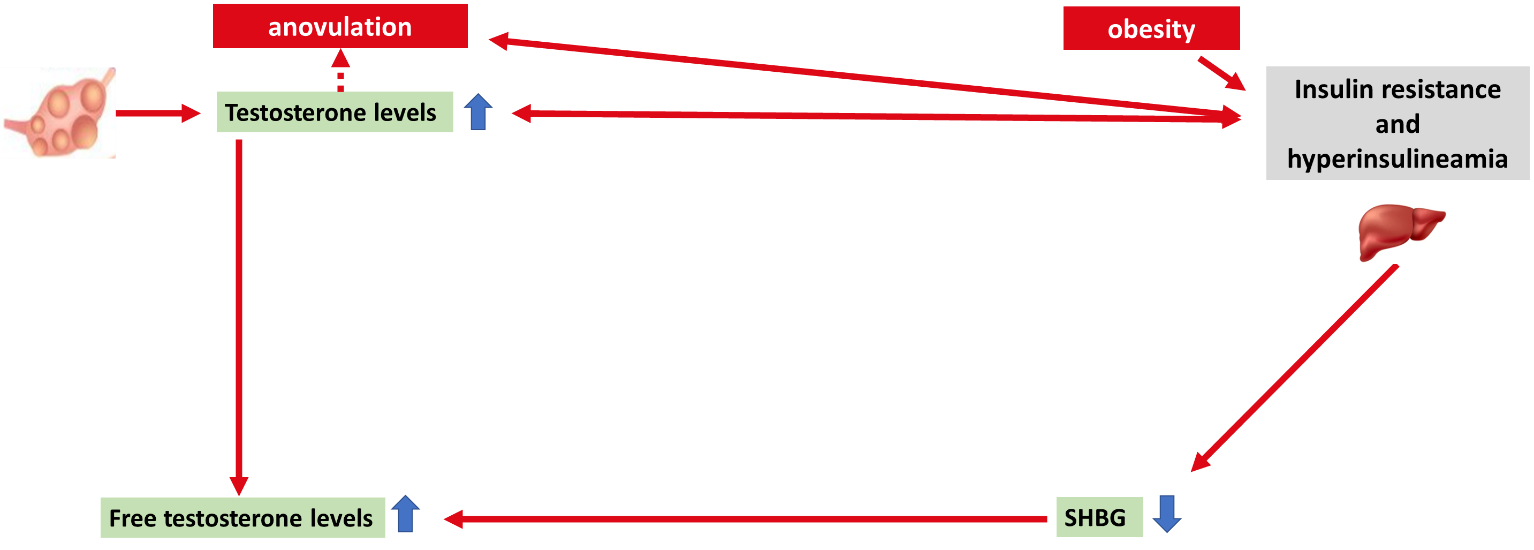


**vicious cycle**

**Supplementary results 2:** **FG-score**

We found only one article that compared the effect of inositols to placebo regarding hirsutism. Donne et al. suggested that inositol may have a beneficial effect on FG score. However, more studies are needed to strengthen this result. In this respect, inositols were even inferior to metformin. On the other hand, the average score of each RCT’s cohort varied between 7 and 18, showing a diverse population. The mean change induced by inositol or metformin was also diverse among studies between 0 and 2.5. These results may strongly depend on the study population and the inter-observer variability during the scoring process. However, the difference detected in these studies was clinically irrelevant.

**Supplementary results 3:Side effects**

Only two studies reported inositols having side effects. (1, 21) However, according to our pooled data, 7% of patients experienced bloating during inositol treatment compared to 53% in the metformin group.

**Supplementary results 4.: Pregnancy rate**

We did not find any significant effect of inositols on pregnancy rate. However, we found a serious risk of bias in reporting this outcome. For example, only one study evaluated how many women wished to be pregnant at the beginning of patient enrollment. (11)Moreover, they did not report if patients used any non-medical contraception methods.

**Supplementary results 5.:** **Different isomers of inositols**

We aimed to analyze the effects of different inositol isomers in treating PCOS. However, due to the low number of RCTs, we could not provide convincing evidence for the superiority of one of the isomers or the combinations. On the other hand, the combination of DCI and myoinositol seems to have less efficacy than monotherapy, although we found only one eligible RCT per outcome with combined therapy. DCI is known to have an aromatase inhibitory effect that may worsen PCOS symptoms in case of long-term therapy. However, it may improve insulin resistance in the short term, thus increasing SHBG production.**(33)** According to our data, it had no hyperandrogenic effect during the 6-8 week-long treatment. Myoinositol is the most used inositol in RCTs, so we analyzed its effects compared to placebo and metformin. Based on our results, myoinositol reduced fasting glucose, androstenedione levels, and BMI compared to placebo. It was non-inferior to metformin in any outcomes, except FG-score, and it was even more effective in the elevation of SHBG levels.

**REFERENCES**

1. Angik R JSSHC, Chimote A. A comparative study of metabolic and hormonal effects of myoinositol vs. metformin in women with polycystic ovary syndrome: a randomised controlled trial. International Journal of Reproduction, Contraception, Obstetrics and Gynecology. 2015;4(1):189-94.

2. Benelli E, Del Ghianda S, Di Cosmo C, Tonacchera M. A Combined Therapy with Myo-Inositol and D-Chiro-Inositol Improves Endocrine Parameters and Insulin Resistance in PCOS Young Overweight Women. International journal of endocrinology. 2016;2016.

3. Brusco GF, Mariani M. Inositol: effects on oocyte quality in patients undergoing ICSI. An open study. European review for medical and pharmacological sciences. 2013;17(22):3095‐102.

4. Chirania K. MS, Behera S. A randomised clinical trial comparing myoinositol and metformin in PCOS. International journal of Reproduction, Contraception, Obstetrics and Gynecology. 2017;6(5):1814-20.

5. Chhabra N, Malik S. Effect of insulin sensitizers on raised serum anti-mullerian hormone levels in infertile women with polycystic ovarian syndrome. Journal of human reproductive sciences. 2018;11(4):348‐52.

6. Costantino D, Minozzi G, Minozzi F, Guaraldi C. Metabolic and hormonal effects of myo-inositol in women with polycystic ovary syndrome: A double-blind trial. European Review for Medical and Pharmacological Sciences. 2009;13(2):105-10.

7. Donà G, Sabbadin C, Fiore C, Bragadin M, Giorgino FL, Ragazzi E, et al. Inositol administration reduces oxidative stress in erythrocytes of patients with polycystic ovary syndrome. European journal of endocrinology. 2012;166(4):703‐10.

8. Donne MLE, Metro D, Alibrandi A, Papa M, Benvenga S. Effects of three treatment modalities (diet, myoinositol or myoinositol associated with D-chiro-inositol) on clinical and body composition outcomes in women with polycystic ovary syndrome. European review for medical and pharmacological sciences. 2019;23(5):2293‐301.

9. Fruzzetti F, Perini D, Russo M, Bucci F, Gadducci A. Comparison of two insulin sensitizers, metformin and myo-inositol, in women with polycystic ovary syndrome (PCOS). Gynecological endocrinology. 2017;33(1):39‐42.

10. Genazzani AD, Lanzoni C, Ricchieri F, Jasonni VM. Myo-inositol administration positively affects hyperinsulinemia and hormonal parameters in overweight patients with polycystic ovary syndrome. Gynecological endocrinology. 2008;24(3):139‐44.

11. Gerli S, Papaleo E, Ferrari A, Di Renzo GC. Randomized, double blind placebo-controlled trial: effects of myo-inositol on ovarian function and metabolic factors in women with PCOS. European review for medical and pharmacological sciences. 2007;11(5):347‐54.

12. Jamilian H, Jamilian M, Foroozanfard F, Afshar Ebrahimi F, Bahmani F, Asemi Z. Comparison of myo-inositol and metformin on mental health parameters and biomarkers of oxidative stress in women with polycystic ovary syndrome: a randomized, double-blind, placebo-controlled trial. Journal of psychosomatic obstetrics and gynaecology. 2018;39(4):307‐14.

13. Iuorno MJ, Jakubowicz DJ, Baillargeon JP, Dillon P, Gunn RD, Allan G, et al. Effects of d-chiro-inositol in lean women with the polycystic ovary syndrome. Endocrine practice. 2002;8(6):417‐23.

14. Vincenzo De Leo* MCM, Valentina Cappelli, Alessandra Di Sabatino, Claudia Tosti and Paola Piomboni. A Combined Treatment with Myo-Inositol and Monacolin K Improve the Androgen and Lipid Profiles of Insulin-Resistant PCOS Patients. Journal of Metabolic Syndrome. 2013;2:127.

15. Jamilian M, Farhat P, Foroozanfard F, Afshar Ebrahimi F, Aghadavod E, Bahmani F, et al. Comparison of myo-inositol and metformin on clinical, metabolic and genetic parameters in polycystic ovary syndrome: a randomized controlled clinical trial. Clinical endocrinology. 2017;87(2):194‐200.

16. Nehra J, Kaushal J, Singhal SR, Ghalaut VS. Comparision of myo-inositol versus metformin on anthropometric parameters in polycystic ovarian syndrome in women. International journal of pharmacy and pharmaceutical sciences. 2017;9(4):144‐8.

17. Nehra J, Kaushal J, Singhal SR, Ghalaut VS. A comparative study of myo inositol versus metformin on biochemical profile in polycystic ovarian syndrome in women. International journal of pharmaceutical sciences and research. 2017;8(4):1664‐70.

18. Nestler JE, Jakubowicz DJ, Reamer P, Gunn RD, Allan G. Ovulatory and metabolic effects of D-chiro-inositol in the polycystic ovary syndrome. New England journal of medicine. 1999;340(17):1314‐20.

19. Pourghasem S, Bazarganipour F, Taghavi SA, Kutenaee MA. The effectiveness of inositol and metformin on infertile polycystic ovary syndrome women with resistant to letrozole. Archives of gynecology and obstetrics. 2019;299(4):1193‐9.

20. Raffone E, Rizzo P, Benedetto V. Insulin sensitiser agents alone and in co-treatment with r-FSH for ovulation induction in PCOS women. Gynecol Endocrinol. 2010;26(4):275-80.

21. Rajasekaran K, Malhotra N, Mahey R, Khadgawat R, Kalaivani M. Myoinositol versus metformin pretreatment in GnRH-antagonist cycle for women with PCOS undergoing IVF: a double-blinded randomized controlled study. Gynecol Endocrinol. 2021:1-8.

22. Schillaci R, Mangione D, Lo Monte G, Vassiliadis A. Inositol supplementation and IVF outcome: Preliminary data. Italian Journal of Gynaecology and Obstetrics. 2012;24(1):38-44.

23. Shokrpour M, Foroozanfard F, Afshar Ebrahimi F, Vahedpoor Z, Aghadavod E, Ghaderi A, et al. Comparison of myo-inositol and metformin on glycemic control, lipid profiles, and gene expression related to insulin and lipid metabolism in women with polycystic ovary syndrome: a randomized controlled clinical trial. Gynecological endocrinology. 2019;35(5):406‐11.

24. Pooja Singh SB, Santosh Kumar Verma. A prospective randomised controlled study on the effects of myoinositol on ovarian functions and metabolic factors in women with polycystic ovarian syndrome. International Journal of Reproduction, Contraception, Obstetrics and Gynecology. 2020;9(12):4912-7.

25. Soldat-Stanković V, Popović-Pejičić S, Stanković S, Prtina A, Malešević G, Bjekić-Macut J, et al. The effect of metformin and myoinositol on metabolic outcomes in women with polycystic ovary syndrome: role of body mass and adiponectin in a randomized controlled trial. J Endocrinol Invest. 2021.

26. Tagliaferri V, Romualdi D, Immediata V, De Cicco S, Di Florio C, Lanzone A, et al. Metformin vs myoinositol: which is better in obese polycystic ovary syndrome patients? A randomized controlled crossover study. Clinical endocrinology. 2017;86(5):725‐30.

27. Jayasena CN, Franks S. The management of patients with polycystic ovary syndrome. Nature Reviews Endocrinology. 2014;10(10):624-36.

28. Armanini D, Boscaro M, Bordin L, Sabbadin C. Controversies in the Pathogenesis, Diagnosis and Treatment of PCOS: Focus on Insulin Resistance, Inflammation, and Hyperandrogenism. Int J Mol Sci. 2022;23(8).

29. Notaro ALG, Neto FTL. The use of metformin in women with polycystic ovary syndrome: an updated review. J Assist Reprod Genet. 2022;39(3):573-9.

30. Diamanti-Kandarakis E, Dunaif A. Insulin Resistance and the Polycystic Ovary Syndrome Revisited: An Update on Mechanisms and Implications. Endocrine Reviews. 2012;33(6):981-1030.

31. Lutz SZ, Wagner R, Fritsche L, Peter A, Rettig I, Willmann C, et al. Sex-Specific Associations of Testosterone With Metabolic Traits. Frontiers in Endocrinology. 2019;10.

32. Ding H, Zhang J, Zhang F, Zhang S, Chen X, Liang W, et al. Resistance to the Insulin and Elevated Level of Androgen: A Major Cause of Polycystic Ovary Syndrome. Front Endocrinol (Lausanne). 2021;12:741764.

33. Dinicola S, Unfer V, Facchinetti F, Soulage CO, Greene ND, Bizzarri M, et al. Inositols: From Established Knowledge to Novel Approaches. Int J Mol Sci. 2021;22(19).
